# Cove‐Edged Hexa‐*peri*‐hexabenzo‐bis‐*peri*‐octacene: Molecular Conformations and Amplified Spontaneous Emission

**DOI:** 10.1002/anie.202201088

**Published:** 2022-03-02

**Authors:** Yanwei Gu, Victor Vega‐Mayoral, Saül Garcia‐Orrit, Dieter Schollmeyer, Akimitsu Narita, Juan Cabanillas‐González, Zijie Qiu, Klaus Müllen

**Affiliations:** ^1^ Synthetic Chemistry Max Planck Institute for Polymer Research Ackermannweg 10 55128 Mainz Germany; ^2^ Madrid Institute for Advanced Studies IMDEA Nanociencia c/Faraday 9, Campus de Cantoblanco 28049 Madrid Spain; ^3^ Department of chemistry Johannes Gutenberg University Mainz Duesbergweg 10–14 55128 Mainz Germany; ^4^ Institute for Physical Chemistry Johannes Gutenberg University Mainz Duesbergweg 10–14 55128 Mainz Germany

**Keywords:** Amplified Spontaneous Emission, Cove-Edges, Molecular Conformations, Nanographenes

## Abstract

The bottom‐up synthesis of an unprecedentedly large cove‐edged nanographene, hexa‐peri‐hexabenzo‐bis‐peri‐octacene (**HBPO**), is reported in this work. Chiral high‐performance liquid chromatography and density functional theory (DFT) calculations revealed multiple conformations in solution. Two different molecular conformations, “waggling” and “butterfly”, were found in crystals by X‐ray crystallography, and the selectivity of conformations could be tuned by solvents. The optoelectronic properties of **HBPO** were investigated by UV/Vis absorption and fluorescence spectroscopies, cyclic voltammetry, and DFT calculations. The contorted geometry and branched alkyl groups suppress the aggregation of **HBPO** in solution, leading to a high fluorescence quantum yield of 79 %. The optical‐gain properties were explored through transient absorption and amplified spontaneous emission spectroscopies, which enrich the choices of edge structures for potential applications in laser cavities.

## Graphical abstract

An unprecedentedly large cove‐edged nanographene (NG), hexa‐*peri*‐hexabenzo‐bis‐*peri*‐octacene (**HBPO**), was synthesized. Different ratios of contorted conformations, “waggling” and “butterfly”, were found in the crystals. The amplified spontaneous emission, which was previously only reported for zigzag‐edged NGs, can be observed in this cove‐edged NG, thus enriching the choices of edge structures for potential applications in laser cavities.

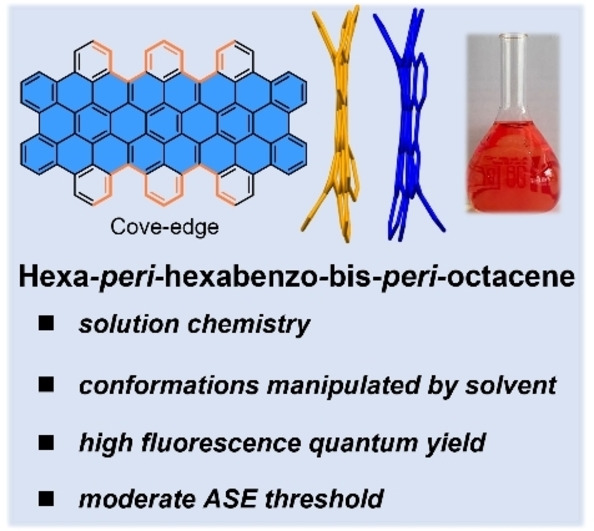

## Introduction

**Figure 1:**
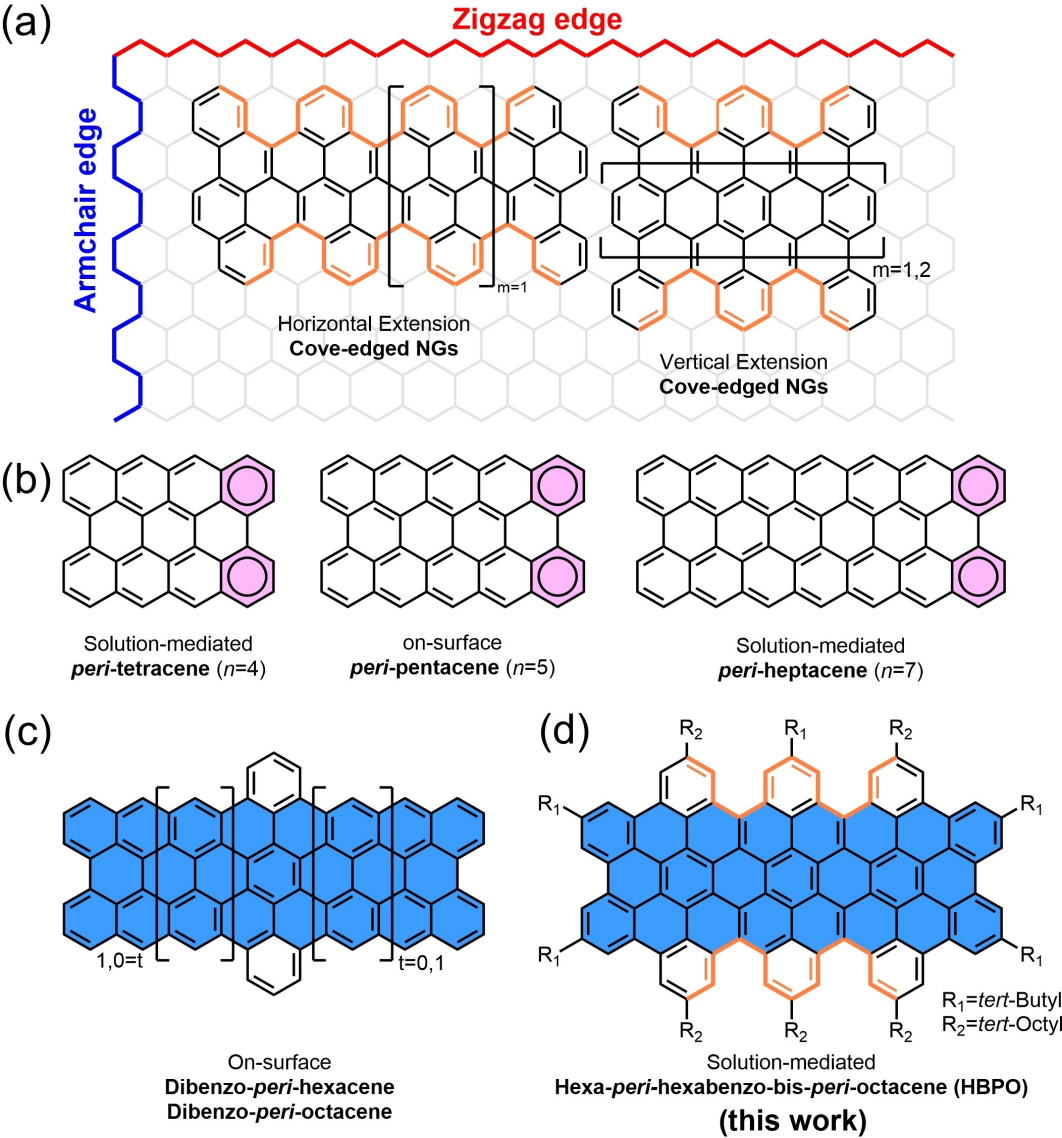
a) Representative examples of cove‐edged nanographenes (NGs). b) Reported structures of [*n*]periacenes. c) Dibenzo‐fused *peri‐*acenes achieved by on‐surface synthesis. d) Structure of hexa‐*peri*‐hexabenzo‐bis‐*peri*‐octacene (**HBPO**) in this work.

Polycyclic aromatic hydrocarbons (PAHs) have played an important role in the development of modern organic chemistry. Largely extended PAHs, which are also known as nanographenes (NGs), can be regarded as molecularly defined subunits of graphene[^1^, ^2^] and possess intriguing optical and charge‐transport properties.[^3^, ^4^, ^5^, ^6^] Different from graphene with a zero band gap, NGs display tunable energy gaps by structure modifications.[^7^, ^8^, ^9^, ^10^] Besides size and topology, the optoelectronic properties of NGs depend strongly on their edge structures. Zigzag‐ and armchair‐edges are the most widely studied cases (Figure 1a), which usually result in planar π‐conjugated systems.^[11]^ In contrast, cove‐ and fjord‐edges can strongly distort the π‐conjugated systems from planarity, owing to the steric repulsion between peripheral hydrogens.[^12^, ^13^, ^14^, ^15^] The resulting contorted structure has a strong influence on the packing behavior and the molecular conformations in the solid‐state.^[12]^ Compared with planar structures, twisted NGs reveal improved solubility and less pronounced aggregation.^[12]^ Until now, however, there are only limited reports on the solution‐mediated synthesis of cove‐edged NGs with a horizontal or vertical extension of π‐conjugated systems (Figure 1a).[^13^, ^14^]

[*n*]*peri*‐Acenes are a unique class of NGs composed of two armchair‐edges and two zigzag‐edges (Figure 1b), which are typically described with two aromatic sextet rings (highlighted with circles and pink color, Figure 1b) in the closed‐shell form. Thanks to the zigzag‐edges, [*n*]*peri*‐acenes display dramatically decreased energy gaps when extending the conjugation length [*n*] and can even show open‐shell diradical character if [*n*] exceeds a certain threshold (*n*>3, Figure 1b).[^16^, ^17^, ^18^] Three members, *peri*‐tetracene, *peri‐*pentacene, and *peri*‐heptacene, have been obtained recently and characterized via solution‐mediated or surface‐assisted synthesis, displaying diradical or even tetraradical character because of the aromatic stabilization from additional aromatic sextet rings in their open‐shell forms.[^17^, ^19^, ^20^] The surface‐assisted syntheses of dibenzo‐fused *peri*‐hexacene and dibenzo‐fused *peri*‐octacene have also been reported (Figure 1c), although, in a strict sense, they can no longer be considered as *peri*‐acenes.^[21]^ The adopted protocols rested upon a dibenzo‐*peri*‐fusion at zigzag‐edges of *peri*‐acenes and furnished increased energy gaps. With the assistance of more *peri*‐fused benzene rings, zigzag‐edges of *peri‐*acenes can be transformed into cove‐edges, thus providing access to stabilized congeners.

Herein, the cove‐edged hexa‐*peri*‐hexabenzo‐bis‐*peri*‐octacene (**HBPO**, Figure 1d) was designed by *peri*‐fusing six benzene rings to the two zigzag‐edges of *peri*‐octacene. To the best of our knowledge, this is the largest NG with a benzo‐fused *peri*‐acene backbone achieved by solution chemistry.[^21^, ^22^] The effect of *peri*‐fused benzene rings on the electronic structure of *peri*‐octacene was investigated experimentally by UV/Vis absorption spectroscopy and theoretically by density functional theory (DFT) calculations. Remarkably enough, two different contorted conformations of **HBPO**, “waggling” and “butterfly”, were confirmed by X‐ray crystallography, and the selectivity of conformations in the crystalline form can be manipulated by the choice of the solvents. Ultrafast transient absorption (TA) and amplified spontaneous emission (ASE) spectroscopies of **HBPO** revealed the potential of cove‐edged NGs as optical gain media for laser cavities, enriching the choices of edge structures besides the previously known zigzag periphery.[^23^, ^24^, ^25^]

## Results and Discussion

**Scheme 1:**
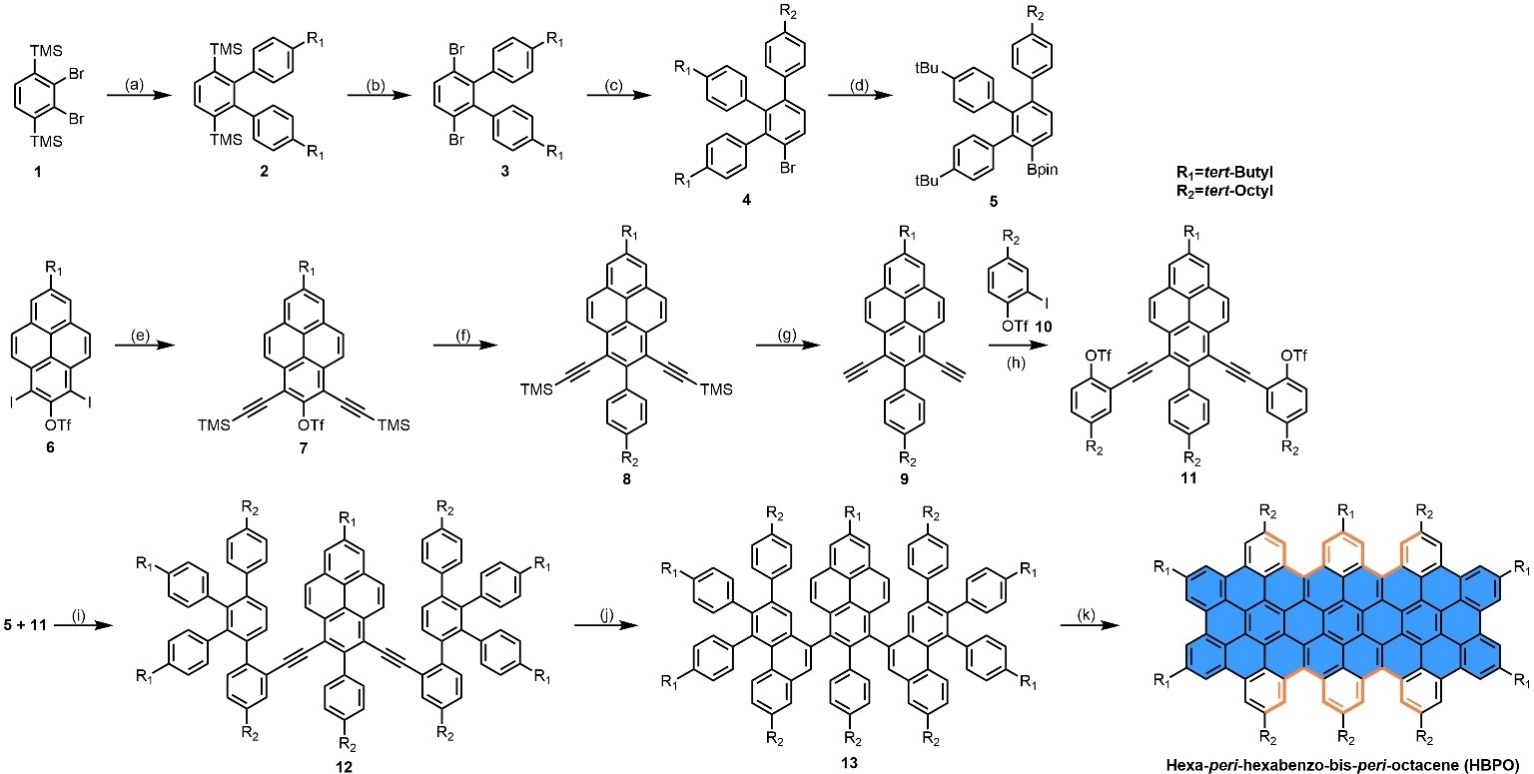
Synthetic route for hexa‐*peri*‐hexabenzo‐bis‐*peri*‐octacene (**HBPO**). Reagents and conditions: a) 4‐*tert*‐butylphenylboronic acid, K_3_PO_4_, *N,N*‐dimethylformamide, water, Pd(dppf)Cl_2_⋅CH_2_Cl_2_, 90 °C, 12 h, 80 %; b) bromine, dichloromethane, methanol, room temperature (R.T.), 12 h, 70 %; c) 4‐*tert*‐octylphenylboronic acid pinacol ester, Pd(PPh_3_)_4_, K_2_CO_3_, tetrahydrofuran (THF), water, 80 °C, 12 h, 44 %; d) *n*‐BuLi, 2‐isopropoxy‐4,4,5,5‐tetramethyl‐1,3,2‐dioxaborolane, THF, −78 °C to R.T., 12 h, 68 %; e) trimethylsilylacetylene, CuI, Pd(PPh_3_)_2_Cl_2_, triethylamine, 50 °C, 12 h, 67 %; f) 4‐*tert*‐octylphenylboronic acid pinacol ester, K_3_PO_4_, Pd(PPh_3_)_4_, toluene, ethanol, water, 105 °C, 12 h, 77 %; g) tetra‐*n*‐butylammonium fluoride, THF, R.T., 1 h, 92 %; h) Pd(PPh_3_)_2_Cl_2_, CuI, triethylamine, R.T., 12 h, 70 %; i) Pd(PPh3)_4_, K_2_CO_3_, dioxane, water, 95 °C, 12 h, 96 %; j) InCl_3_, mesitylene, 150 °C, 12 h, 90 %; k) 2,3‐dichloro‐5,6‐dicyano‐1,4‐benzoquinone, triflic acid, dichloromethane, 0 °C to R.T., 30 mins, 46 %. Pd(dppf)Cl_2_⋅CH_2_Cl_2_: [1,1′‐bis(diphenylphosphino)ferrocene] dichloropalladium(II) complex with dichloromethane, Pd(PPh_3_)_4_: tetrakis(triphenylphosphine)palladium(0), Pd(PPh_3_)_2_Cl_2_: bis(triphenylphosphine)palladium(II) dichloride.

The synthesis of **HBPO** is depicted in Scheme 1. The most simple molecule with a single cove‐edge is the [4]helicene, which can be constructed through the Mallory photocyclization^[26a]^ or transition‐metal catalyzed annulation of alkynes,^[26b]^ while multiple cove‐edges can be built up in one step by cyclodehydrogenations^[27]^ of polyphenylene derivatives. Therefore, from a retrosynthesis point of view, precursor **13** was designed by opening multiple C−C bonds between aromatic rings. Two building blocks, boronate ester **5** and triflate **11**, were first prepared by multi‐step syntheses with *tert*‐butyl groups and *tert*‐octyl groups, endowing good solubility to the target molecules. Subsequently, the three‐step protocol of Suzuki coupling‐benzannulation‐cyclodehydrogenation was utilized to build up the cove‐edges of **HBPO**. The treatment of key intermediate **12** with a catalytical amount of InCl_3_ furnished the corresponding **13** with a high yield of 90 % compared with other reported cyclization methods.[^28^, ^29^, ^30^] The structure of **13** was confirmed by ^1^H NMR, ^13^C NMR, high‐resolution mass spectrum (HRMS), and UV/Vis absorption spectroscopy. Notably, the InCl_3_‐catalyzed alkyne benzannulation proceeded regioselectively toward the tetraphenylbenzene units rather than the phenyl unit, presumably because of higher electron density at the reactive positions of the tetraphenylbenzene moiety. Finally, the fully fused **HBPO** was achieved through oxidative cyclodehydrogenation with 2,3‐dichloro‐5,6‐dicyano‐benzoquinone and triflic acid in a moderate yield of 46 %, without undesired rearrangement that sometimes occurred in the Scholl reaction.^[31]^

**HBPO** exhibited excellent solubility (4 mg mL^−1^) in chloroform and toluene, and moderate solubility (1 mg mL^−1^) in other common organic solvents, such as dichloromethane and tetrahydrofuran (THF), thus allowing unambiguous structure characterization. The sharp aromatic proton NMR signals of **HBPO** (THF‐*d*_8_/CS_2_=2/1, room temperature, Figure S34) indicated great solubility of **HBPO** in this mixed solvent without pronounced aggregation. All proton signals were clearly assigned with the assistance of correlation spectroscopy and rotating‐frame nuclear Overhauser effect spectroscopy (Figure S36–S38). The HRMS of **HBPO** recorded by atmospheric pressure chemical ionization displayed a parent ion peak at *m*/*z*=1904.1802, in accordance with the mass calculated for the molecular composition (calcd. for C_146_H_151_, *m*/*z*=1904.1810 [*M*+H]^+^, Figure S39).

The conformations of cove‐edged NGs could be manipulated by the size of the substituents.^[12]^ Chiral high‐performance liquid chromatography (HPLC) separation of **HBPO** indeed proved the existence of two isomers in a ratio of approximately 2 : 1, although the partly overlapping peaks prevented further investigations (Figure S6). Three possible conformations were identified theoretically for **HBPO** and noted as “waggling”, “butterfly”, and “helical” (Figure S7a). The DFT calculations suggest that the total energy of the butterfly conformation is 2.2 kcal mol^−1^ higher than that of the waggling one, while the total energy of the helical form is much higher (43.7 kcal mol^−1^), therefore the helical conformation is not expected to be observed experimentally (Figure S7a). The activation energies for conformational isomerism were calculated by DFT to be 50.9/50.8 kcal mol^−1^ starting from waggling/butterfly states, excluding their interconversion at room temperature. The simulated isomerization barriers for waggling and butterfly conformations of the unsubstituted core, however, were dramatically reduced to 11.9/12.1 kcal mol^−1^ (Figure S7b), indicating significant steric repulsions at the cove‐edges caused by the branched alkyl chains in **HBPO**. Similarly, previous reports of fjord‐edged^[15]^ and armchair‐edged graphene nanoribbons^[32]^ emphasized the effect of branched alkyl chains at the periphery by enhancing the backbone distortion. Therefore, **HBPO** provides a new opportunity to increase the racemization barriers and prevent the rapid interconversion in smaller helical molecules (e.g. 4‐ and 5‐helicenes).^[33]^

**Figure 2:**
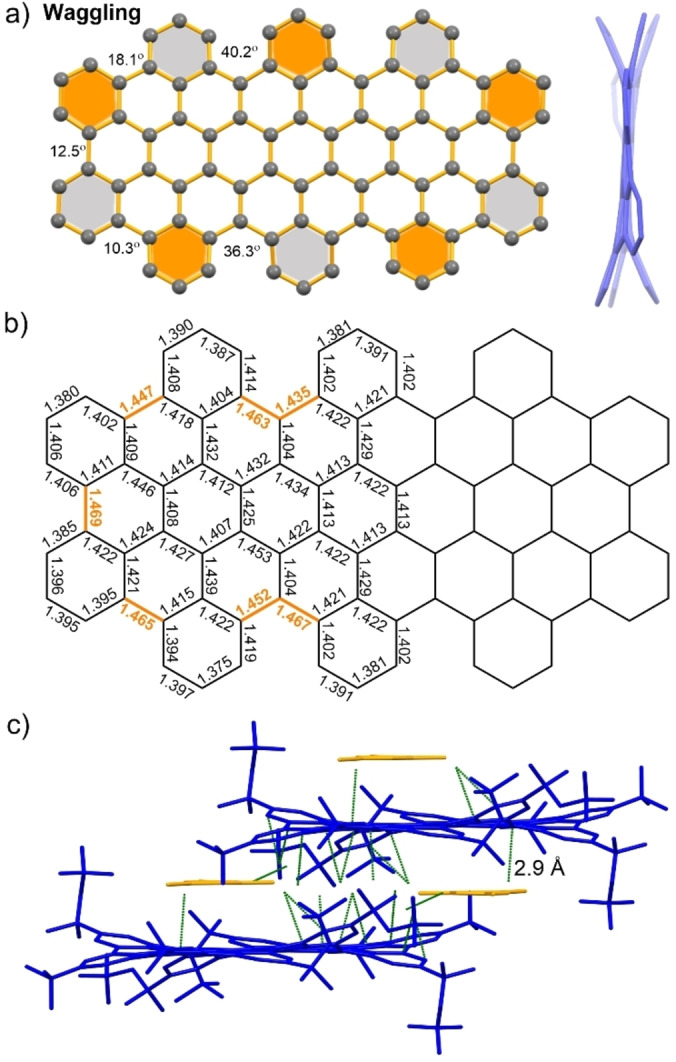
a) Top‐down and side views (H atoms and alkyl groups omitted for clarity) of the waggling conformation of **HBPO** crystal structure grown from mesitylene/methanol. The benzene rings along the cove‐edges and bay regions are bent above (orange color) or below (grey color) the central benzene ring. b) Selected bond lengths (in Å) of **HBPO**. c) Waggling conformation of **HBPO** with only close C−H⋅⋅⋅π contacts (green color, H atoms omitted for clarity, mesitylene labeled with orange color).

The molecular conformations of **HBPO** were further proven by single‐crystal X‐ray diffraction analysis after crystal growth by slow solvent diffusion of methanol into its mesitylene solution.^[34]^ Notably, the crystal structure displayed the single waggling conformation with the lowest total energy, where six benzene rings adopted an “up‐down” arrangement of the inner cove edges with different torsional angles (36.3° and 40.2°) between adjacent benzenoid rings (Figure 2a). Besides, the neighboring benzene rings at the bay regions also displayed “up‐down” torsional angles of 10.3°, 12.5°, and 18.1° because of the waggling backbone and branched alkyl chains. Correspondingly, these torsional angles at the cove edges and bay regions led to much longer C−C bonds (1.44–1.47 Å, highlighted in orange color, Figure 2b).

**Figure 3:**
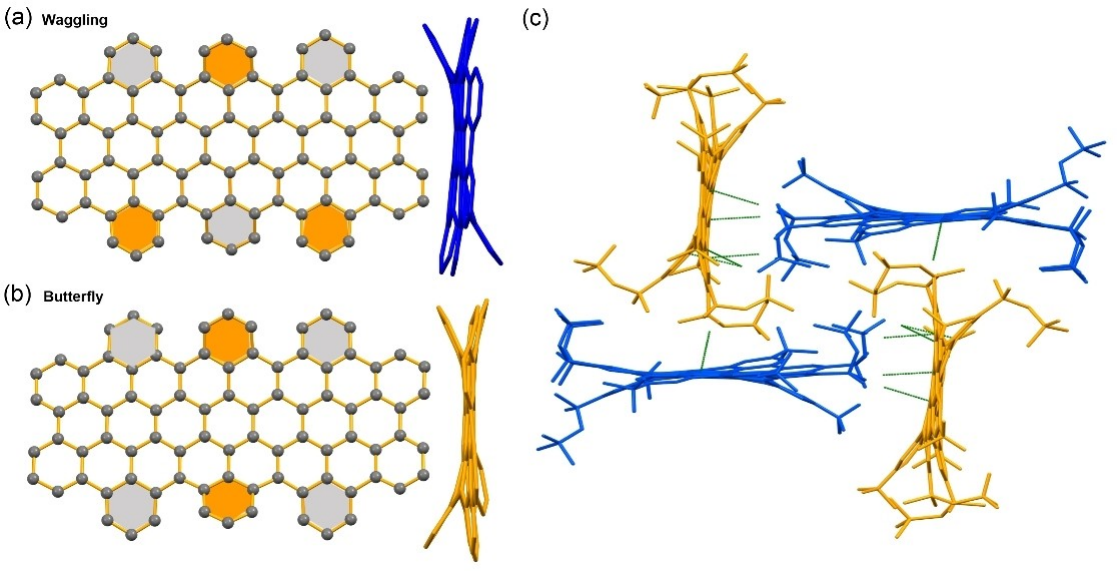
Top‐down and side views (H atoms and alkyl groups omitted for clarity) of a) the waggling (blue color) and b) the butterfly (yellow color) conformations of **HBPO** crystal structure grown from toluene/methanol. The benzene rings along the cove‐ edges are bent above (orange color) or below (grey color) the bis‐*peri*‐octacene backbone. c) Two conformations of **HBPO** with only close C−H⋅⋅⋅π contacts (green color, H atoms omitted for clarity).

Remarkably, two different conformations of **HBPO**, waggling and butterfly, were found to coexist in a 1 : 1 ratio in the crystal structure grown from slow solvent diffusion of methanol into its toluene solution.^[34]^ Both conformations displayed contorted frameworks because of the steric repulsion between the six “up‐down” alternating benzene rings along the cove‐edges (Figure 3a, b). Unfortunately, the bond length analysis of **HBPO** in this crystalline form is not accessible due to the poor diffraction and highly disordered side‐groups. The molecular packing fails to reveal tight intermolecular π–π interactions, but only close contacts between the aliphatic chains and the π‐surface via [C−H⋅⋅⋅π] interactions, indicating that aliphatic chains play a crucial role in crystal packing.^[35]^ The presence of two different conformations in one single crystal is rare for PAHs and NGs.^[36]^ In the present case, **HBPO** exhibited very different crystallization behavior during the crystal growth process in different solvent systems (mesitylene/methanol or toluene/methanol), where solvents served as the guest molecules to profoundly influence the molecular organization of difference conformations.

**Figure 4:**
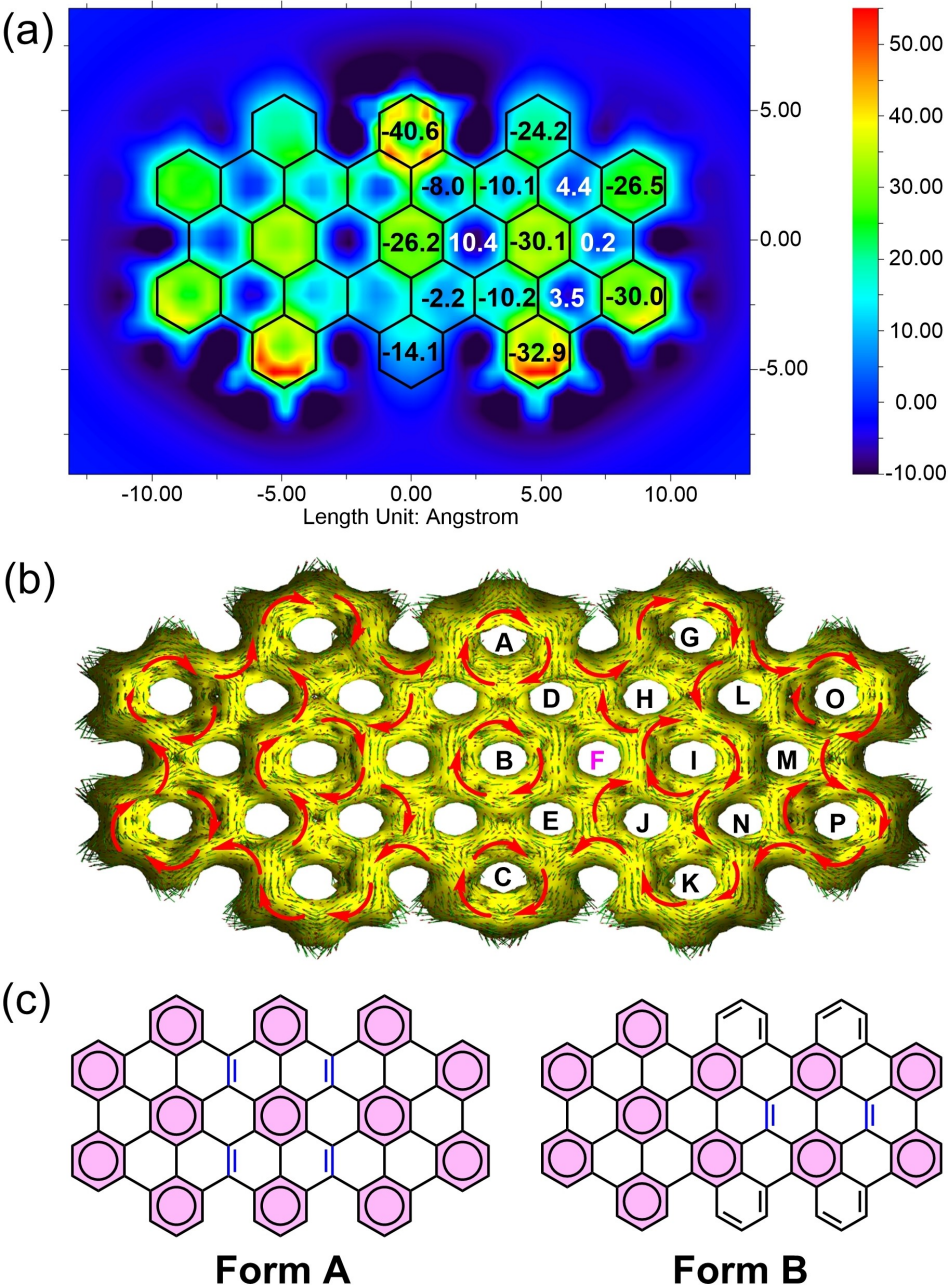
a) ICSS plot at 1 Å of *Z*‐axis (ICSS(1)_zz_) and NICS(1)_zz_ values for **HBPO** with waggling conformation. Z‐axis perpendicular to drawing plane. Red and orange regions are correlated to strong aromaticity values. b) Calculated ACID plot (isovalue=0.04, contribution from π electrons only) of **HBPO** with waggling conformation. c) Two resonance structures of **HBPO**. Aromatic sextet rings are shown with circles and pink color.

Calculations of isotropic chemical shielding surfaces (ICSS),[^37^, ^38^] nucleus‐independent chemical shifts (NICS),^[39]^ and anisotropy of the induced current densities (ACID)^[40]^ of **HBPO** provide insights into its electronic structure and, in particular, aromaticity. Here, the waggling conformation with the lowest total energy was chosen as the model structure to conduct the calculations. The Multiwfn package^[38]^ was used to generate the ICSS(1)_zz_ plot (at 1 Å at *Z*‐axis) of **HBPO** in Figure 4a, where red and orange colors indicated high aromaticity. The NICS(1)_zz_ values in Figure 4c suggest two resonance structures for **HBPO**: 13 aromatic sextet rings with 4 localized double bonds (form A) and 11 aromatic sextet rings with 2 localized double bonds (form B), in agreement with the ACID calculation (Figure 4b). Due to the partial contribution of form B, π‐electrons can establish diatropic ring currents along the periphery, as well as through pathways across the inner benzenoid rings (G/H/I/J/K). The dominance of resonance form A is consistent with Clar's aromatic sextet rule.^[41]^ The NICS(1)_zz_ values of butterfly conformation (Figure S3) indicated the same conclusion of aromaticity and resonance form as those of waggling conformation. The ACID plots of *peri*‐octacene, dibenzo‐*peri*‐octacene, and **HBPO** (Figure 4b and S3) indicate that benzene rings fused at *peri*‐positions of the zigzag‐edges can disrupt the delocalized π‐conjugated system, thus leading to larger HOMO–LUMO energy gaps with more benzo‐fusions (*peri*‐octacene: 0.35 eV; dibenzo‐*peri*‐octacene 1.35 eV; **HBPO** 2.10 eV).

**Figure 5:**
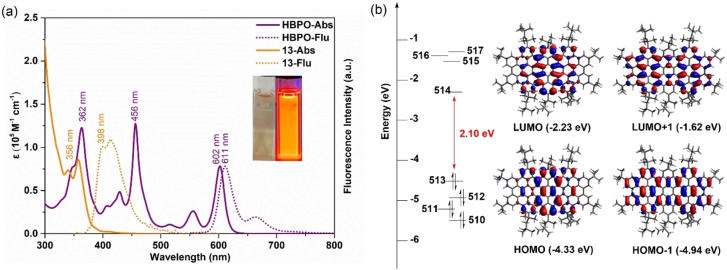
a) UV/Vis absorption and fluorescence spectra of **HBPO** and **13** solutions (*c*=2×10^−5^ M) at room temperature in toluene and dichloromethane, respectively. Inset: Photographs of **HBPO** in dilute toluene solution under ambient condition (left) and 365 nm UV excitation (right). b) Frontier molecular orbitals and energies of **HBPO** calculated at the B3LYP/6‐31G(d, p) level of theory.

The UV/Vis absorption spectrum of **HBPO** displayed three intense and well‐resolved absorption bands (Figure 5a), which were red‐shifted with respect to the band of precursor **13** at 356 nm, reflecting the extended π‐conjugation after cyclodehydrogenation. According to the time‐dependent density functional theory (TD‐DFT) calculations, the longest‐wavelength absorption band at 602 nm can be assigned to the HOMO→LUMO electronic transition (Figure 5b and Table S1). Notably, the HOMO and LUMO are highly localized in the central peropyrene substructure. The calculated energy gap of 2.10 eV is consistent with the optical band gap (2.01 eV) from the absorption spectrum and electrochemical energy gap (1.75 eV) measured by cyclic voltammetry (Figure S2). The small Stokes shift of 9 nm determined from the emission band at 611 nm indicated the rigid structure of **HBPO** which also exhibited a high fluorescence quantum yield of 79 % measured at a low concentration (absorbance<0.1) with a Nile blue A perchlorate standard. The emission can already be observed under indoor light excitation (Figure S1).

**Figure 6:**
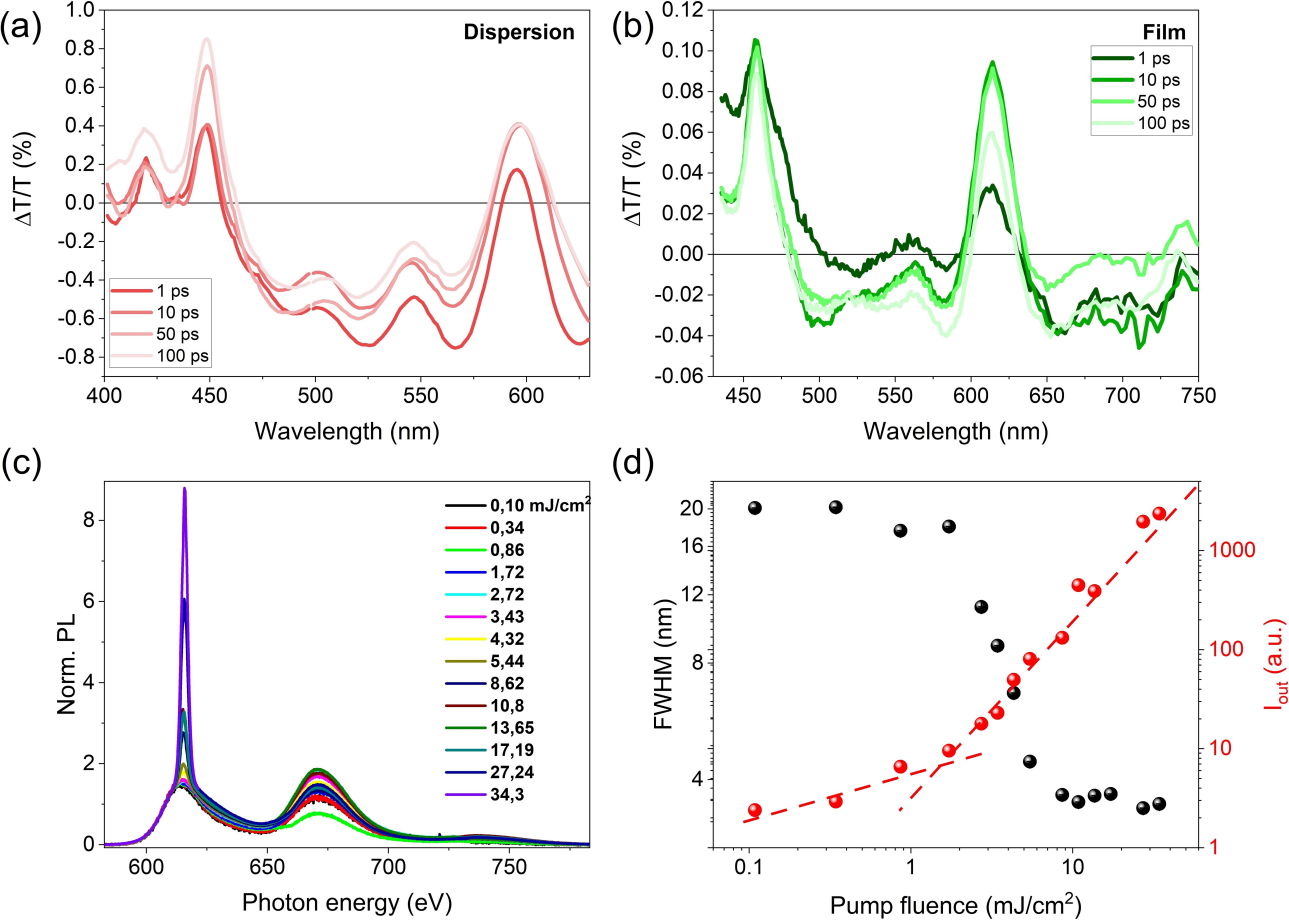
a, b) Transient absorption spectra of **HBPO** in chloroform and of a 1.24 wt % blend of **HBPO** in PS taken at four different pump‐probe time delays. c) Photoluminescence spectra taken with different pump fluences. d) Full width half maximum (black circles) and emission output (red circles) as a function of pump fluence in a 1.24 wt % blend of **HBPO** in PS.

Amplified spontaneous emission (ASE) is a process where the spontaneously emitted radiation (luminescence) is amplified due to the optical gain of the material. The presence of ASE in a material is a prerequisite for lasing. In recent years, ASE was reported in a few zigzag‐edged NGs with high fluorescence quantum yields[^23^, ^24^, ^25^] whereby bulky substituents are necessary to prevent the π–π stacking.^[42]^ However, ASE has not yet been investigated in NGs with other edge structures. The cove‐edged **HBPO** exhibits a contorted skeleton without strong‐intermolecular contacts in the crystal and high fluorescence quantum yield, thus prompting us to explore its possible ASE characteristics. Broadband TA spectroscopy of **HBPO** in chloroform (Figure 6a) displayed a negative Δ*T*/*T* band covering the 450–800 nm spectral window ascribed to excited‐state absorption (ESA) superposed with two overwhelmed bumps at 459 and 613 nm, which were tentatively assigned to ground state bleaching (GSB) and stimulated emission (SE) respectively (Figure S8). The absence of ASE in the solution was confirmed by the lack of emission linewidth narrowing upon pumping with the highest available fluences (Figure S9). In contrast, the TA spectra of the **HBPO**/polystyrene (PS) film exhibited three main spectral features (Figure 6b): (1) a positive Δ*T*/*T* band centered at 459 nm assigned to GSB, (2) SE centered at 613 nm, (3) negative Δ*T*/*T* between both spectral features and at longer wavelengths ascribed to ESA. Such enhanced performance in the doped film is likely resulting from less non‐radiative decay channels in the inert PS matrix film and thereof inhibition of the intermolecular charge‐transfer process. The latter is commonly observed in other luminescent conjugated polymers and graphene molecules.[^43^, ^44^, ^45^, ^46^] The presence of persistent SE for hundred picoseconds holds promise for the ASE of **HBPO** in the solid‐state.

To explore the gain properties, ASE measurements in the doped film were carried out upon pumping at 456 nm to yield stable and efficient ASE throughout the entire **HBPO**/PS film. The narrowing of the emission linewidth appeared at a pump fluence of 2.4 mJ cm^−2^ (Figure 6c) with homogeneous ASE action in different regions of the film with a similar threshold (Figure 6d). Although **HBPO** exhibits a higher ASE threshold compared with reported zigzag‐edged NGs,[^23^, ^24^, ^25^] this is the first observation of ASE behavior in a contorted NG with cove‐edges, therefore enriching the future molecular design options.

## Conclusion

In summary, we demonstrated an efficient approach toward the cove‐edged **HBPO**. Different from many other NGs, the title compound did not suffer from strong aggregation and therefore gave sharp NMR signals. Due to the hydrogen repulsion between *peri*‐fused benzene rings, waggling and butterfly conformations were confirmed experimentally and theoretically. X‐ray crystallography indicated the simultaneous occurrence of two molecular conformations with different contorted geometries. More importantly, the selectivity of conformations of **HBPO** can be manipulated by solvents in the crystalline form. The introduction of branched alkyl chains at the periphery increased the racemization barriers of unsubstituted aromatic core, which paves the way to prevent the rapid interconversion of small helical structures. The strong orange emission with a high fluorescence quantum yield of 79 % paves the way to obtain optical‐gain properties with a moderate threshold (2.4 mJ cm^−2^) by comparison with literature work on similar systems (Figure S13). The bent aromatic core together with the presence of branched alkyl chains secures good solution processability. Due to its ASE properties, **HBPO** holds promise as the gain medium in laser devices for future investigations.

## Conflict of interest

The authors declare no conflict of interest.

1

## Supporting information

As a service to our authors and readers, this journal provides supporting information supplied by the authors. Such materials are peer reviewed and may be re‐organized for online delivery, but are not copy‐edited or typeset. Technical support issues arising from supporting information (other than missing files) should be addressed to the authors.

Supporting Information

Supporting Information

Supporting Information

Supporting Information

Supporting Information

## Data Availability

The data that support the findings of this study are available from the corresponding author upon reasonable request.
