# Supplementary material for: Cove‐Edged Hexa‐peri‐hexabenzo‐bis‐peri‐octacene: Molecular Conformations and Amplified Spontaneous Emission
Source: Angew Chem Int Ed Engl. 2022 Mar 2;61(18):e202201088. doi: 10.1002/anie.202201088 (PMC9311809; doi:10.1002/anie.202201088)
Supplement: Supplementary file 5 — Supporting Information [file ANIE-61-0-s003.pdf]

Angewandte Chemie
Eine Zeitschrift der Gesellschaft Deutscher Chemiker

Supporting Information

Cove-Edged Hexa-*peri*-hexabenzo-bis-*peri*-octacene: Molecular Conformations and Amplified Spontaneous Emission

Y. Gu, V. Vega-Mayoral, S. Garcia-Orrit, D. Schollmeyer, A. Narita, J. Cabanillas-González, Z. Qiu*, K. Müllen**

Table of Contents

Table of Contents 2
1. Experimental Procedures 3
 1.1 General methods 3
 1.2 Synthetic details 4
2. Fluorescence measurement 11
3. Cyclic voltammetry of HBPO 11
4. DFT calculations 11
5. Transient Absorption Spectroscopy and Amplified Stimulated Emission Characterization of HBPO 14
6. X-ray Crystallographic data 17
7. NMR and Mass spectra 20
8. Cartesian coordinates of Waggling Conformation, Butterfly Confirmation, Helical Conformation, Transition States (TS1 and TS2) of HBPO, Dibenzo-peri-octacene and peri-Octacene 33
9. References 78

1. Experimental Procedures

1.1 General methods

All reactions with air- or moisture-sensitive compounds were carried out under argon atmosphere using standard Schlenk line techniques. Unless otherwise noted, all starting materials were purchased from commercial sources and used without further purification. All other reagents were used as received. Column chromatography was conducted with silica gel (grain size 0.04 – 0.063 mm) and thin-layer chromatography (TLC) was performed on silica gel-coated aluminum sheets with an F254 indicator. Nuclear magnetic resonance (NMR) spectra were recorded in methylene chloride-d_2, chloroform-*d,* or THF-d_8 on AVANCE 300 MHz and AVANCE 500 MHz Bruker spectrometers. The chemical shift was recorded in ppm and the following abbreviations were used to explain the multiplicities: s = singlet, d = doublet, t = triplet, m = multiplet, dd = doublet of doublets. Abbreviations of solvents: DMF = *N*,*N*-dimethylformamide, DCM = dichloromethane, THF = tetrahydrofuran. High-resolution mass spectra (HRMS) using atmospheric pressure chemical ionization (APCI) were recorded on a MicrOTOF-QII instrument. UV-Vis absorption spectra were taken on a Perkin-Elmer Lambda 900 spectrophotometer using a 10 mm quartz cell. The fluorescence quantum yield (Φ) was measured using Nile blue A perchlorate (in ethanol under air, $\Phi = 0.27$) as a reference.[1] Photoluminescence spectra were recorded on an FL3095SL spectrometer (J&M TIDAS 9.5, Germany). Cyclic voltammetry (CV) was performed on a WaveDriver 20 Bipotentiostat/Galvanostat (Pine Instruments Company) and measurements were carried out in DCM containing 0.1 M *n*-Bu_4NPF_6 as supporting electrolyte at room temperature (scan rate: 100 mV/s). A glassy carbon electrode was used as a working electrode, a platinum wire as a counter electrode, and a silver wire as a reference electrode. High-performance liquid chromatography (HPLC) analysis was performed on an Agilent 1200 Series equipped with the following modules: quaternary pump (G1311A 1100), manual sample injector (Rheodyne 7725i), column thermostat (G1316A 1200), and DAD detector (G1713B1200). The chiral column used was Daicel Chiralpak IE analytical column (4.6 × 250 mm) packed with amylose tris-(3,5-dichlorophenylcarbamate) immobilized on silica gel (5 μm). The column temperature was set at 20 ℃ and the flow was constant during the operation. Density functional theory calculations were conducted at the B3LYP/6-31G(d,p) level using Gaussian 09 software package.[2]

1.2 Synthetic details

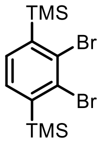

1

Compound **1** was synthesized by following the same procedure as reported before.[3]

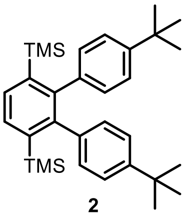

2

Compound **1** (0.76 g, 2 mmol), 4-*tert*-butylphenylboronic acid (1.07 g, 6 mmol, 3 equiv.), and potassium phosphate (2.54 g, 12 mmol, 6 equiv.) were mixed in a solution of DMF (15 mL) and water (2 mL). After the reaction mixture was bubbled under nitrogen for 15 mins, catalyst [1,1′ -bis(diphenylphosphino) ferrocene]dichloropalladium(II) complex with DCM (164 mg, 0.2 mmol, 10 mol%) was added in one portion. The mixture was heated to 90 ℃ and stirred overnight. After that, the black mixture was cooled to room temperature, 100 mL of ammonium chloride solution was added. The aqueous phase was extracted by ethyl acetate (3×50 mL). The combined organic phase was washed with brine and water and dried over sodium sulfate. After the solvents were removed by rotary evaporation, the residue was purified by silica gel chromatography using hexane as eluent, affording **2** as a colorless solid (0.78 g, 80%). ^{1}H NMR (300 MHz, methylene chloride-d_2): δ 7.59 (s, 2H), 7.07 (d, J = 7.8 Hz, 4H), 6.84 (d, J = 7.8 Hz, 4H), 1.21 (s, 18H), -0.08 (s, 18H). ^{13}C NMR (75 MHz, methylene chloride-d_2): δ 149.38, 147.91, 140.31, 139.83, 132.76, 130.97, 123.71 34.53, 31.40, 0.48. HRMS analysis (APCI): calcd. for $C_{32}H_{46}Si_2$ $[M]^+$: 486.3133; found: 486.3141 (error: -1.7 ppm).

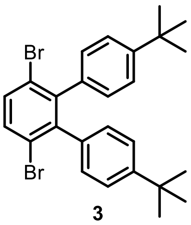

3

To a solution of compound **2** (4.87 g, 10 mmol) in methanol (59 mL) and DCM (59 mL) at 0 ℃, bromine (1.53 mL, 30.1 mmol) was added slowly. The mixture was stirred overnight at room temperature. The reaction was quenched by saturated aqueous sodium sulfite solution and extracted with DCM (2×50 mL). The combined organic phase was washed with brine and water and dried over sodium sulfate. After the solvents were removed by rotary evaporation, the residue was purified by silica gel chromatography using hexane as eluent, affording **3** as a colorless solid (3.5 g, 70%). ^{1}H NMR (300 MHz, methylene

chloride-d_2): δ 7.54 (s, 2H), 7.14 (d, J = 8.3 Hz, 4H), 6.85 (d, J = 8.4 Hz, 4H), 1.21 (s, 18H). ^{13}C NMR (75 MHz, methylene chloride-d_2): δ 150.28, 144.66, 137.55, 132.88, 129.91, 124.45, 123.45, 34.65, 31.31. HRMS analysis (APCI): calcd. for $C_{26}H_{28}Br_2$ $[M]^+$: 498.0552; found: 498.0553 (error: -0.1 ppm).

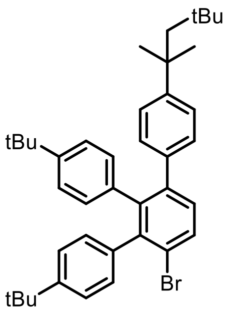

4

Compound **3** (3.70 g, 7.4 mmol), 4-*tert*-octylphenylboronic acid pinacol ester (3.05 g, 9.62 mmol, 1.3 equiv.), and potassium carbonate (5.11 g, 37 mmol, 5 equiv.) were mixed in a solution of THF (333 mL) and water (37 mL). After the reaction mixture was bubbled under nitrogen for 15 mins, catalyst tetrakis(triphenylphosphine)palladium(0) (212 mg, 0.185 mmol, 2.5 mol%) was added in one portion. The mixture was heated to 80 ℃ and stirred overnight. The reaction mixture was extracted by diethyl ether (2×50 mL). The combined organic phase was washed with brine and water and dried over sodium sulfate. After the solvents were removed by rotary evaporation, the residue was purified by silica gel chromatography using hexane as eluent, affording **4** as a colorless solid (1.95 g, 44%). ^{1}H NMR (300 MHz, methylene chloride-d_2): δ 7.70 (d, J = 8.3 Hz, 1H), 7.28 (d, J = 8.3 Hz, 1H), 7.16 (dd, J = 8.3, 4.5 Hz, 4H), 6.94 (dd, J = 10.6, 8.4 Hz, 4H), 6.85 (d, J = 8.3 Hz, 2H), 6.61 (d, J = 8.3 Hz, 2H), 1.66 (s, 2H), 1.29 (s, 6H), 1.24 (s, 9H), 1.12 (s, 9H), 0.61 (s, 9H). ^{13}C NMR (75 MHz, methylene chloride-d_2): δ 149.87, 148.87, 148.41, 142.95, 141.58, 131.49, 131.13, 130.63, 130.39, 129.43, 125.84, 124.35, 123.95, 123.25, 118.56, 57.18, 38.48, 34.65, 34.40, 32.56, 31.98, 31.67, 31.37, 31.28. HRMS analysis (APCI): calcd. for $C_{40}H_{49}Br$ $[M^+$: 608.3012; found: 608.3019 (error: -1.2 ppm).

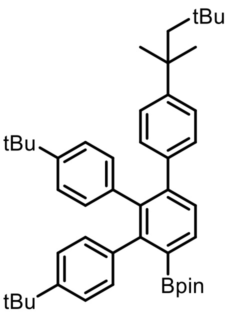

5

To a solution of compound **4** (1.95 g, 3.2 mmol) in THF (30 mL) at -78 ℃, *n*-BuLi (2.4 mL, 1.2 equiv., 1.6 M, 3.84 mmol) was added dropwise. The yellow solution was stirred at -78 ℃ for 1 hour. 2-Isopropoxy-4,4,5,5-tetramethyl-1,3,2-dioxaborolane (0.848 mL, 778 mg, 1.3 equiv., 4.16 mmol) was added dropwise

into the solution. The solution was warmed to room temperature and stirred overnight. After that, the reaction was quenched with water (100 mL). The aqueous phase was extracted by diethyl ether (3×50 mL). The combined organic phase was washed with water and brine and dried over sodium sulfate. After the solvents were removed by rotary evaporation, the residue was purified by silica gel chromatography using DCM/hexane (1:4) as eluent, affording **5** as a colorless solid (1.4 g, 68 %). ^{1}H NMR (300 MHz, methylene chloride-d_2): δ 7.62 (d, J = 7.6 Hz, 1H), 7.38 (d, J = 7.6 Hz, 1H), 7.13 (dd, J = 8.0, 5.8 Hz, 4H), 6.98 – 6.88 (m, 6H), 6.69 (d, J = 8.3 Hz, 2H), 1.67 (s, 2H), 1.29 (s, 6H), 1.25 (s, 9H), 1.16 (s, 9H), 1.09 (s, 12H), 0.64 (s, 9H). ^{13}C NMR (75 MHz, methylene chloride-d_2): δ 149.08, 148.59, 148.11, 146.99, 143.71, 140.06, 139.83, 139.49, 137.22, 132.42, 131.60, 130.42, 129.51, 128.59, 125.66, 123.99, 118.55, 83.79, 57.18, 38.46, 34.54, 34.43, 32.58, 32.01, 31.70, 31.47, 31.35, 24.71. HRMS analysis (APCI): calcd. for $C_{46}H_{62}BO_2$ $[M+H]^+$: 657.4845; found: 657.4841 (error: 0.6 ppm).

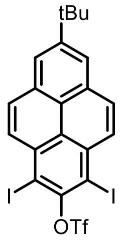

6

Compound **6** was synthesized by following the same procedure as reported before.[4]

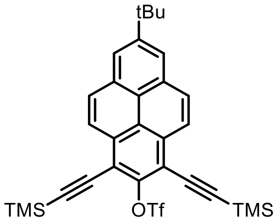

7

Compound **6** (658 mg, 1 mmol) and copper iodide (38 mg, 0.2 mmol, 20 mol%) were mixed in a solution of triethylamine (30 mL). After the reaction mixture was bubbled under nitrogen for 15 mins, catalyst bis(triphenylphosphine)palladium(II) dichloride (80 mg, 0.11 mmol, 11 mol%) was added in one portion. Then trimethylsilylacetylene (0.7 mL, 5 mmol, 5 equiv.) was added slowly to the reaction mixture, which was further stirred overnight at 50 ℃. The reaction mixture was then filtered through Celite. After the solvents were removed by rotary evaporation, the residue was purified by silica gel chromatography using DCM/hexane (1:9) as eluent, affording **7** as a colorless solid (400 mg, 67%). ^{1}H NMR (300 MHz, methylene chloride-d_2): δ 8.49 (d, J = 9.1 Hz, 2H), 8.36 (s, 2H), 8.26 (d, J = 9.1 Hz, 2H), 1.61 (s, 9H), 0.42 (s, 18H). ^{13}C NMR (75 MHz, methylene chloride-d_2): δ 151.20, 148.71, 133.52, 131.45, 131.10, 125.37, 125.15, 123.03, 121.61, 118.55, 111.93, 108.90, 96.74, 35.66, 31.91, -0.23, -2.06. HRMS analysis (APCI): calcd. for $C_{31}H_{34}F_2O_2SSi_2$ $[M+H]^+$: 599.1702; found: 599.1716 (error: -2.4 ppm).

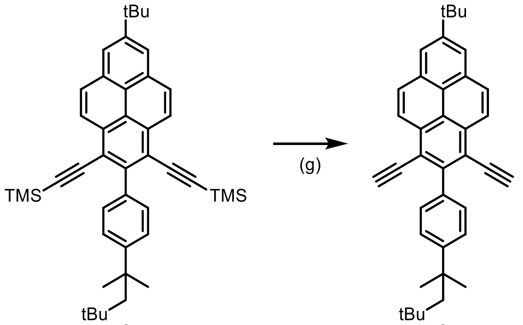

8 9

Compound **7** (750 mg, 1.25 mmol), 4-*tert*-octylphenylboronic acid pinacol ester (1.192 g, 3.75 mmol, 3 equiv.), and potassium phosphate (795 mg, 3.75 mmol, 3 equiv.) were mixed in a solution of toluene (12.5 mL), ethanol (1.25 mL), and water (1.25 mL). After the reaction mixture was bubbled under nitrogen for 15 mins, catalyst tetrakis(triphenylphosphine)palladium(0) (115 mg, 0.1 mmol, 8 mol%) was added in one portion. The mixture was heated to 105 ℃ and stirred overnight. The reaction mixture was extracted with diethyl ether (2×50 mL). The combined organic phase was washed with brine and water and dried over sodium sulfate. After the solvents were removed by rotary evaporation, the residue was purified by silica gel chromatography using DCM/hexane (1:9) as eluent, affording **8** as a yellow solid (620 mg, 77%). To a solution of compound **8** (620 mg, 0.97 mmol) in THF (20 mL) at 0 ℃, tetra-*n*-butylammonium fluoride (4 mL, 4 mmol, 1 M) was added slowly. The mixture was stirred at room temperature for 1 h. After the solvents were removed by rotary evaporation, the residue was purified by silica gel chromatography using DCM/hexane (1:9) as eluent, affording **9** as a yellow solid (440 mg, 92%). ^{1}H NMR (300 MHz, methylene chloride-d_2): δ 8.61 (d, J = 9.1 Hz, 2H), 8.34 (s, 2H), 8.24 (d, J = 9.2 Hz, 2H), 7.53 (d, J = 2.7 Hz, 4H), 3.49 (s, 2H), 1.85 (s, 2H), 1.61 (s, 9H), 1.49 (s, 6H), 0.79 (s, 9H). ^{13}C NMR (75 MHz, methylene chloride-d_2): δ 150.47, 149.75, 146.09, 137.40, 132.89, 131.13, 130.42, 130.09, 125.99, 125.76, 124.27, 123.39, 122.10, 116.63, 86.74, 81.50, 57.63, 38.87, 35.58, 32.73, 31.96, 31.71, 30.07. HRMS analysis (APCI): calcd. for $C_{38}H_{39}$ [M+H]$^+$: 495.3046; found: 495.3058 (error: -2.4 ppm).

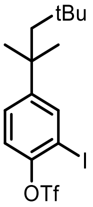

10

Compound **10** was synthesized by following the similar procedure as reported before.[4] ^{1}H NMR (300 MHz, chloroform-d): δ 7.86 (d, J = 2.2 Hz, 1H), 7.41 (dd, J = 8.7, 2.2 Hz, 1H), 7.22 (d, J = 8.7 Hz, 1H), 1.73 (s, 2H), 1.36 (s, 6H), 0.74 (s, 9H). ^{13}C NMR (75 MHz, chloroform-d): δ 152.75, 148.03, 139.60, 138.58, 128.06, 125.24, 121.13, 1210.00, 116.75, 112.50, 88.58, 57.00, 38.74, 32.55, 31.94, 31.42. HRMS analysis (APCI): calcd. for $C_{15}H_{20}F_3IO_3S$ [M]$^+$: 464.0130; found: 464.0137 (error: -1.6 ppm).

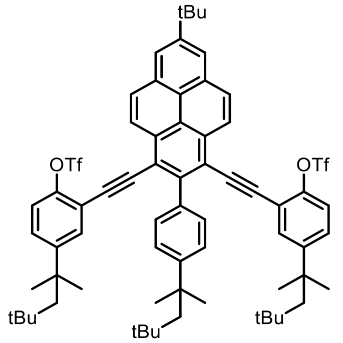

11

Compound **10** (658 mg, 1 mmol) and copper iodide (38 mg, 0.2 mmol, 20 mol%) were mixed in a solution of triethylamine (30 mL). After the reaction mixture was bubbled under nitrogen for 15 mins, catalyst bis(triphenylphosphine)palladium(II) dichloride (70 mg, 0.1 mmol, 10 mol%) was added in one portion. Then compound **9** was dissolved in degassed triethylamine (20 mL) and added slowly to the reaction mixture, which was further stirred overnight at room temperature. The reaction mixture was then filtered through Celite. After the solvents were removed by rotary evaporation, the residue was purified by silica gel chromatography using DCM/hexane (1:9) as eluent, affording **11** as a yellow solid (816 mg, 70%). ^{1}H NMR (300 MHz, methylene chloride-d_2): δ 8.80 (d, J = 9.1 Hz, 2H), 8.38 (s, 2H), 8.31 (d, J = 9.2 Hz, 2H), 7.80 (d, J = 8.3 Hz, 2H), 7.58 (d, J = 8.3 Hz, 2H), 7.51 – 7.39 (m, 4H), 7.28 (d, J = 8.6 Hz, 2H), 1.78 (d, J = 2.6 Hz, 6H), 1.62 (s, 9H), 1.47 (s, 6H), 1.39 (s, 12H), 0.77 (s, 18H), 0.60 (s, 9H). ^{13}C NMR (75 MHz, methylene chloride-d_2): δ 151.28, 150.55, 150.30, 147.58, 136.88, 133.05, 131.79, 131.28, 130.87, 130.58, 128.41, 126.16, 125.65, 124.44, 123.56, 122.21, 120.91, 117.57, 116.84, 93.85, 92.09, 57.37, 57.03, 38.98, 38.90, 35.60, 32.66, 32.51, 31.96, 31.78, 31.56. HRMS analysis (APCI): calcd. for $C_{68}H_{77}F_6O_6S_2$ [M+H]$^+$: 1167.5060; found: 1167.5076 (error: -1.4 ppm).

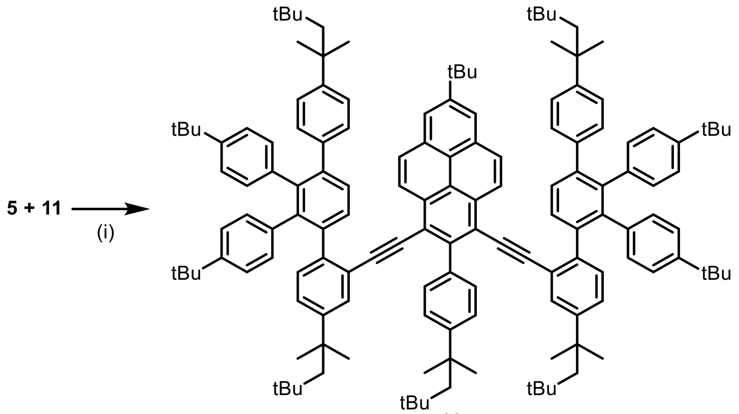

12

Compound **11** (475 mg, 0.4 mmol), compound **5** (695 mg, 1.04 mmol, 2.6 equiv.), and potassium carbonate (528 mg, 3.8 mmol, 9.5 equiv.) were mixed in a solution of dioxane (25 mL) and water (5 mL). After the reaction mixture was bubbled under nitrogen for 15 mins, catalyst tetrakis(triphenylphosphine)palladium(0) (230 mg, 0.2 mmol, 50 mol%) was added in one portion. The mixture was heated to 95 ℃ and stirred overnight. The reaction mixture was extracted by diethyl ether

(2×50 mL). The combined organic phase was washed with brine and water and dried over sodium sulfate. After the solvents were removed by rotary evaporation, the residue was purified by silica gel chromatography using DCM/hexane (1:9) as eluent, affording **12** as a yellow solid (740 mg, 96%). ^{1}H NMR (500 MHz, methylene chloride-*d*$_2$): *δ* 8.04 (s, 2H), 7.93 (t, *J* = 8.4 Hz, 4H), 7.81 (d, *J* = 9.0 Hz, 2H), 7.64 (q, *J* = 7.9 Hz, 6H), 7.30 – 7.22 (m, 8H), 7.19 (d, *J* = 6.8 Hz, 4H), 7.06 (d, *J* = 8.5 Hz, 2H), 6.75 (d, *J* = 64.2 Hz, 18H), 1.71 (s, 8H), 1.56 (s, 2H), 1.54 (s, 9H), 1.35 (s, 12H), 1.32 (s, 12H), 1.27 (s, 6H), 1.11 (s, 18H), 1.07 (s, 18H), 0.90 (s, 10H), 0.67 (d, *J* = 11.8 Hz, 36H). ^{13}C NMR (126 MHz, methylene chloride-*d*$_2$): *δ* 148.67, 148.45, 148.35, 148.26, 142.32, 142.12, 141.47, 141.32, 140.60, 139.51, 137.52, 137.37, 136.62, 132.45, 131.84, 131.58, 131.19, 130.67, 130.11, 129.87, 129.76, 129.25, 129.18, 126.28, 126.17, 125.79, 124.85, 123.89, 123.21, 123.08, 117.90 , 100.00, 91.06, 57.36, 57.04, 39.24, 39.22, 38.55, 38.53, 35.45, 34.40, 34.38, 34.34, 32.91, 32.89, 32.67, 32.65, 32.32, 32.22, 32.08, 32.06, 32.04, 31.73, 31.70, 31.37, 31.35, 31.29, 31.27. HRMS analysis (APCI): calcd for $C_{146}H_{175}$ $[M+H]^+$: 1928.3688; found: 1928.3677 (error: 0.6 ppm).

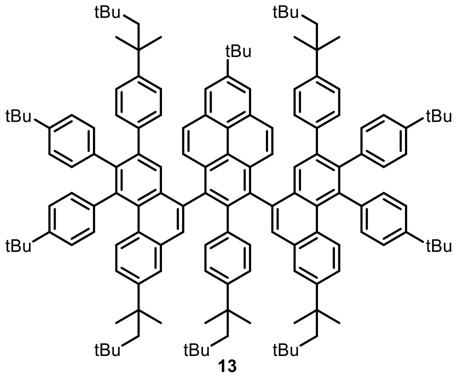

13

Compound **12** (190 mg, 0.1 mmol) and indium(III) chloride (6.6 mg, 0.03 mmol) were dissolved in mesitylene (20 mL) under nitrogen atmosphere. The mixture was heated overnight at 150 °C. After cooling to room temperature, the solvent was removed under vacuum. The mixture was extracted with DCM (3×30 mL) and sodium carbonate solution (30 mL). The combined organic layer was dried over sodium sulfate. The solvent was removed under vacuum and the residue was purified by column chromatography (silica gel, hexanes/DCM = 9/1) to afford compound **13** as an orange color solid (171 mg, 90%). ^{1}H NMR (500 MHz, methylene chloride-*d*$_2$): *δ* 8.23 (s, 2H), 7.97 (d, *J* = 9.3 Hz, 2H), 7.89 – 7.82 (m, 3H), 7.78 – 7.68 (m, 3H), 7.63 (s, 1H), 7.59 (s, 1H), 7.41 – 7.35 (m, 2H), 7.26 (d, *J* = 7.9 Hz, 2H), 7.14 (t, *J* = 7.5 Hz, 3H), 7.10 – 6.99 (m, 7H), 6.82 (dd, *J* = 22.4, 10.7 Hz, 11H), 6.71 (dd, *J* = 8.1, 4.2 Hz, 3H), 6.66 – 6.60 (m, 2H), 6.55 (t, *J* = 9.0 Hz, 2H), 1.82 – 1.72 (m, 4H), 1.58 (s, 12H), 1.45 – 1.35 (m, 14H), 1.31 (d, *J* = 2.1 Hz, 18H), 1.24 (s, 5H), 1.18 – 1.12 (m, 23H), 1.11 – 0.96 (m, 12H), 0.69 (d, *J* = 10.6 Hz, 18H), 0.56 (s, 10H), 0.49 (s, 6H), 0.13 (d, *J* = 24.2 Hz, 8H). ^{13}C NMR (126 MHz, methylene chloride-*d*$_2$): *δ* 149.58, 149.52, 148.02, 147.87, 147.79, 147.64, 147.60 , 147.44, 147.07, 140.99, 140.86, 140.55, 140.52, 139.68, 139.61, 139.44, 137.92, 137.84, 136.31, 136.05, 135.95, 133.29, 133.19, 133.16, 132.89, 131.60, 131.47, 131.14, 131.04, 130.62, 129.74, 129.71, 128.31, 127.91, 127.05, 126.73, 125.57,

125.39, 125.30, 125.13, 124.25, 124.07, 123.53, 122.85, 100.38, 67.42, 38.66, 38.64, 38.61, 38.38, 38.36, 38.28, 38.07, 38.04, 34.68, 34.35, 32.63, 32.52, 32.47, 32.01, 31.94, 31.88, 31.56, 31.53, 31.45, 31.40, 31.37. HRMS analysis (APCI): calcd. for $C_{146}H_{175}$ $[M+H]^+$: 1928.3688; found: 1928.3659 (error: 1.6 ppm).

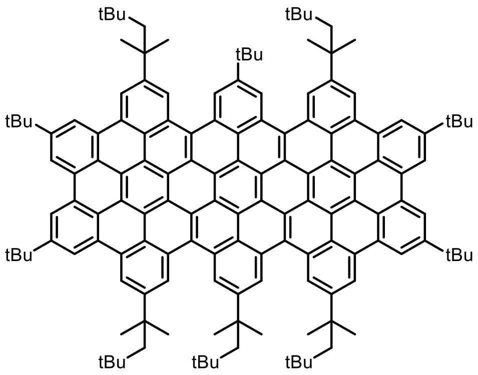

HBPO

A solution of compound **13** (30 mg, 0.015 mmol) and 2,3-dichloro-5,6-dicyano-1,4-benzoquinone (DDQ, 60 mg, 0.27 mmol) in dry degassed DCM (10 mL) was stirred at 0°C for 15 mins. Then trifluoromethane sulfonic acid (0.11 mL) was added in one portion. The mixture was stirred overnight at room temperature and subsequently quenched with Et_3N (1 mL). After 30 mins, the mixture was extracted with DCM (2×50 mL) and aqueous sodium bicarbonate (50 mL). The combined organic portion was dried with sodium sulfate. The solvent was removed under vacuum and the residue was washed with methanol (30 mL) to remove the remaining DDQ. After filtration, the precipitated solid was further washed with hexane to remove partially oxidized compounds. The precipitate was purified by column chromatography (silica gel, toluene) to afford the target compound **HBPO** as a magenta solid (14 mg, 46%). ^{1}H NMR (500 MHz, THF-d_8:CS_2=2:1, 298K): δ 10.32 (d, J = 13.3 Hz, 4H), 10.19 (d, J = 17.1 Hz, 4H), 9.70 (s, 4H), 9.58 (s, 4H), 9.51 (s, 4H), 2.44 (s, 8H), 2.21 (s, 6H), 2.14 (d, J = 11.6 Hz, 26H), 1.97 (s, 36H), 1.93 (s, 9H), 0.94 (s, 36H), 0.39 (s, 9H). ^{13}C NMR (126 MHz, THF-d_8:CS_2=2:1, 298K): δ 150.63, 149.36, 149.22, 149.05, 132.38, 132.09, 131.31, 130.37, 129.35, 129.09, 127.13, 126.43, 126.27, 126.19, 126.16, 125.93, 125.86, 125.40, 125.36, 123.05, 122.40, 122.25, 122.19, 122.15, 122.11, 121.33, 121.27, 120.57, 120.36, 58.60, 58.53, 58.37, 41.01, 40.99, 40.95, 36.88, 36.68, 33.77, 33.49, 33.47, 33.35, 33.04, 32.91, 32.89, 32.67, 32.24, 30.93. HRMS analysis (APCI): calcd for $C_{146}H_{151}$ $[M+H]^+$: 1904.1810; found: 1904.1802 (error: 0.4 ppm).

2. Fluorescence measurement

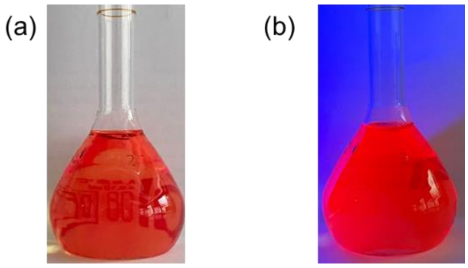

FigureS1. (a) Fluorescence of **HBPO** under excitation of indoor light and (b) under excitation at 365 nm.

3. Cyclic voltammetry of HBPO

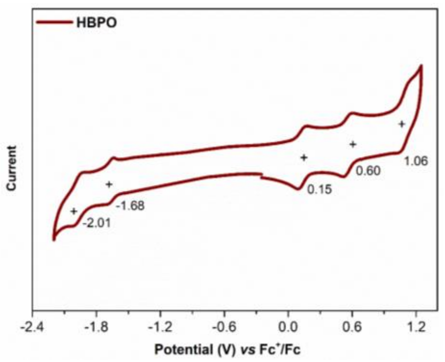

Figure S2. Cyclic voltammogram of **HBPO** in DCM containing 0.1 M *n*-Bu_4NPF_6 at room temperature.

Using the ferrocene/ferrocenium couple as an external reference, the electrochemical properties of **HBPO** were studied by cyclic voltammetry (CV). Three oxidation waves with half-wave potentials $E_{1/2}^{ox}$ at 0.15, 0.60, and 1.06 V and two reduction waves with half-wave potentials $E_{1/2}^{red}$ at -1.68 and -2.01 V were observed. The HOMO and LUMO energy levels were estimated from the onset of the first oxidation/reduction wave potential to be 4.92 eV and 3.17 eV, respectively, consistent with the molecular orbital energy levels based on DFT calculations (4.33 and 2.23 eV). Therefore, the electrochemical energy gap was determined to be 1.75 eV.

4. DFT calculations

Theoretical calculations were performed with the Gaussian09 rev. D program suite.[2] All calculations were carried out using the density functional theory (DFT) method with Becke's three-parameter hybrid exchange functionals and the Lee-Yang-Parr correlation functional (B3LYP) employing the 6-31G(d,p) basis set for all atoms.[5] The geometry of **HBPO** was optimized under a Pople basis set 6-31G (d,p).[6]

NICS values were calculated using the standard GIAO procedure.[7] ACID plot was calculated by using the method developed by Herges.[8]

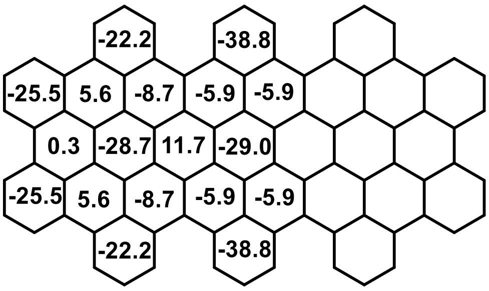

Figure S3. NICS(1)$_{zz}$ values calculated based on the butterfly conformations of **HBPO**.

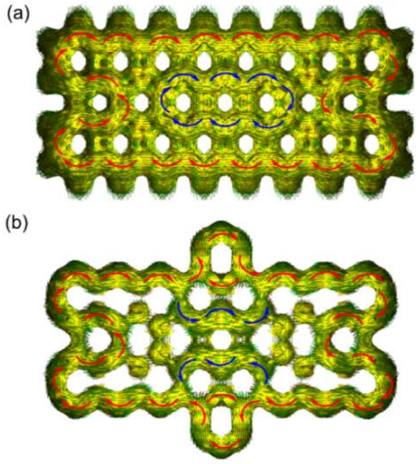

Figure S4. Calculated ACID plot (contribution from π electrons only) of (a) *peri*-octacene, and (b) dibenzo-*peri*-octacene.

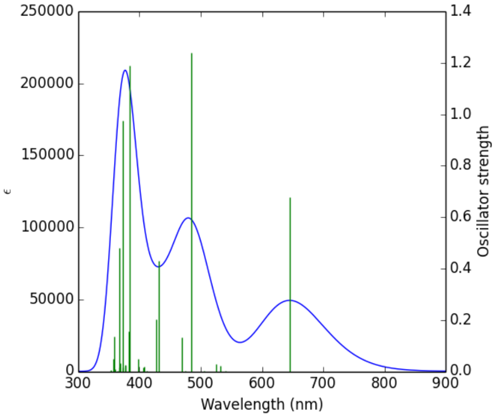

Figure S5. Calculated UV-vis absorption spectrum of **HBPO** obtained by TD-DFT calculation at B3LYP/6-31G(d,p) level of theory (isovalue=0.025).

Table S1. Table Selected calculated wavelength, oscillator strength, and compositions of major transitions of **HBPO** from TD-DFT (B3LYP/6-31G (d,p)).

Wavelength (nm)	Osc. Strength	Major contributions
645.6	0.6785	H→L (98%)
485.1	1.2405	H-2→L (34%), H→L+2 (53%), H→L+3 (11%)
468.9	0.1326	H-4→L (16%), H→L+4 (73%)
430.9	0.4283	H-4→L (75%), H→L+4 (18%)
384.0	1.1889	H-3→L+1 (39%), H-1→L+2 (26%), H-1→L+3 (27%)
383.0	0.1563	H-6→LUMO (11%), H-2→L+1 (30%), H-1→L+2 (23%), H-1→L+3 (28%)

The second maximum absorption band at 456 nm is attributed to a combination of H-2→L, H→L+2, and H→L+3 electronic transition. The shortest-wavelength absorption band appears at 362 nm because of the multiple components of electronic transition, including H-3→L+1, H-1→L+2, and H-1→L+3.

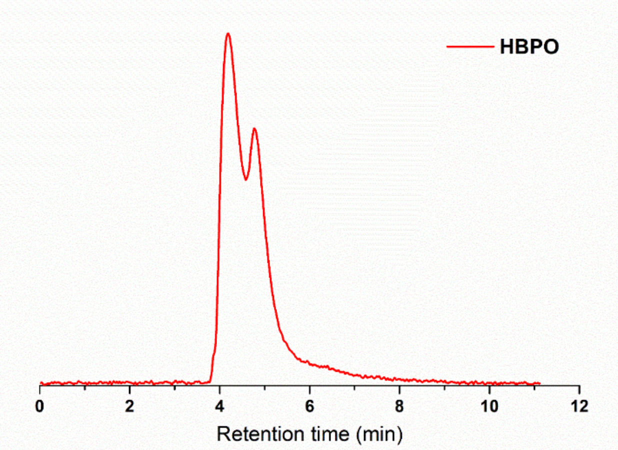

Figure S6. Chiral HPLC trace during the separation of **HBPO** monitored at 602 nm. A mixture of THF/*n*-hexane = 1:9 was used as the eluent with a flow rate of 1 mL/min. Enantiomeric resolution of **HBPO** with chiral HPLC exhibited two distinct bands, indicating a mixture of conformers.

DFT calculations were performed to evaluate the isomerization process of **HBPO**. As demonstrated in Figure S7, there is one plausible pathway, involving two transition states (TSs) and one metastable helical conformation. The simplified **HBPO** without branched alkyl substituents was also optimized to calculate the isomerization barriers for comparison.

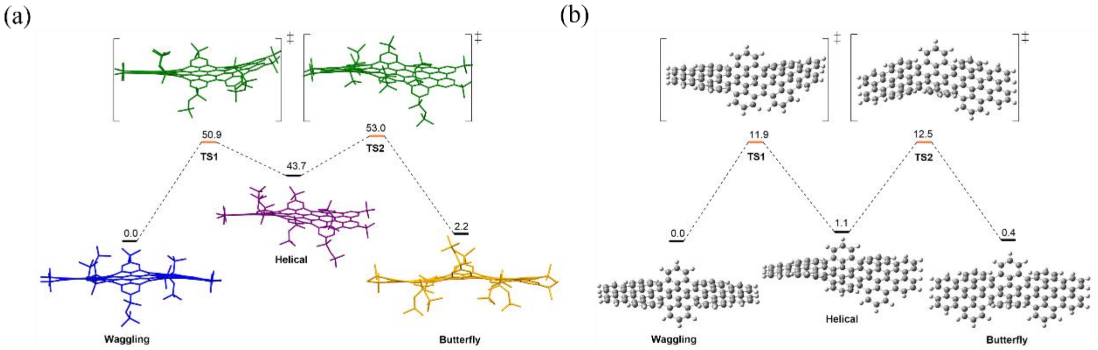

Figure S7. Isomerization processes of **HBPO** from the waggling conformation to the butterfly conformation based on its crystal structure. The relative Gibbs free energy Δ*G* (kcal/mol) was calculated at the B3LYP/6-31G(d,p) level. TS: transition state. (a) **HBPO**; (b) simplified **HBPO** without branched alkyl chains.

5. Transient Absorption Spectroscopy and Amplified Stimulated Emission Characterization of HBPO

HBPO solutions and films for TA spectroscopy and ASE measurements were prepared as follows. **HBPO** solutions were obtained by diluting **HBPO** in anhydrous chloroform at a concentration displaying high fluorescence quantum yield (Optical density ~ 0.1 at 602 nm). For this purpose, 1 mg of **HBPO** was dissolved in 5 mL of anhydrous chloroform and diluted several times. Before any dilution or measurement, a mild sonication was applied to the sample. All steps of sample preparation were performed under an inert atmosphere. **HBPO/**PS blend films were prepared with 1.24, 2,43, and 5 wt% **HBPO** contents. Accordingly, 1 mg of **HBPO** was dispersed in 2 mL of chloroform and sonicated mildly. In parallel, a solution containing 60 mg of PS in 1 mL of anhydrous chloroform was prepared and stirred overnight. Both master solutions were subsequently mixed to obtain the desired **HBPO** wt% content. Finally, **HBPO**/PS solutions were spin-coated on top of Spectrosil B substrates (v = 1000 rpm, t = 1 s). The thicknesses of the final films were around 500 nm.

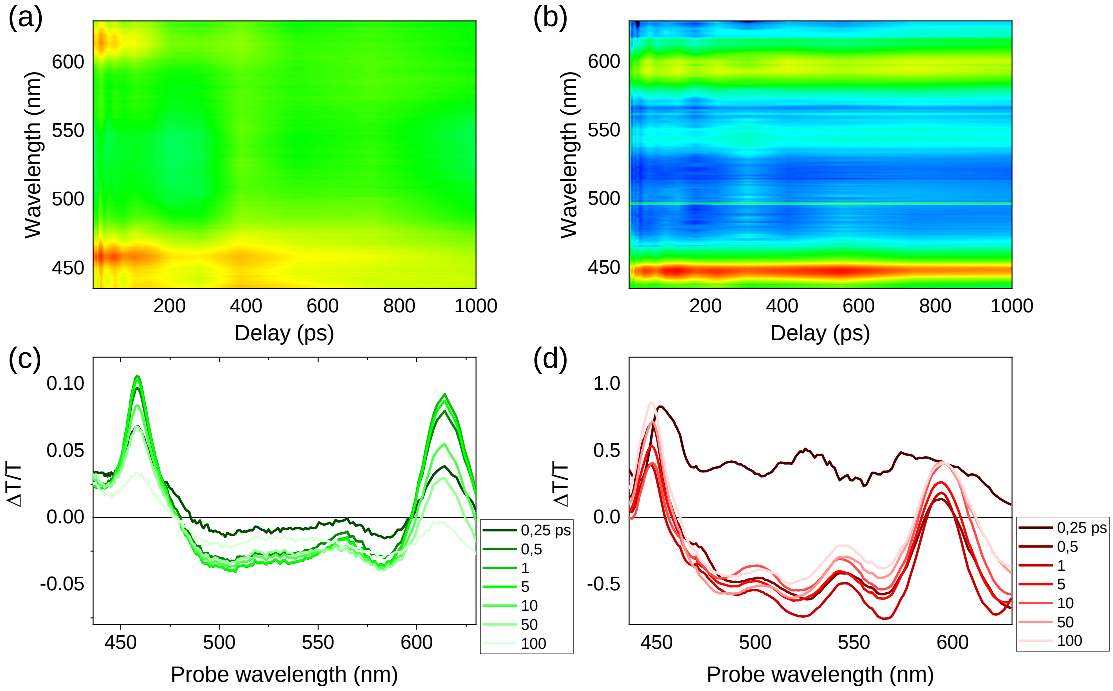

Figure S8. Transient absorption contour plot of (a) **HBPO/PS** blend film (b) and the **HBPO**in solution. ΔT/T spectra at different delays of (c) **HBPO/PS blend film** and (d) the **HBPO**in solution. Measurements were conducted with 387 nm pump wavelength.

The TA spectra of the **HBPO** solution are dominated by a broad ESA band (negative DT/T). A 613 nm bump appears superimposed with ESA and it is likely assigned to SE. It cannot be ascribed to GSB since it has no resemblance in the ground state absorption. The SE assignment is further reinforced by the bump spectral location being resonant with the 0-0 vibronic peak of the fluorescence spectrum. Differing from the solution, the 613 nm SE band in the **HBPO**/PS blend dominates over ESA.

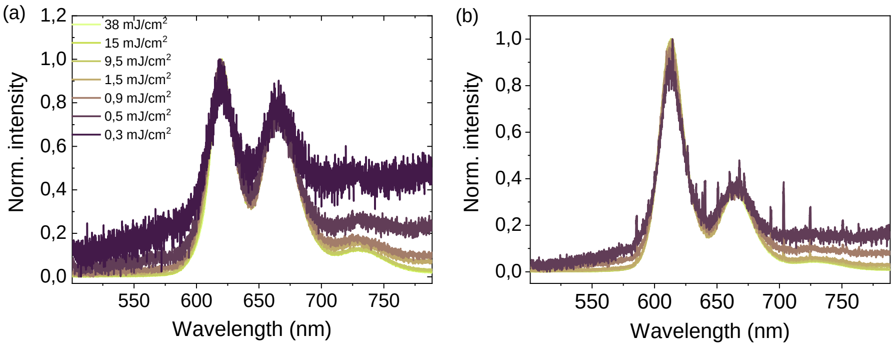

Figure S9. Fluorescence measurements in **HPBO** varying the pump fluence between 0.3 mJ/cm^2 and 38 mJ/cm^2 in (a) a 1 mg/mL solution, (b) a 0.04 mg/mL solution.

HBPO solutions did not exhibit ASE (Figure S8) whereas a clear linewidth narrowing was observed in a 1.24 wt% blend film of **HBPO** in PS (Figure S9), in line with the observation of stimulated emission in

transient absorption measurements. Figure S10 depicts ASE measurements performed in the blend while changing continuously the irradiation position. Upon pumping at 355 nm, a linewidth narrowing was manifested at pump fluences around 36 mJ/cm^2, close to the degradation onset of the sample. Indeed, ASE started to vanish when the sample was irradiated for more than five seconds. Tuning the pump wavelength from 355 to 456 nm led instead to stable and efficient ASE throughout the entire **HBPO**/PS film, (Figure S11).

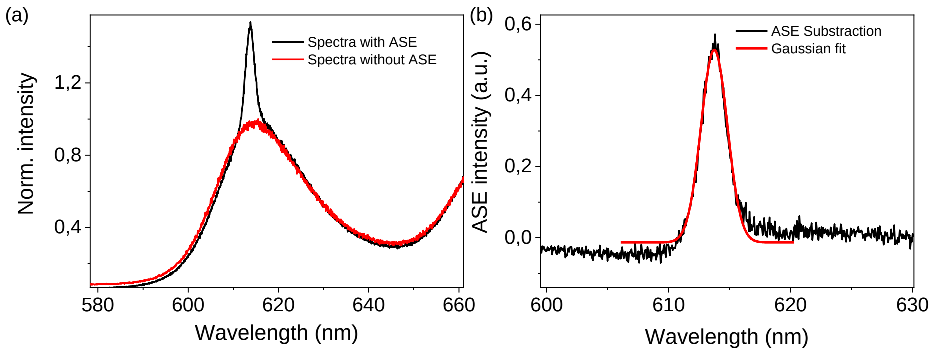

Figure S10. (a) Emission spectra of a **HPBO/**PS blend film below (red) and above (black) the ASE threshold. (b) ASE signal substracted from the fluorescence background, obtaining a full-width half maximum of 2.55 nm. The photoexcitation wavelength was 355 nm.

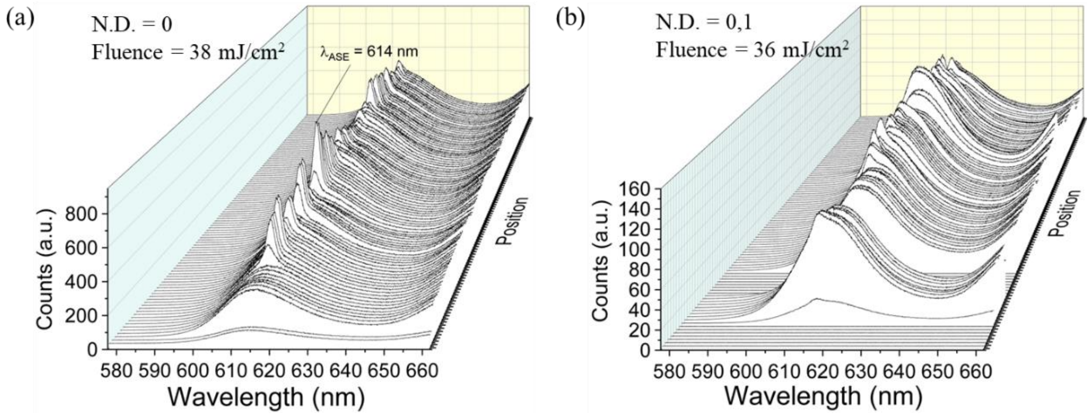

Figure S11. Scanned emission spectra across the blend film photoexcited at 355 nm. The ASE peak at 614 nm is visible in some film positions. The pump fluence was (a) above and (b) below the 36 mJ/cm^2 ASE threshold.

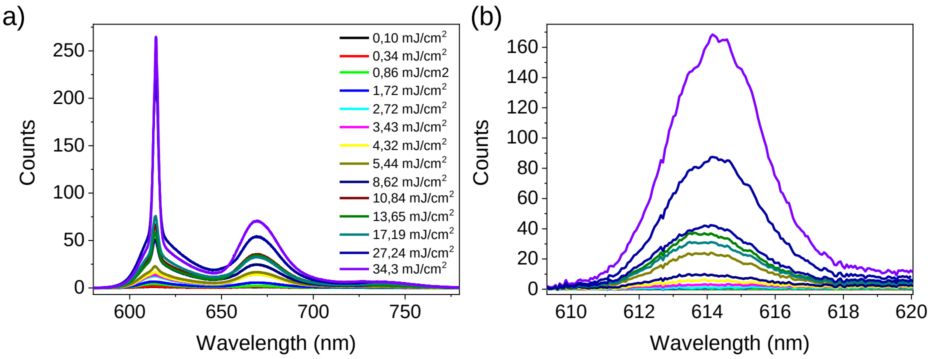

Figure S12. (a) Emission spectra photoexcited at 456 nm with different pump fluences. (b) Expanded spectra around the ASE spectral region. The same legend applies for (a) and (b).

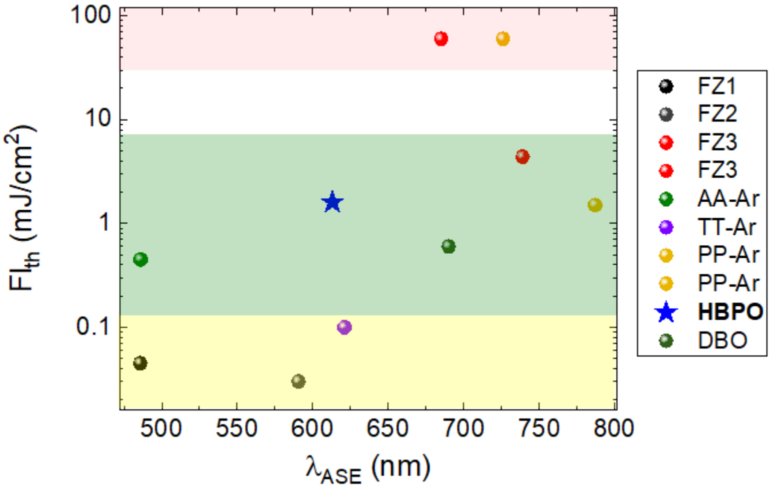

Figure S13. Thresholds and ASE wavelengths of **HBPO** by comparing with the literature. Datapoints have been obtained from the reported references.[9-11]

6. X-ray Crystallographic data

The single crystals of compound **HBPO** suitable for X-ray analysis were obtained by slow diffusion of methanol into the toluene solution of **HBPO**. The X-ray diffraction data were collected at 120 K on a STOE IPDS 2T diffractometer by using graphite monochromated Cu-Kα radiation.
Cambridge Crystallographic Data Centre and the data can be obtained free of charge via www.ccdc.cam.ac.uk/structures. Crystallographic data with CCDC number 2042061 and 2141365.

Table S2. Crystal data and structure refinement for **HBPO** grown in toluene/methanol.

Empirical formula	$C_{146}H_{150}$
moiety formula	$C_{146}H_{150}$ + [solvent]

Formula weight	1904.65 g/mol	
Temperature	120(2) K	
Wavelength	1.54178Å, CuK α	
Diffractometer	STOE IPDS 2T	
Crystal system	triclinic	
Space group	P -1, (2)	
Unit cell dimensions	a = 23.1399(10) Å	α = 78.775(3)°
	b = 23.7733(11) Å	β = 71.015(3)°
	c = 26.3841(19) Å	γ = 81.423(4)°
Volume	13404.4(11) $Å^3$	
Number of reflections	70579	
and range used for lattice parameters	2.26° <= θ <= 64.70°	
Z	4	
Density (calculated)	0.944 Mg/m^3	
Absorption coefficient	0.395 mm^{-1}	
Absorption correction	None	
F(000)	4104	
Crystal size, colour and form	0.040 x 0.190 x 0.240 mm^3, brown sheet	
Theta range for data collection	2.259 to 68.073°.	
Index ranges	-26<=h<=25, -27<=k<=27, -31<=l<=30	
Reflections collected	120006	
Independent reflections	45893 [R(int) = 0.0409]	
observed [I>2sigma(I)]	20398	
Completeness to theta = 67.7°	94.4 %	
Refinement method	Full-matrix least-squares on F^2	
Data /restraints / parameters	45893 / 1884 / 2030	
Goodness-of-fit on F^2	1.883	
Final R indices [I>2sigma(I)]	R1 = 0.2072, wR2 = 0.5079	
R indices (all data)	R1 = 0.2833, wR2 = 0.5807	
Largest diff. peak and hole	0.882 and -0.531 $eÅ^{-3}$	
Remark	crystals contain at least six molecules of CH_3OH which could not be refined – Squeeze was used, Two slightly different	

	independent molecules, **only a proof of synthesis**.

Table S3. Crystal data and structure refinement for **HBPO** grown in mesitylene/methanol.

Empirical formula	$C_{173}H_{186}$	
moiety formula	$C_{146}H_{150}$ + 3[C_9H_{12}]	
Formula weight	2265.21 g/mol	
Temperature	120(2) K	
Wavelength	0.71073Å, MoK α	
Diffractometer	STOE IPDS 2T	
Crystal system	Triclinic	
Space group	P -1, (2)	
Unit cell dimensions	a = 10.7542(6) Å	α = 89.158(5)°
	b = 14.8759(8) Å	β = 84.844(5)°
	c = 20.7535(14) Å	γ = 79.496(5)°
Volume	3251.3(3) Å^3	
Number of reflections	27191	
and range used for lattice parameters	2.17° <= θ <= 28.40°	
Z	1	
Density (calculated)	1.157 Mg/m^3	
Absorption coefficient	0.065 mm^{-1}	
Absorption correction	Integration	
F(000)	1224	
Crystal size, colour and form	0.070 x 0.150 x 0.490 mm^3, red plate	
Theta range for data collection	2.415 to 28.176°.	
Index ranges	-14<=h<=14, -17<=k<=19, -27<=l<=27	
Reflections collected	46312	
Independent reflections	15584 [R(int) = 0.0791]	
observed [I>2sigma(I)]	7276	
Completeness to theta = 67.7°	99.9 %	
Refinement method	Full-matrix least-squares on F^2	
Data /restraints / parameters	15584 / 223 / 961	
Goodness-of-fit on F^2	1.127	
Final R indices [I>2sigma(I)]	R1 = 0.1344, wR2 = 0.2373	
R indices (all data)	R1 = 0.2549, wR2 = 0.3047	
Largest diff. peak and hole	0.346 and -0.358 eÅ^{-3}	
Remark	molecule has pseudo-Ci symmetry, t-butyl and tert-octyl groups are disordered, one mesitylene is half occupied at centre of inversion	

7. NMR and Mass spectra

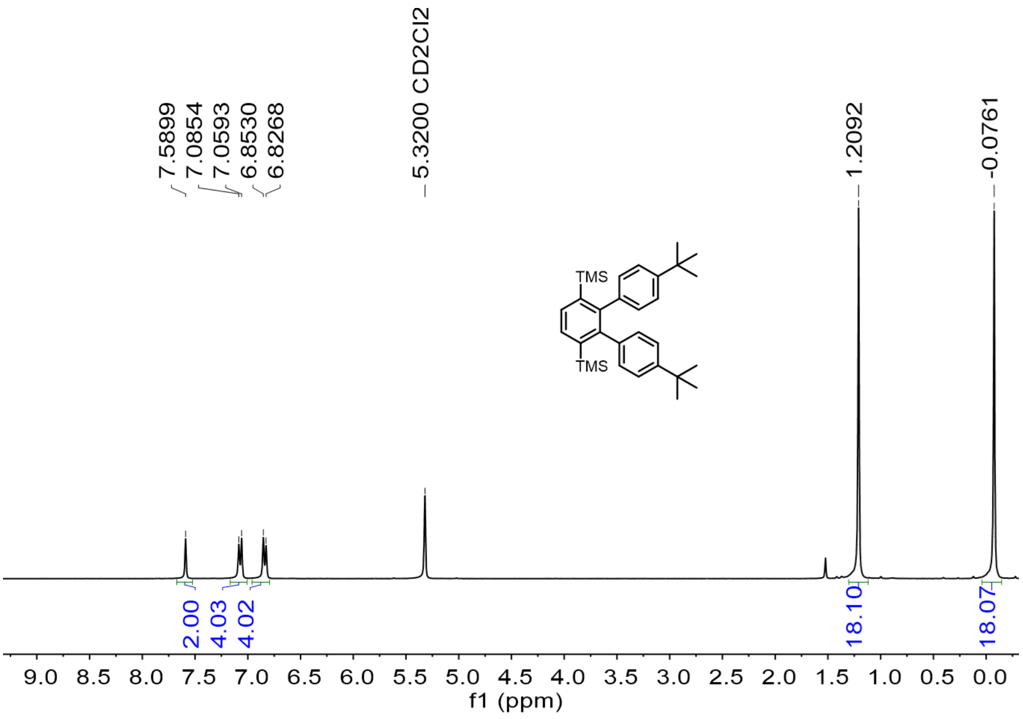

Figure S14. ^{1}H NMR spectrum of compound **2** (300 MHz, methylene chloride-d_2).

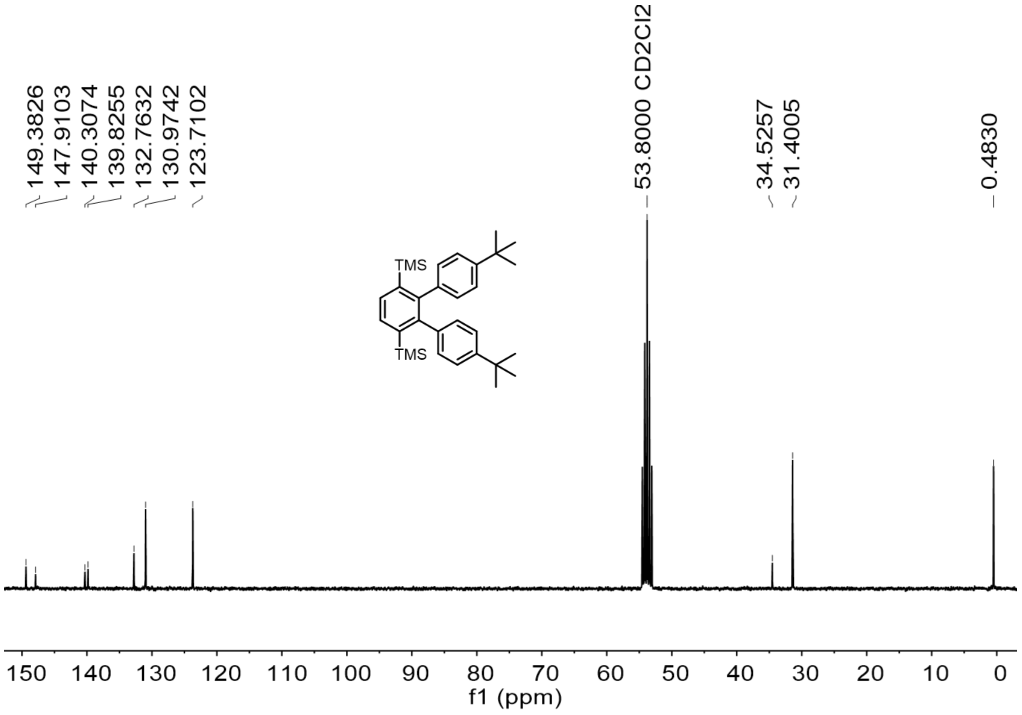

Figure S15. ^{13}C NMR spectrum of compound **2** (75 MHz, methylene chloride-d_2).

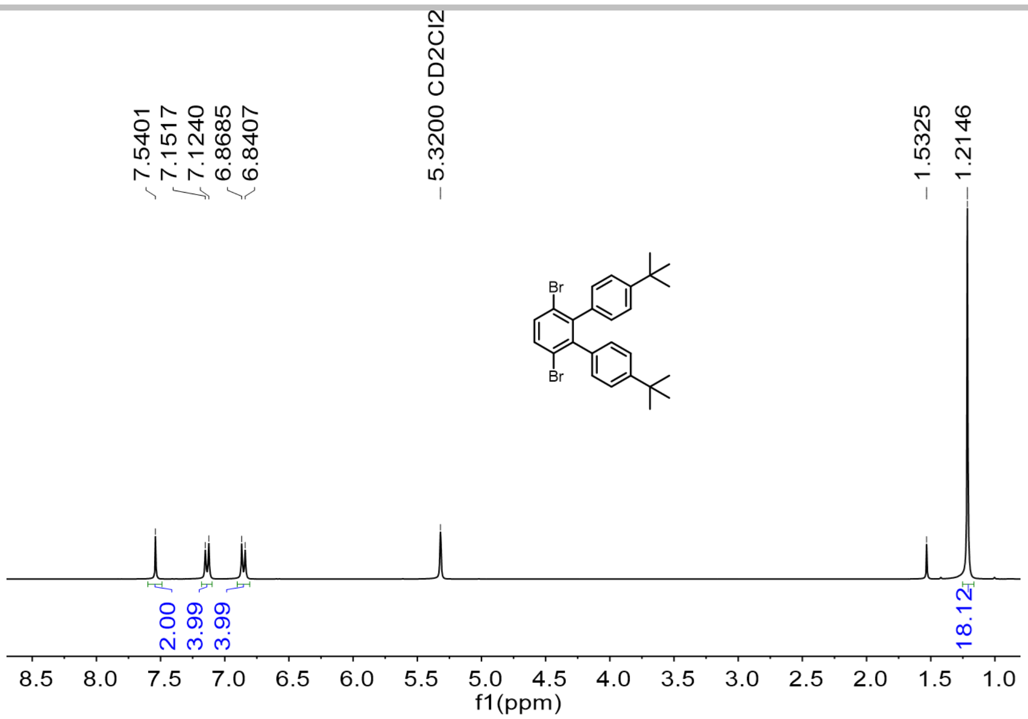

Figure S16. ^{1}H NMR spectrum of compound **3** (300 MHz, methylene chloride-d_2).

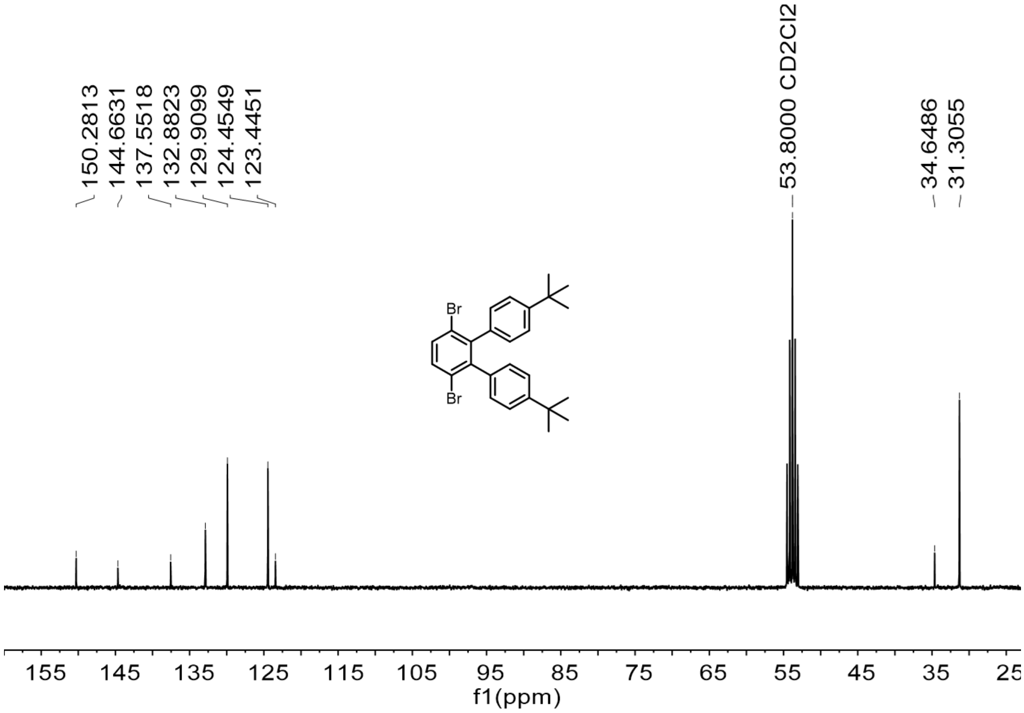

Figure S17. ^{13}C NMR spectrum of compound **3** (75 MHz, methylene chloride-d_2).

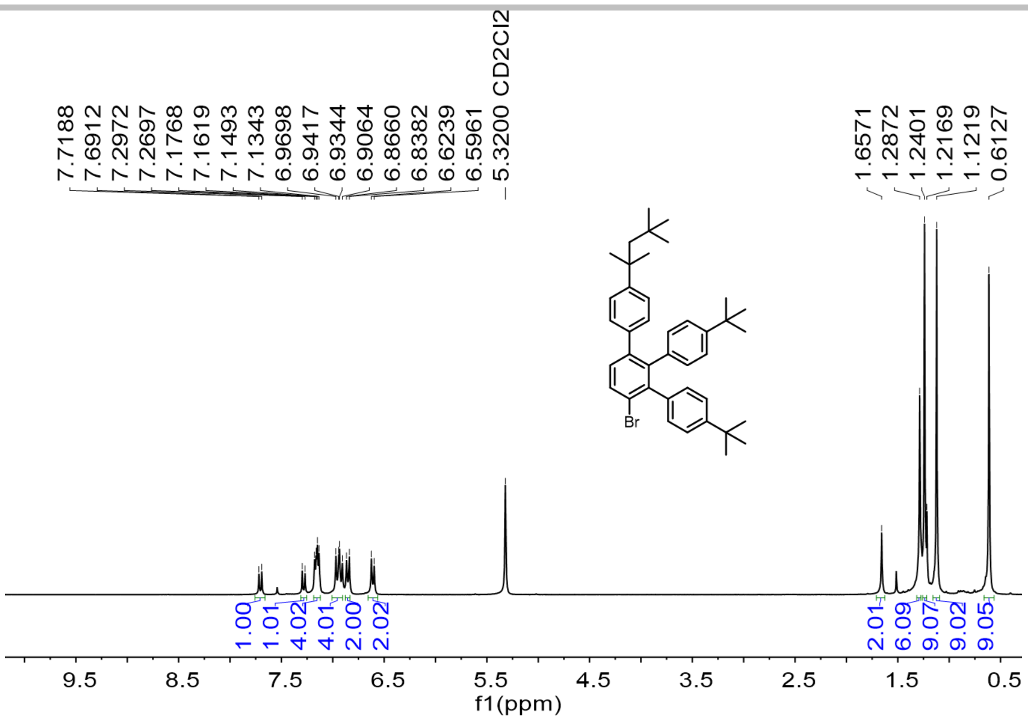

Figure S18. ^{1}H NMR spectrum of compound **4** (300 MHz, methylene chloride-d_2).

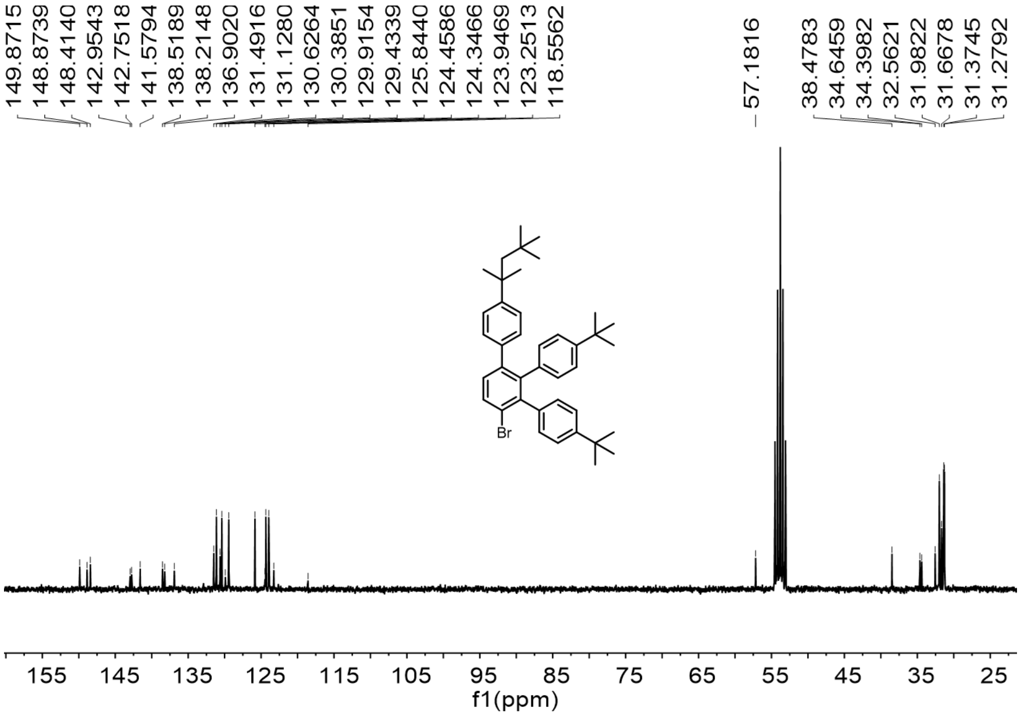

Figure S19. ^{13}C NMR spectrum of compound **4** (75 MHz, methylene chloride-d_2).

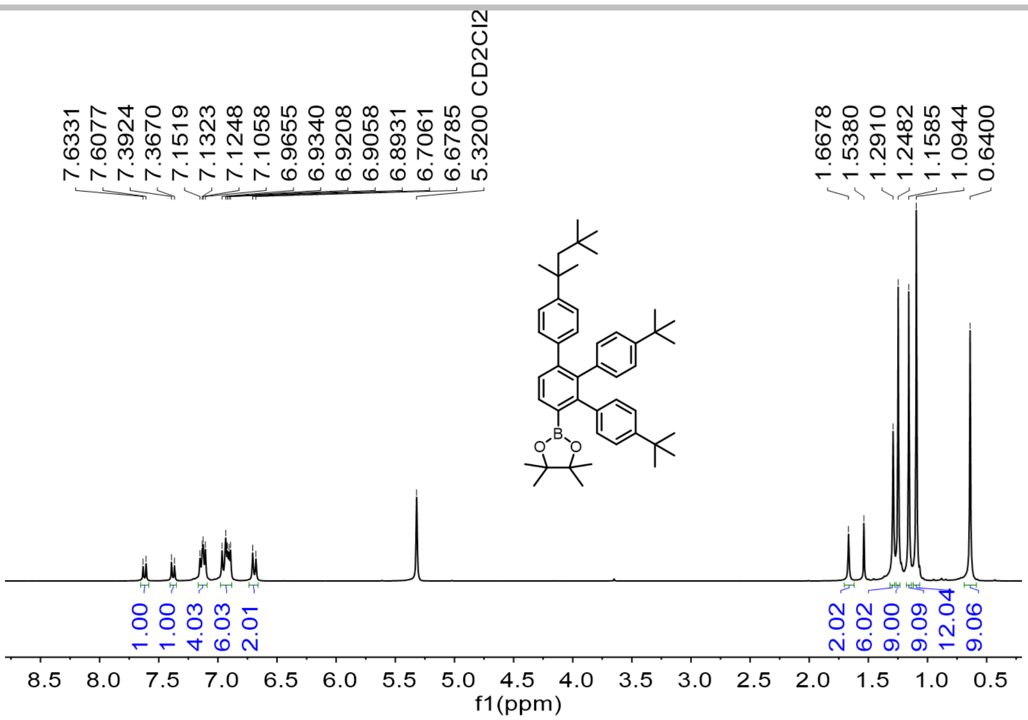

Figure S20. ^{1}H NMR spectrum of compound **5** (300 MHz, methylene chloride-d_2).

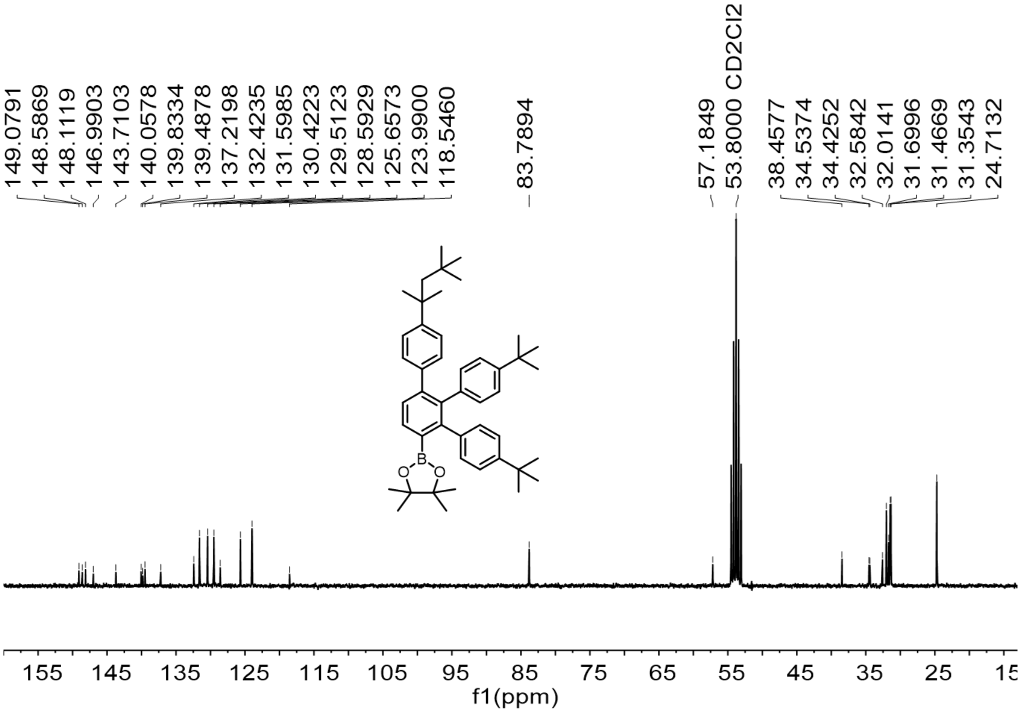

Figure S21. ^{13}C NMR spectrum of compound **5** (75 MHz, methylene chloride-d_2).

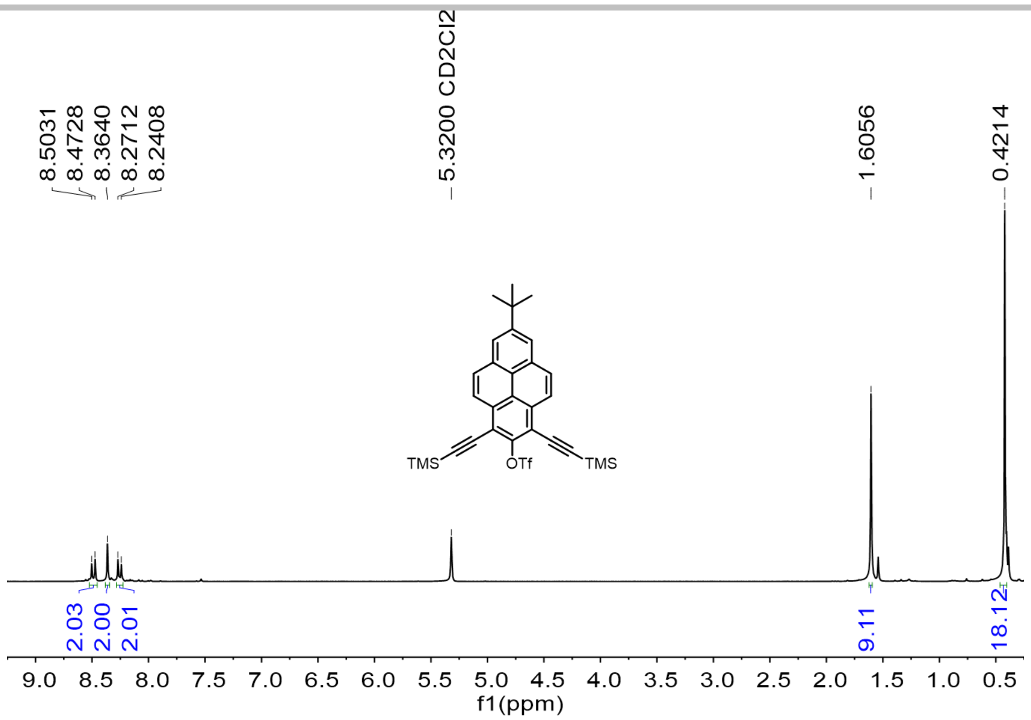

Figure S22. ^{1}H NMR spectrum of compound **7** (300 MHz, methylene chloride-*d*$_2$).

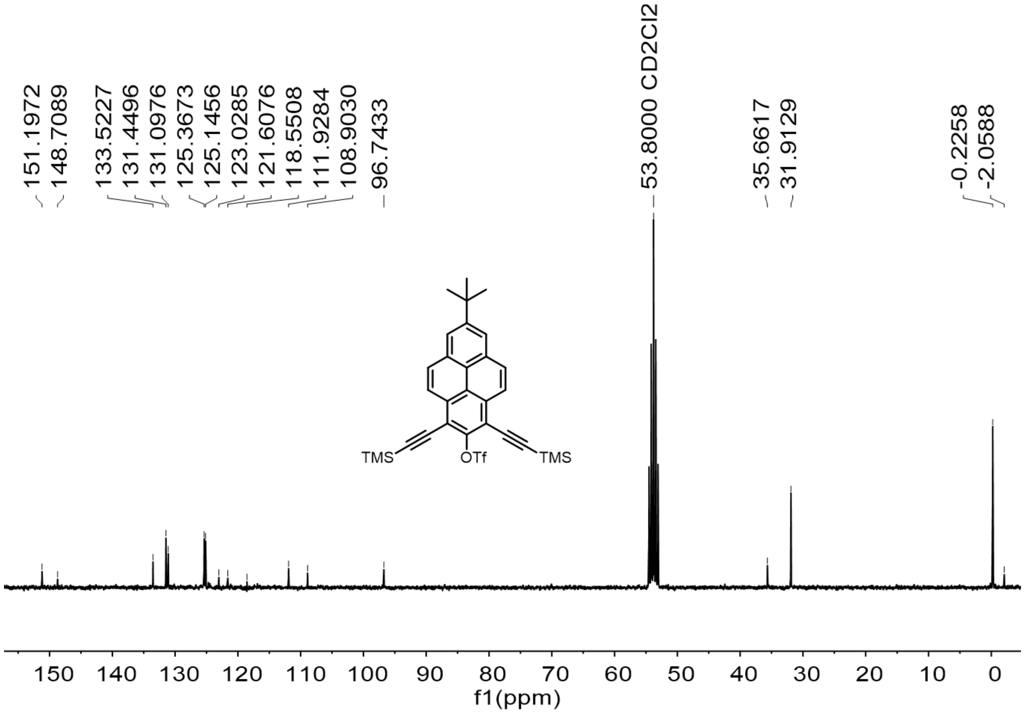

Figure S23. ^{13}C NMR spectrum of compound **7** (75 MHz, methylene chloride-*d*$_2$).

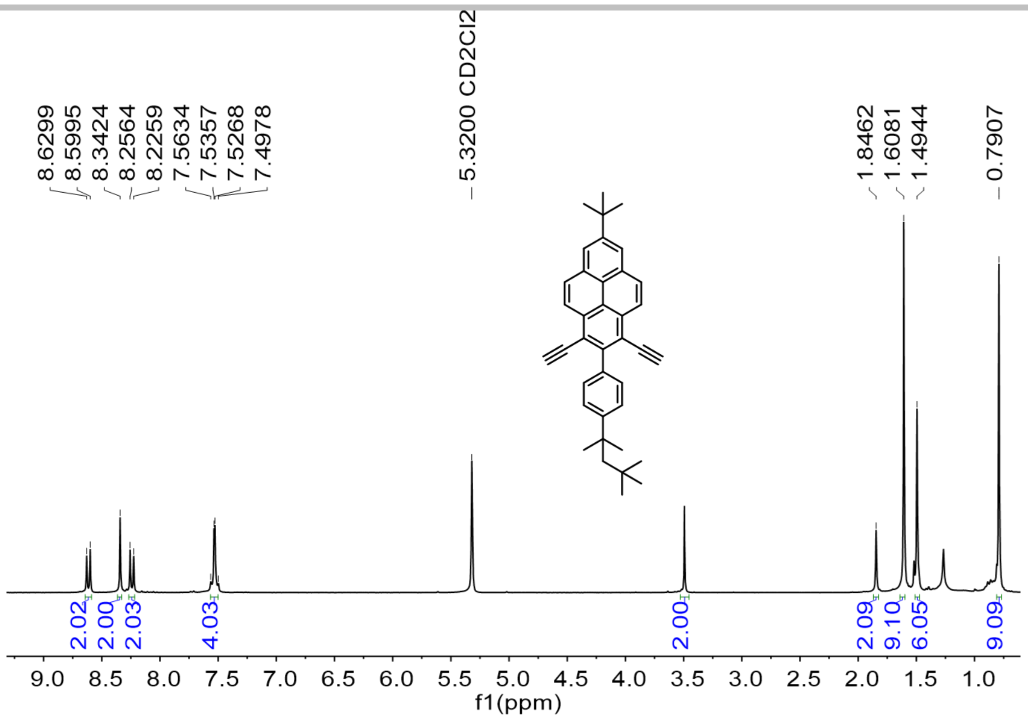

Figure S24. ^{1}H NMR spectrum of compound **9** (300 MHz, methylene chloride-d_2).

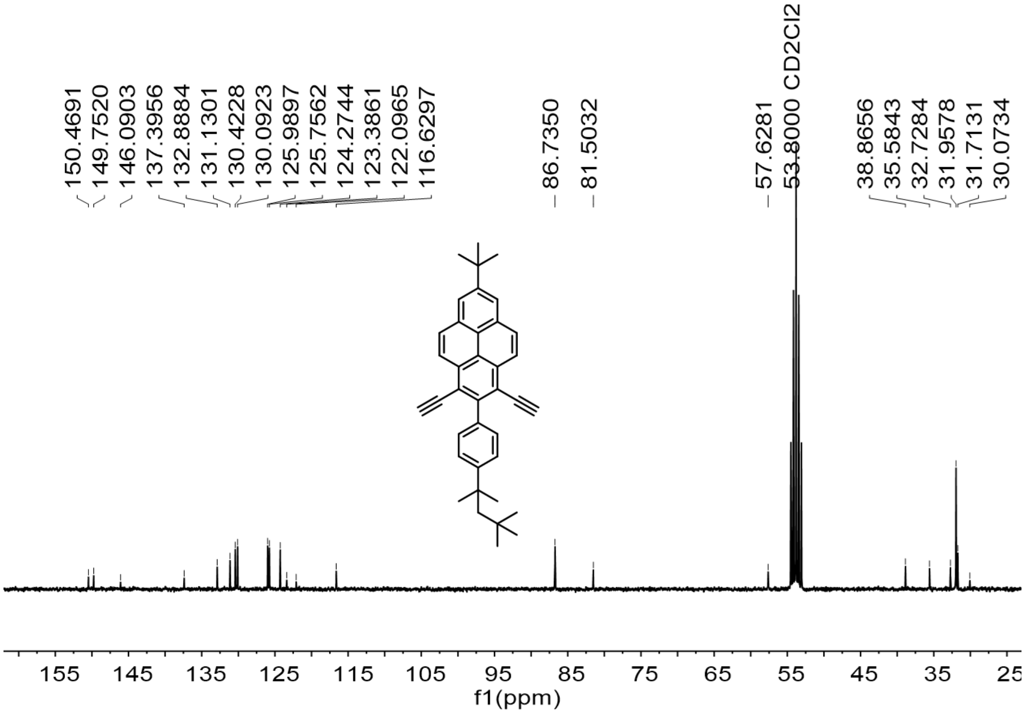

Figure S25. ^{13}C NMR spectrum of compound **9** (75 MHz, methylene chloride-d_2).

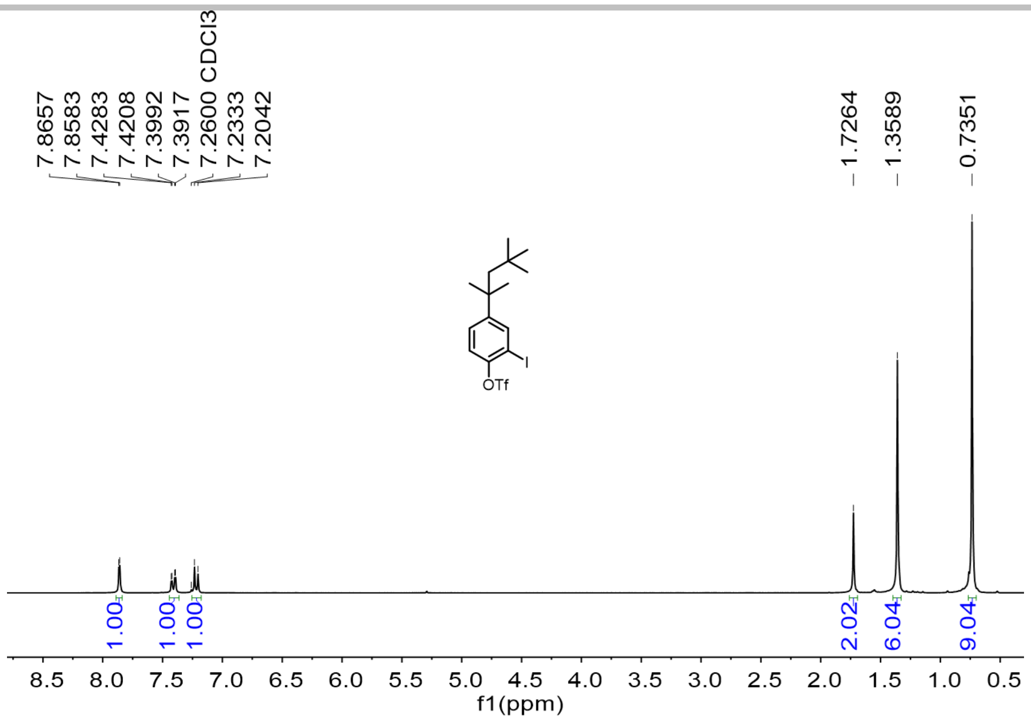

Figure S26. ^{1}H NMR spectrum of compound **10** (300 MHz, chloroform-*d*).

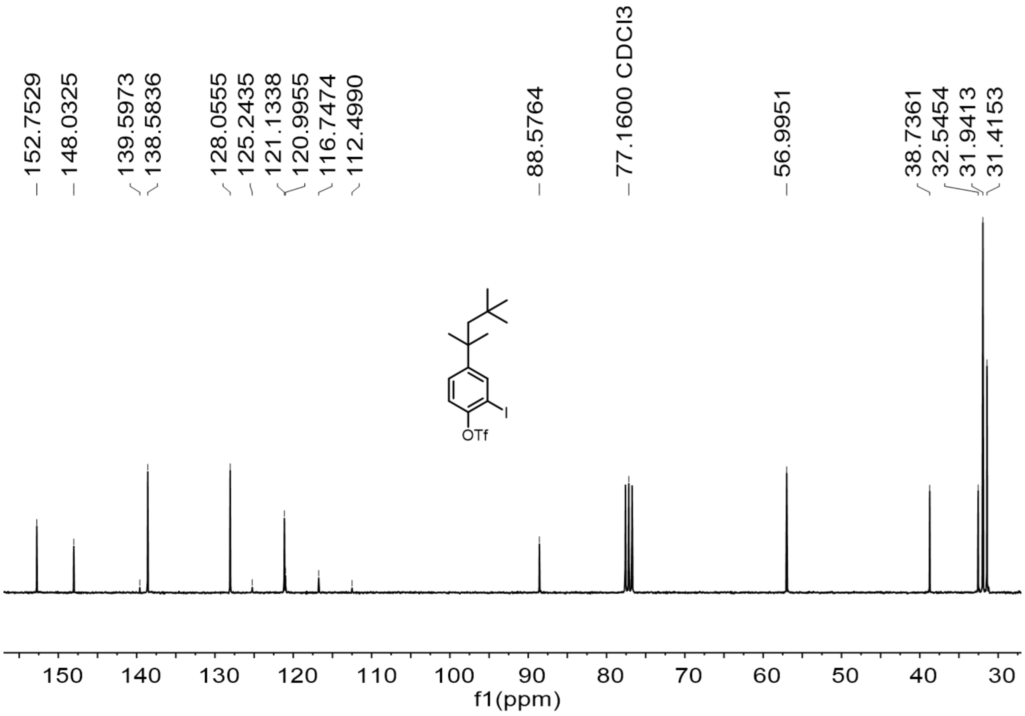

Figure S27. ^{13}C NMR spectrum of compound **10** (75 MHz, chloroform-*d*).

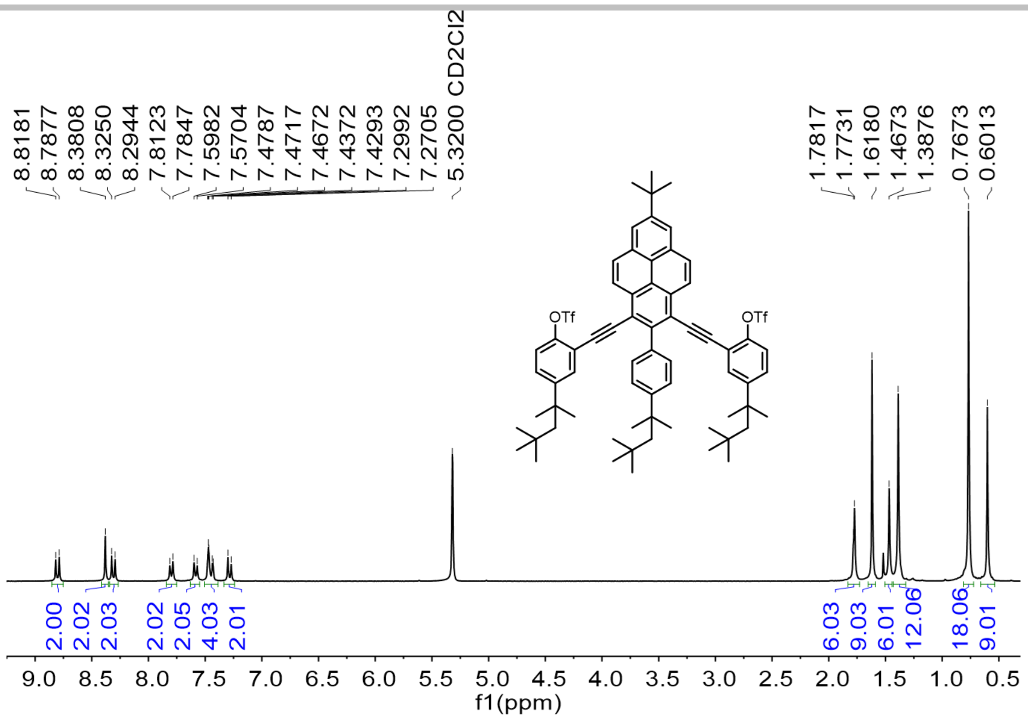

Figure S28. ^{1}H NMR spectrum of compound **11** (300 MHz, methylene chloride-d_2).

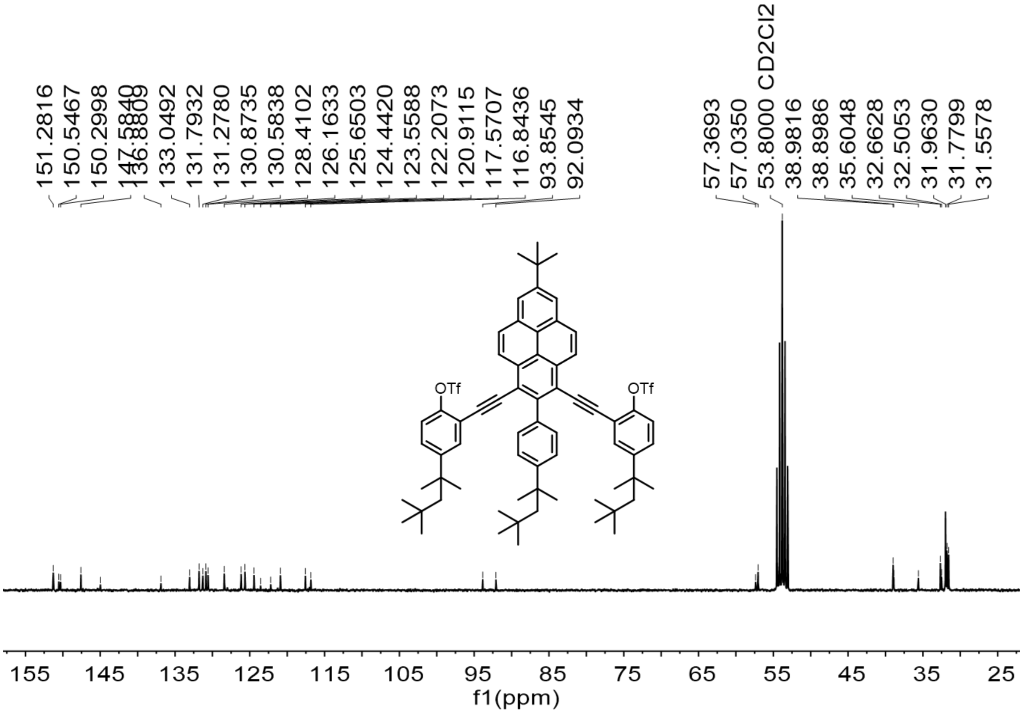

Figure S29. ^{13}C NMR spectrum of compound **11** (75 MHz, methylene chloride-d_2).

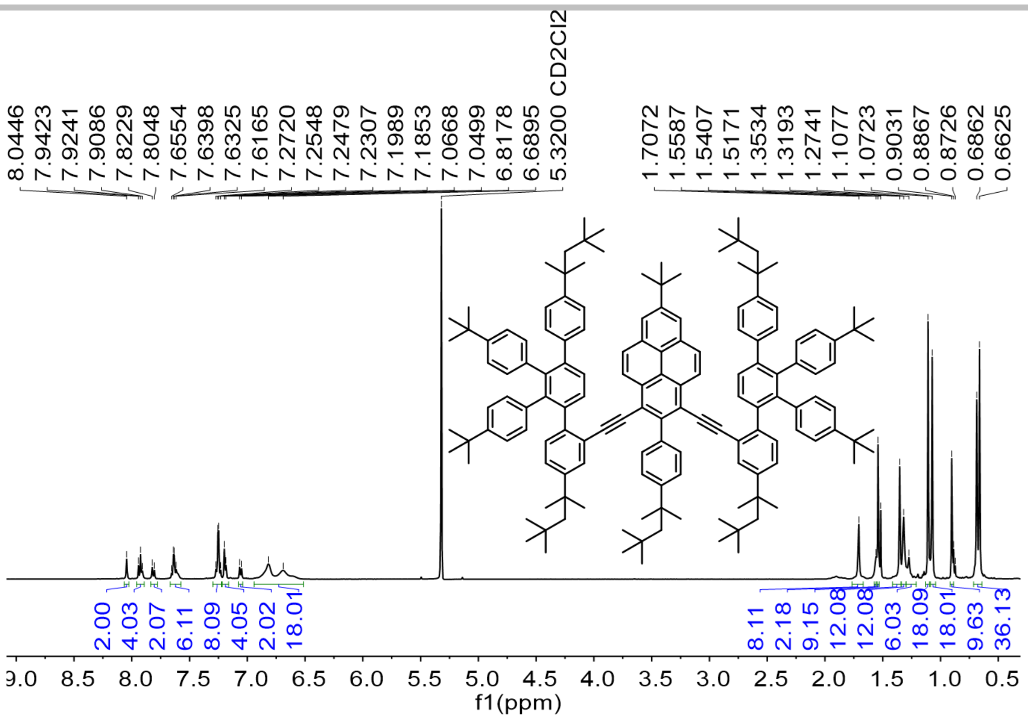

Figure S30. ^{1}H NMR spectrum of compound **12** (500 MHz, methylene chloride-d_2).

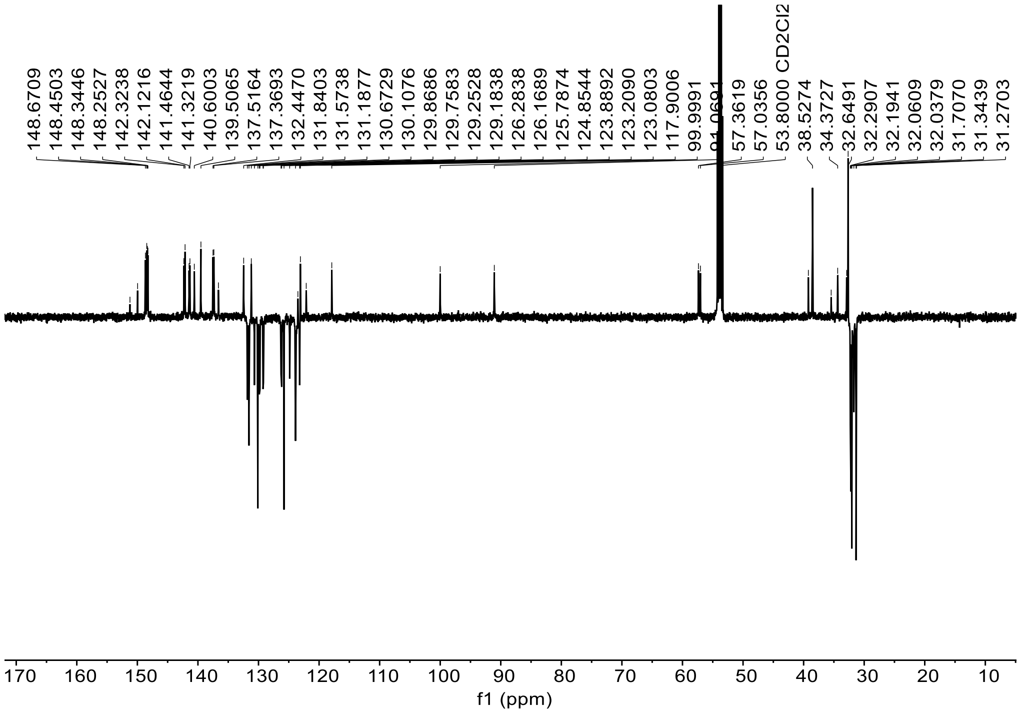

Figure S31. Spin-echo ^{13}C NMR spectrum of compound **12** (126 MHz, methylene chloride-d_2).

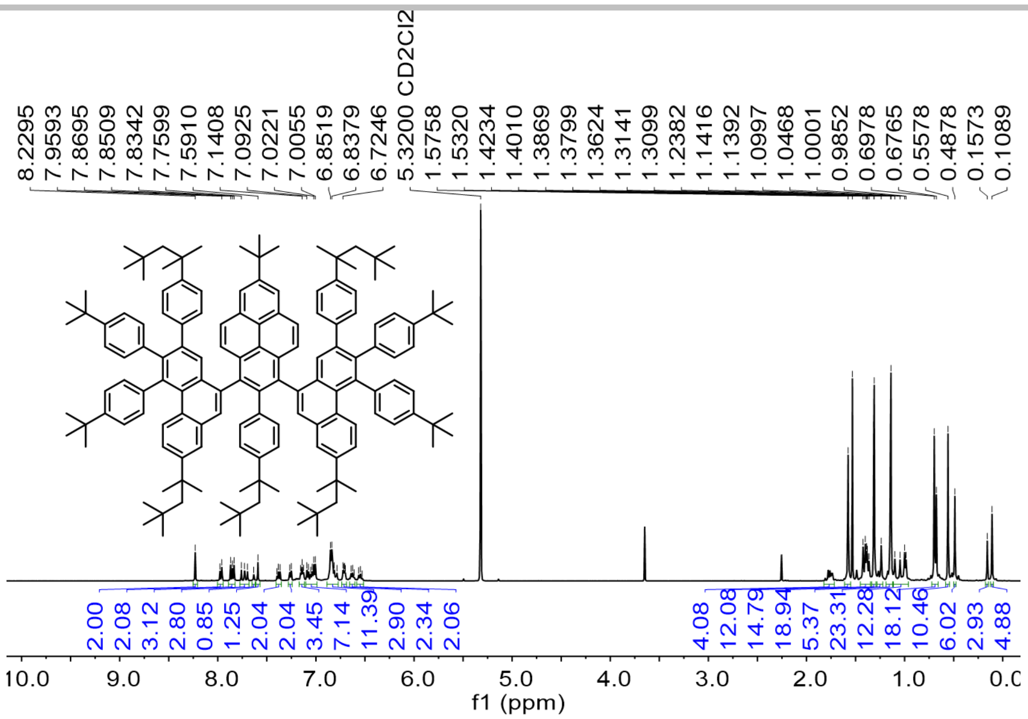

Figure S32. ^{1}H NMR spectrum of compound **13** (500 MHz, methylene chloride-d_2).

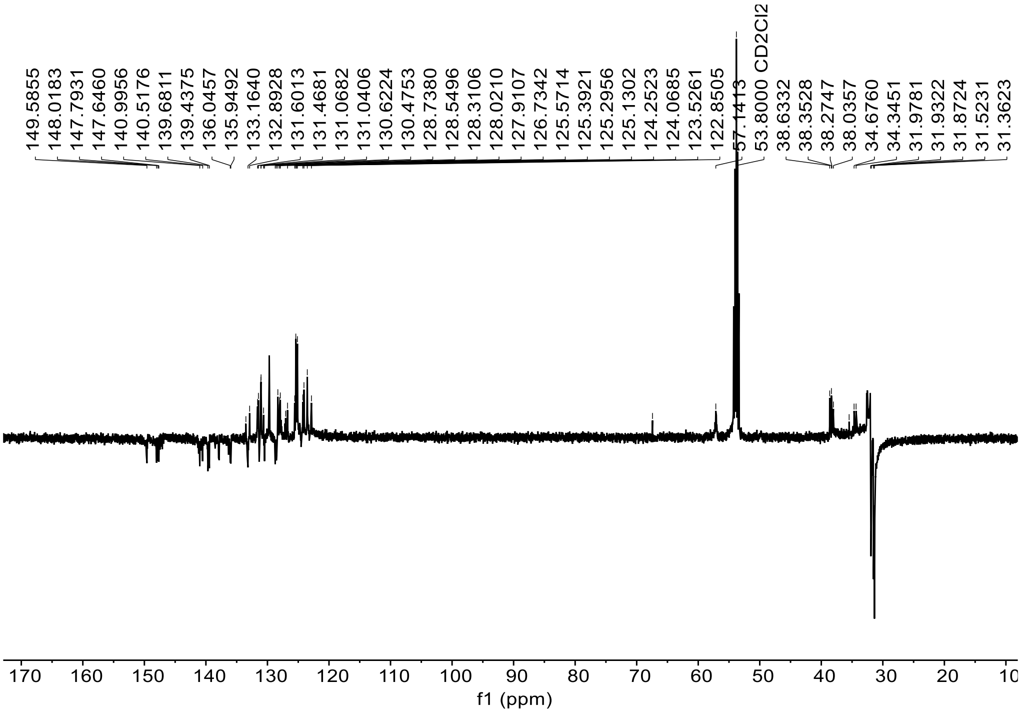

Figure S33. Spin-echo ^{13}C NMR spectrum of compound **13** (126 MHz, methylene chloride-d_2).

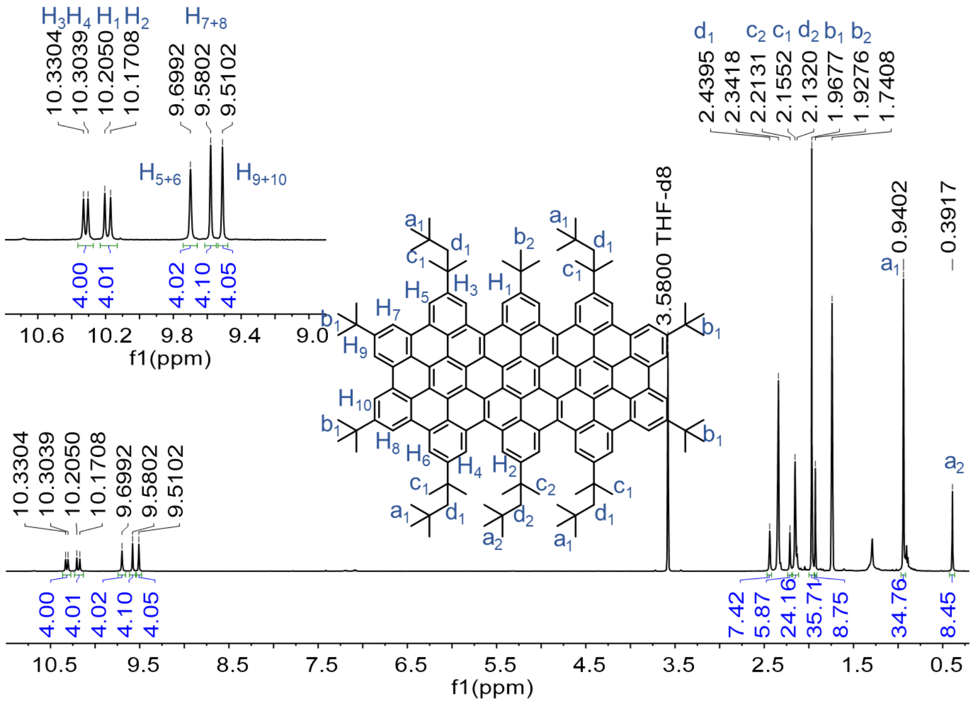

Figure S34. ^{1}H NMR spectrum of **HBPO** (500 MHz, THF-d_8:CS_2=2:1, 298K).

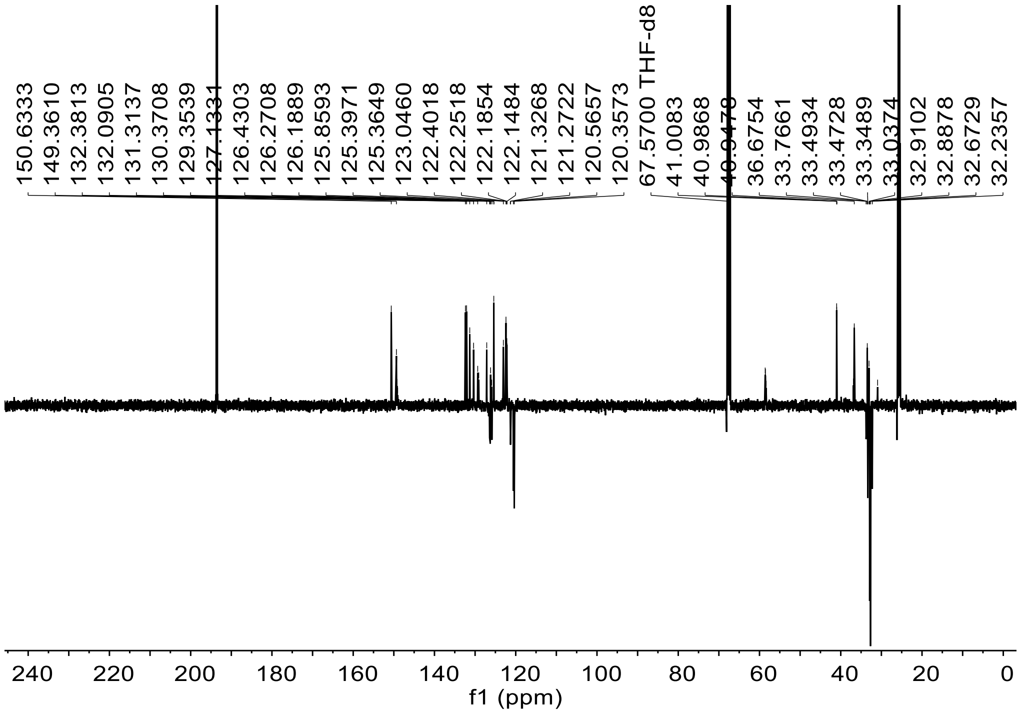

Figure S35. Spin-echo ^{13}C NMR spectrum of **HBPO** (126 MHz, THF-d_8:CS_2=2:1, 298K).

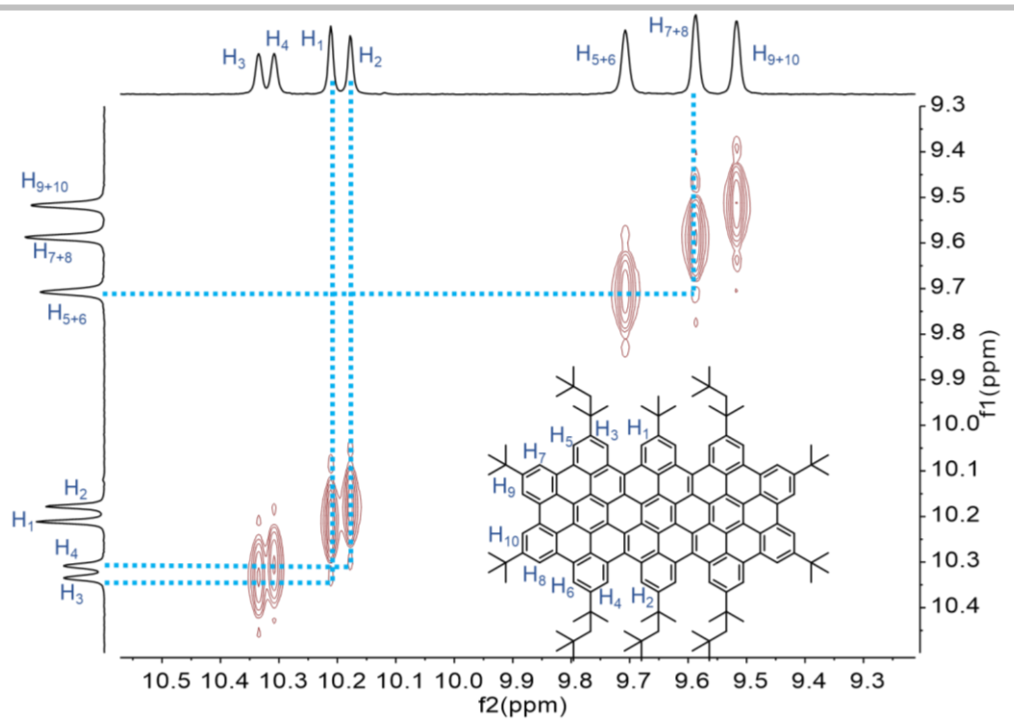

Figure S36. Aromatic region of the ^1H-^1H COSY spectrum of **HBPO** (500 MHz, THF-d_8:CS_2=2:1, 298K).

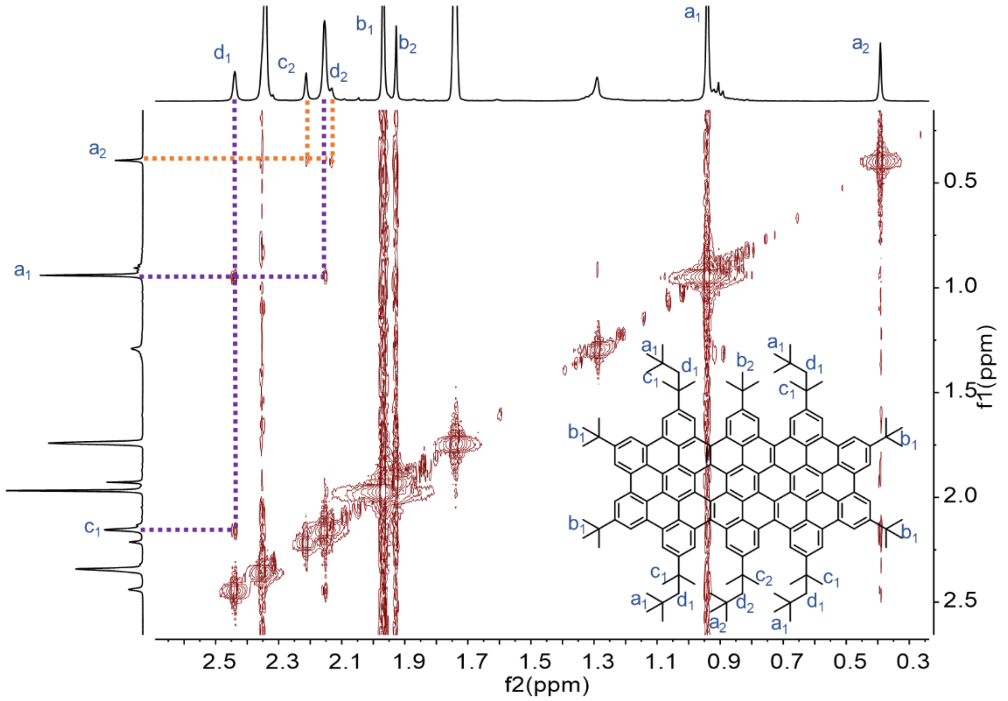

Figure S37. Aliphatic and aromatic region of the ^1H-^1H ROESY spectrum of **HBPO** (500 MHz, THF-d_8:CS_2=2:1, 298K).

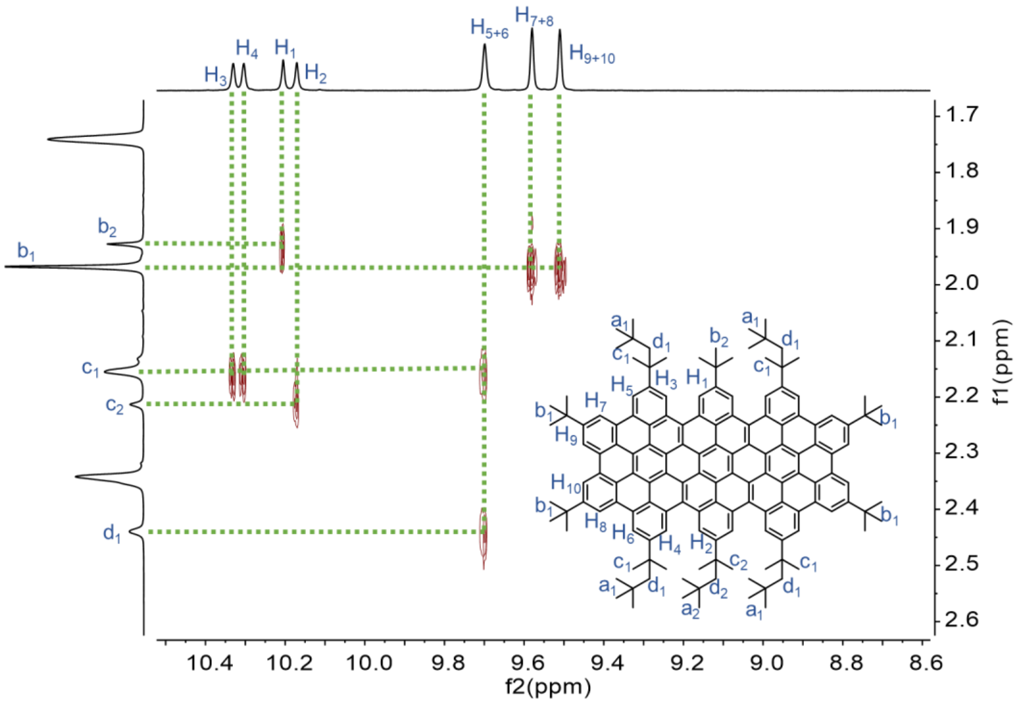

Figure S38. Aromatic region of the ^{1}H-^{1}H ROESY spectrum of **HBPO** (500 MHz, THF-d_8:CS_2=2:1, 298K).

Acquisition Parameter

Source Type	APCI	Ion Polarity	Positive	Set Nebulizer	3.0 Bar
Focus	Not active	Set Capillary	4500 V	Set Dry Heater	200 °C
Scan Begin	300 m/z	Set End Plate Offset	-500 V	Set Dry Gas	6.0 l/min
Scan End	2500 m/z	Set Collision Cell RF	1000.0 Vpp	Set Divert Valve	Waste

Meas. m/z	#	Formula	m/z	err [ppm]	rdb	e⁻ Conf	N-Rule
1904.1802	1	C 146 H 151	1904.1810	0.4	71.5	even	ok

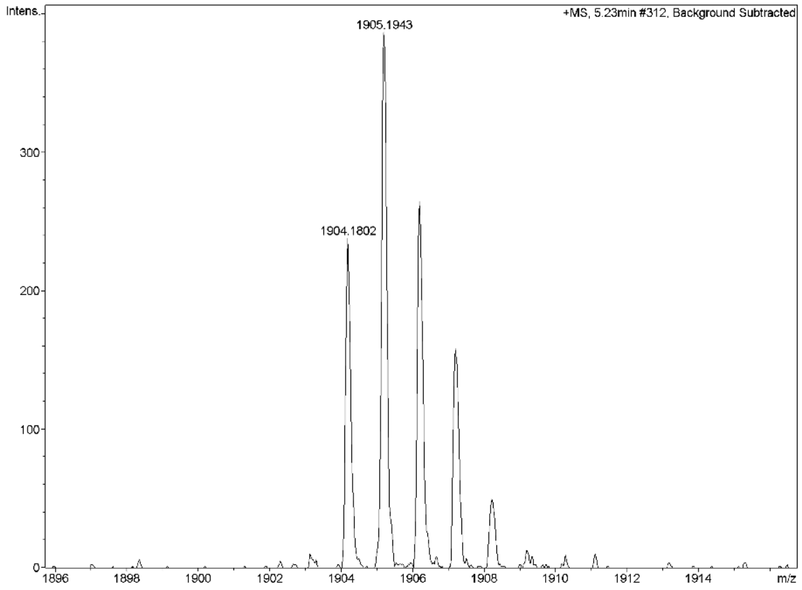

Figure S39. High-resolution APCI mass spectrum of compound [**HBPO**+H]$^{+}$.

8. Cartesian coordinates of Waggling Conformation, Butterfly Confirmation, Helical Conformation, Transition States (TS1 and TS2) of HBPO, Dibenzo-peri-octacene, and peri-Octacene.

Table S4. Cartesian coordinates of the waggling confirmation of **HBPO**.

	X	Y	Z
C	11.30332	4.435102	-1.036537
C	11.274342	4.478286	1.48772
C	12.456004	2.643605	0.270624
C	11.221594	3.564582	0.240058
C	12.340922	-2.922965	0.427029
C	11.18487	-4.687196	-0.913481
C	11.025026	-4.68927	1.60633
C	11.073279	-3.794077	0.345103
C	4.865101	9.566442	-1.520561
C	5.522519	7.248033	-2.249355
C	7.268813	8.960159	-1.732209
C	5.879988	8.420621	-1.318195
C	6.008024	8.00117	0.180636
C	3.55055	7.773497	0.908207
C	5.39694	7.136263	2.436662
C	4.951634	7.135744	0.946666
C	4.785644	-5.586431	-3.537834
C	4.124201	-7.969668	-4.000655
C	6.497457	-7.265273	-4.249087
C	5.218902	-7.058025	-3.404199
C	5.598275	-7.463798	-1.943519
C	3.292893	-7.689655	-0.839925
C	5.372629	-7.89056	0.505061
C	4.721558	-7.136404	-0.687581
C	-1.193986	6.994579	-3.467787
C	0.942217	7.675216	-2.366112
C	0.9656	6.051895	-4.295209
C	0.19516	6.495318	-3.027705
C	1.669944	-4.984234	6.229115
C	-0.026672	-3.710716	4.905231
C	-0.718031	-5.677745	6.314699
C	0.3802	-5.116169	5.385628

C	0.729403	-6.093168	4.218192
C	0.453471	-7.510679	2.18833
C	-1.600754	-6.744467	3.350188
C	-0.173836	-6.31692	2.957409
C	-4.795486	5.983604	3.333229
C	-6.505082	7.688374	3.984611
C	-4.139364	8.390323	3.66436
C	-5.238263	7.444795	3.131859
C	-5.640988	7.775554	1.658902
C	-5.458142	8.077022	-0.811467
C	-3.354007	7.945416	0.501879
C	-4.785258	7.384988	0.406586
C	-4.996502	-5.572173	-3.550198
C	-6.737733	-7.238077	-4.219159
C	-4.392517	-7.993365	-3.870969
C	-5.475545	-7.022255	-3.352145
C	-5.902931	-7.341809	-1.883706
C	-5.762937	-7.642536	0.588803
C	-3.638308	-7.584406	-0.698311
C	-5.051047	-6.976178	-0.621474
C	-11.234378	4.949088	0.807475
C	-11.113508	4.844372	-1.712405
C	-12.412288	3.131257	-0.438822
C	-11.143001	4.003446	-0.413869
C	-11.359352	-4.176464	1.255294
C	-11.371732	-4.289565	-1.267081
C	-12.530268	-2.419502	-0.081815
C	-11.297078	-3.341907	-0.046113
C	2.504572	2.873058	-0.357661
C	2.474549	1.485755	-0.151414
C	2.409598	-1.369603	0.212856
C	2.37541	-2.757585	0.412151
C	-2.439842	2.964741	-0.488161
C	-2.475491	1.580599	-0.263479
C	-2.539682	-1.267464	0.135458
C	-2.567811	-2.652032	0.359469
C	9.850723	1.383441	0.244721
C	9.898221	2.775585	0.237161

C	8.680132	3.465681	0.222417
C	7.44464	2.812674	0.213143
C	7.417346	1.389285	0.178031
C	8.641412	0.66771	0.214109
C	9.784865	-2.953138	0.264249
C	9.792512	-1.560156	0.260663
C	8.612822	-0.797486	0.211757
C	7.36147	-1.470056	0.164804
C	7.335197	-2.893672	0.126068
C	8.542439	-3.594408	0.191582
C	6.179559	3.55802	0.227622
C	6.136422	4.932646	0.462857
C	4.937153	5.661122	0.487655
C	3.762935	4.977428	0.183084
C	3.739654	3.58973	-0.087167
C	4.956202	2.855075	0.032255
C	6.13124	-0.720829	0.14278
C	6.15941	0.689993	0.12042
C	4.940507	1.422429	0.024606
C	3.698157	0.742214	0.009263
C	3.667723	-0.675191	0.112411
C	4.881114	-1.404198	0.161678
C	5.959481	-4.960937	-0.209308
C	6.044939	-3.588412	0.027422
C	4.841227	-2.83613	0.146842
C	3.591698	-3.521139	0.193398
C	3.572612	-4.905727	-0.090635
C	4.73397	-5.633486	-0.335806
C	1.286521	3.529074	-0.820609
C	1.291136	4.720286	-1.565002
C	0.113217	5.306083	-2.049884
C	-1.103767	4.756417	-1.643608
C	-1.176871	3.569282	-0.88879
C	0.035239	2.87268	-0.616928
C	1.186002	-0.613506	0.121222
C	1.218561	0.779403	-0.10802
C	-0.000612	1.497065	-0.244908
C	-1.251488	0.828356	-0.15919

C	-1.283903	-0.564067	0.078271
C	-0.065012	-1.281934	0.210772
C	1.047111	-4.559339	1.563798
C	1.116125	-3.36606	0.825433
C	-0.098574	-2.657833	0.581132
C	-1.345885	-3.306809	0.806222
C	-1.346433	-4.496418	1.561035
C	-0.168145	-5.093242	2.015418
C	-3.658533	3.732779	-0.299429
C	-3.640684	5.126033	-0.065998
C	-4.801843	5.86625	0.133684
C	-6.026965	5.189139	0.035257
C	-6.111911	3.807094	-0.141963
C	-4.908496	3.05012	-0.235576
C	-3.765321	-0.526303	-0.022862
C	-3.7347	0.889001	-0.151177
C	-4.948462	1.617728	-0.209804
C	-6.198886	0.9357	-0.162666
C	-6.228211	-0.474586	-0.100842
C	-5.008655	-1.205891	-0.008769
C	-3.837247	-4.76304	-0.110763
C	-3.808322	-3.370454	0.124869
C	-5.026965	-2.638686	0.017799
C	-6.255371	-3.347566	-0.117685
C	-6.219242	-4.732415	-0.290862
C	-5.019193	-5.457293	-0.349971
C	-7.429092	1.68512	-0.19439
C	-7.402607	3.109291	-0.205663
C	-8.610653	3.807663	-0.282911
C	-9.853828	3.165197	-0.318026
C	-9.861393	1.773312	-0.265021
C	-8.681274	1.012339	-0.203534
C	-8.755697	-3.247935	-0.071541
C	-7.518544	-2.598563	-0.102047
C	-7.487707	-1.174362	-0.115025
C	-8.711299	-0.451988	-0.154368
C	-9.922355	-1.165111	-0.140312
C	-9.972458	-2.556026	-0.087391

H	10.474239	5.145926	-1.099195
H	11.277436	3.813072	-1.937045
H	12.235994	5.010259	-1.046654
H	10.446805	5.193318	1.504779
H	12.208453	5.050974	1.501808
H	11.223514	3.887582	2.408084
H	13.367352	3.249888	0.274067
H	12.499143	1.989998	-0.606755
H	12.474666	2.016111	1.167777
H	13.225419	-3.565248	0.484342
H	12.339877	-2.284009	1.316195
H	12.455198	-2.284273	-0.454966
H	12.092848	-5.298979	-0.867951
H	11.229943	-4.078891	-1.822607
H	10.332044	-5.365566	-1.008954
H	10.948873	-4.082283	2.514231
H	11.934881	-5.295806	1.67761
H	10.171463	-5.373181	1.590608
H	5.088557	10.41335	-0.861388
H	3.835085	9.25839	-1.330955
H	4.910453	9.931335	-2.553034
H	4.527178	6.846775	-2.040397
H	6.234497	6.422863	-2.150496
H	5.535323	7.579572	-3.294161
H	8.033955	8.178201	-1.669379
H	7.583222	9.789238	-1.087583
H	7.252152	9.328544	-2.763876
H	6.119477	8.930443	0.756377
H	6.971956	7.489387	0.285522
H	3.084701	7.722274	-0.077509
H	3.612817	8.828083	1.193126
H	2.877948	7.281693	1.618574
H	5.361816	8.151507	2.847558
H	6.41762	6.760988	2.557389
H	4.73588	6.502548	3.035962
H	5.551606	-4.904057	-3.157192
H	3.860197	-5.377314	-2.994806
H	4.613826	-5.341906	-4.592504

H	3.149784	-7.825203	-3.530105
H	4.391908	-9.028148	-3.901508
H	4.002176	-7.759145	-5.069216
H	6.860328	-8.297427	-4.177467
H	7.304605	-6.602542	-3.917527
H	6.304564	-7.053352	-5.306457
H	5.734273	-8.554239	-1.948223
H	6.596676	-7.055486	-1.749315
H	2.701354	-7.141364	-1.57635
H	2.753361	-7.653827	0.110971
H	3.331652	-8.73707	-1.15373
H	4.841144	-7.670808	1.436555
H	6.419698	-7.604619	0.644048
H	5.340176	-8.973435	0.341052
H	-1.768148	6.213059	-3.975432
H	-1.078521	7.827119	-4.169108
H	-1.785797	7.358662	-2.621578
H	1.000693	8.524291	-3.05618
H	1.965616	7.402854	-2.093944
H	0.428009	8.009244	-1.458905
H	1.982612	5.72538	-4.060157
H	1.037131	6.882071	-5.00703
H	0.454788	5.220902	-4.792049
H	2.010995	-5.960764	6.592656
H	1.49888	-4.345126	7.102346
H	2.484086	-4.541041	5.645123
H	-0.164663	-3.046925	5.766644
H	-0.963603	-3.721138	4.342131
H	0.739554	-3.267789	4.261763
H	-1.700704	-5.707759	5.839946
H	-0.809805	-5.049918	7.208213
H	-0.474392	-6.69309	6.648706
H	1.719301	-5.803352	3.846747
H	0.874771	-7.082853	4.673895
H	-0.072315	-7.685382	1.24405
H	0.395108	-8.427118	2.785991
H	1.507873	-7.332207	1.959091
H	-2.158533	-7.100678	2.477585

H	-2.177137	-5.936412	3.805431
H	-1.556896	-7.569155	4.06773
H	-4.618402	5.790432	4.397682
H	-3.870381	5.753515	2.797991
H	-5.558538	5.279256	2.98846
H	-6.875229	8.713467	3.865495
H	-6.294765	7.532283	5.048318
H	-7.312944	7.005002	3.700067
H	-3.17351	8.232206	3.180428
H	-3.993805	8.229415	4.738588
H	-4.418051	9.441129	3.522989
H	-5.780545	8.864573	1.609959
H	-6.640875	7.354902	1.500592
H	-6.507224	7.784719	-0.917463
H	-4.942779	7.810776	-1.739843
H	-5.424009	9.166745	-0.70236
H	-3.38671	9.010477	0.750117
H	-2.829134	7.84846	-0.454367
H	-2.751171	7.443668	1.261727
H	-4.79883	-5.386178	-4.612291
H	-4.074776	-5.362152	-3.001091
H	-5.748178	-4.84938	-3.218934
H	-6.512102	-7.086527	-5.280395
H	-7.533776	-6.537322	-3.943642
H	-7.13154	-8.254878	-4.104615
H	-3.42788	-7.853871	-3.378938
H	-4.23373	-7.839689	-4.944378
H	-4.694892	-9.037409	-3.728401
H	-6.072872	-8.426566	-1.836781
H	-6.893181	-6.895214	-1.736121
H	-5.766054	-8.732979	0.481082
H	-6.802102	-7.31249	0.681058
H	-5.250179	-7.393937	1.523494
H	-3.009018	-7.100354	-1.448137
H	-3.702697	-8.647085	-0.950547
H	-3.123758	-7.507736	0.26529
H	-12.144698	5.556609	0.752463
H	-10.38207	5.632787	0.858643

H	-11.261487	4.379705	1.742148
H	-10.257416	5.524721	-1.739094
H	-12.022499	5.450914	-1.79431
H	-11.054209	4.199555	-2.59512
H	-13.297122	3.771766	-0.509374
H	-12.512978	2.530303	0.470924
H	-12.425885	2.455539	-1.300266
H	-11.318926	-3.529609	2.137603
H	-10.530016	-4.886324	1.324924
H	-12.292285	-4.749871	1.296006
H	-10.54619	-5.007035	-1.277608
H	-11.334162	-3.724451	-2.204007
H	-12.307266	-4.859877	-1.250697
H	-12.563533	-1.817814	-0.996047
H	-12.557149	-1.741101	0.777201
H	-13.44253	-3.023708	-0.052083
H	10.780949	0.835476	0.263142
H	8.70086	4.548005	0.197858
H	10.742458	-1.048788	0.308478
H	8.519853	-4.676842	0.206445
H	7.058625	5.449263	0.694059
H	2.819378	5.501599	0.213453
H	6.873134	-5.522332	-0.355156
H	2.611566	-5.390681	-0.179221
H	2.246208	5.14908	-1.840483
H	-2.028274	5.202588	-1.979044
H	1.975089	-5.030912	1.859552
H	-2.300436	-4.905479	1.859306
H	-2.680895	5.613449	0.014299
H	-6.940291	5.757543	0.153628
H	-2.894717	-5.286836	-0.159012
H	-7.149836	-5.264301	-0.439676
H	-8.588579	4.888836	-0.335663
H	-10.811862	1.261132	-0.28345
H	-8.779745	-4.329003	-0.017621
H	-10.851734	-0.615885	-0.160729

Table S5. Cartesian coordinates of the butterfly confirmation of **HBPO**.

	X	Y	Z
C	-11.266588	4.762882	-0.483758
C	-12.35847	2.911344	0.791037
C	-11.065374	4.652781	2.030948
C	-11.113357	3.816105	0.730296
C	-12.021021	-2.983996	-0.91247
C	-10.935044	-4.996325	0.095117
C	-11.819139	-3.203635	1.594854
C	-11.116338	-3.473964	0.243154
C	-4.859694	5.818116	-3.254976
C	-4.226462	8.218102	-3.668387
C	-6.585182	7.483155	-3.965438
C	-5.316612	7.280898	-3.104358
C	-5.724991	7.656508	-1.644062
C	-5.553941	8.049433	0.813286
C	-3.444545	7.929096	-0.490645
C	-4.860149	7.333136	-0.379028
C	-4.766036	-4.837581	-3.981056
C	-6.495935	-6.35582	-4.958846
C	-4.14293	-7.142644	-4.777735
C	-5.229949	-6.303253	-4.072098
C	-5.644645	-6.908444	-2.69225
C	-5.469205	-7.701395	-0.333211
C	-3.361631	-7.341961	-1.597969
C	-4.783743	-6.790159	-1.388963
C	-1.266452	7.007998	3.778781
C	0.246051	8.012988	2.048449
C	1.201457	6.814413	4.047965
C	0.05731	6.814496	3.008493
C	-0.053777	-8.403716	5.174889
C	-2.408732	-7.833231	4.507325
C	-1.208795	-6.432964	6.181935
C	-1.028958	-7.248542	4.879704
C	-0.515531	-6.226198	3.813191
C	-0.836783	-7.616109	1.626342
C	1.457033	-7.222121	2.510929
C	0.047235	-6.606288	2.393852
C	5.082024	8.281216	-3.736988

C	2.712281	7.523896	-3.694594
C	4.537524	5.87435	-3.243516
C	4.092716	7.332687	-3.024864
C	3.899279	7.703352	-1.51922
C	6.330329	7.928355	-0.722856
C	4.448655	8.089153	0.885404
C	4.935028	7.360866	-0.397059
C	4.973989	-4.893992	-3.890931
C	4.344798	-7.215606	-4.632919
C	6.698397	-6.436303	-4.840273
C	5.435568	-6.361944	-3.950558
C	5.855016	-6.935368	-2.558932
C	5.690555	-7.675262	-0.181897
C	3.577676	-7.360229	-1.447547
C	4.996467	-6.793009	-1.256581
C	11.128374	5.350299	0.684971
C	12.034966	3.382649	1.929974
C	12.17714	3.474103	-0.589388
C	11.302724	3.820557	0.639258
C	11.132299	-4.938682	0.201188
C	12.203008	-2.942665	-0.854168
C	12.013112	-3.109522	1.658208
C	11.306007	-3.412551	0.315696
C	-2.400552	3.013176	0.70735
C	-2.397776	1.618439	0.558427
C	-2.386743	-1.256337	0.382735
C	-2.379262	-2.658911	0.358958
C	2.550436	3.03245	0.71714
C	2.556644	1.636828	0.571803
C	2.56285	-1.241042	0.41097
C	2.562312	-2.644162	0.400548
C	-9.780803	1.619965	0.522873
C	-9.80662	3.011585	0.59124
C	-8.581688	3.686097	0.524991
C	-7.359729	3.017667	0.409322
C	-7.350361	1.594426	0.393774
C	-8.584381	0.890213	0.422487
C	-9.774954	-2.715776	0.20227

C	-9.761702	-1.32304	0.321698
C	-8.578434	-0.572932	0.323308
C	-7.335376	-1.256981	0.202577
C	-7.331225	-2.666473	0.025349
C	-8.547847	-3.362642	0.048081
C	-6.088919	3.745569	0.308245
C	-6.04275	5.121227	0.078952
C	-4.83567	5.825248	-0.051599
C	-3.655099	5.123369	0.172635
C	-3.634881	3.740526	0.463989
C	-4.865854	3.023502	0.422217
C	-6.093485	-0.529523	0.268249
C	-6.100132	0.877897	0.361599
C	-4.867946	1.590658	0.427109
C	-3.63509	0.891092	0.422802
C	-3.628967	-0.527575	0.333116
C	-4.854811	-1.233229	0.245338
C	-5.991668	-4.687998	-0.599785
C	-6.054474	-3.361482	-0.170358
C	-4.839291	-2.652252	0.052331
C	-3.602789	-3.359636	0.011212
C	-3.604819	-4.689193	-0.468463
C	-4.776334	-5.355014	-0.819676
C	-1.161445	3.670768	1.103106
C	-1.135033	4.901397	1.78386
C	0.05744	5.509679	2.185197
C	1.259763	4.918134	1.779987
C	1.3046	3.683664	1.106868
C	0.074512	2.989986	0.901103
C	-1.149736	-0.520536	0.467827
C	-1.154595	0.888727	0.552637
C	0.079645	1.592165	0.621582
C	1.318265	0.897903	0.56171
C	1.322001	-0.512376	0.480652
C	0.088436	-1.217839	0.456652
C	-1.10418	-4.644114	1.233733
C	-1.13916	-3.350098	0.689748
C	0.091839	-2.638251	0.576212

C	1.322488	-3.339778	0.716267
C	1.29002	-4.634982	1.270915
C	0.093182	-5.275003	1.600319
C	3.782716	3.766515	0.469036
C	3.800379	5.145202	0.177489
C	4.979125	5.851332	-0.072032
C	6.185615	5.153261	0.041417
C	6.235928	3.776634	0.288965
C	5.018984	3.052707	0.422127
C	3.801251	-0.505199	0.362941
C	3.798808	0.913799	0.441646
C	5.028275	1.619849	0.43863
C	6.264048	0.913571	0.375779
C	6.265796	-0.495612	0.29934
C	5.031897	-1.20574	0.284734
C	3.80417	-4.677846	-0.389907
C	3.792762	-3.342181	0.07038
C	5.024905	-2.627825	0.108737
C	6.244306	-3.333877	-0.100062
C	6.190839	-4.668341	-0.507073
C	4.980548	-5.345612	-0.719799
C	7.5095	1.638183	0.390826
C	7.51017	3.058325	0.383615
C	8.731772	3.74219	0.474478
C	9.956617	3.077823	0.534491
C	9.937277	1.680203	0.491031
C	8.750825	0.94058	0.419711
C	8.737265	-3.318128	0.120022
C	7.51651	-2.628879	0.086251
C	7.512522	-1.217053	0.241344
C	8.752102	-0.525055	0.34808
C	9.939156	-1.267808	0.358951
C	9.96051	-2.662645	0.263468
H	-10.433113	5.467258	-0.55875
H	-12.189493	5.347358	-0.397805
H	-11.308994	4.195993	-1.419394
H	-13.256662	3.528655	0.89319
H	-12.327227	2.231479	1.648758

H	-12.473152	2.31258	-0.118504
H	-10.226819	5.355049	2.034399
H	-10.962202	4.006391	2.908473
H	-11.986791	5.235165	2.143595
H	-12.228364	-1.912186	-0.843193
H	-12.982108	-3.510036	-0.892849
H	-11.551399	-3.170237	-1.883745
H	-11.912266	-5.488396	0.128712
H	-10.327934	-5.415635	0.904059
H	-10.46674	-5.260548	-0.858686
H	-12.021121	-2.139006	1.744401
H	-11.202178	-3.546225	2.431831
H	-12.777141	-3.733703	1.638642
H	-5.618184	5.118722	-2.890371
H	-3.935564	5.615277	-2.707452
H	-4.675549	5.591242	-4.311496
H	-3.257473	8.079901	-3.184722
H	-4.511318	9.270769	-3.556268
H	-4.084281	8.027083	-4.738103
H	-6.964049	8.508753	-3.883432
H	-7.387581	6.803861	-3.656673
H	-6.373168	7.290596	-5.02283
H	-6.714726	7.218847	-1.468751
H	-5.89003	8.74298	-1.635585
H	-5.546159	9.135314	0.667292
H	-5.035195	7.827992	1.751598
H	-6.595815	7.735257	0.926969
H	-2.924003	7.882269	0.471718
H	-3.504993	8.982748	-0.779158
H	-2.823633	7.416248	-1.228141
H	-4.580781	-4.440622	-4.98598
H	-3.841337	-4.730987	-3.407768
H	-5.521738	-4.204903	-3.505709
H	-6.878602	-7.379275	-5.04813
H	-6.277484	-5.992428	-5.969025
H	-7.297275	-5.733036	-4.545757
H	-4.433676	-8.197945	-4.838237
H	-3.175165	-7.089893	-4.275719

H	-3.99623	-6.782119	-5.802263
H	-5.807837	-7.982545	-2.858172
H	-6.635956	-6.505473	-2.45425
H	-6.515196	-7.423244	-0.172576
H	-4.954508	-7.632249	0.63049
H	-5.447729	-8.748079	-0.65663
H	-2.836989	-7.447993	-0.643887
H	-2.750867	-6.706501	-2.242973
H	-3.410862	-8.334529	-2.055863
H	-2.122089	7.148972	3.111643
H	-1.199119	7.90288	4.405821
H	-1.481093	6.154899	4.430422
H	0.255354	8.957082	2.605337
H	-0.564404	8.059884	1.313958
H	1.188347	7.937757	1.497519
H	1.149652	7.72301	4.65731
H	2.190996	6.796203	3.583426
H	1.127969	5.952234	4.718515
H	0.949005	-8.035018	5.415579
H	0.030984	-9.096919	4.333044
H	-0.40772	-8.983453	6.035291
H	-2.363573	-8.4991	3.643159
H	-3.128292	-7.03765	4.282621
H	-2.81122	-8.413943	5.345259
H	-1.885864	-5.584911	6.029723
H	-0.252067	-6.035657	6.53874
H	-1.630269	-7.057931	6.977136
H	-1.345644	-5.527754	3.657681
H	0.273586	-5.627957	4.287996
H	-1.883496	-7.306378	1.57935
H	-0.804813	-8.602988	2.096232
H	-0.473266	-7.728045	0.599269
H	1.416559	-8.203334	2.984738
H	2.130889	-6.597678	3.105506
H	1.906047	-7.359617	1.52194
H	4.801771	9.330624	-3.588243
H	5.076236	8.088448	-4.815847
H	6.110716	8.157362	-3.392728

H	2.773629	7.335631	-4.772056
H	2.340351	8.545694	-3.555302
H	1.968536	6.836417	-3.276821
H	4.575915	5.652248	-4.31635
H	3.842681	5.167343	-2.780601
H	5.530363	5.677044	-2.829799
H	2.939427	7.27689	-1.205864
H	3.75007	8.791532	-1.48104
H	6.246711	8.981659	-1.00658
H	6.821617	7.399551	-1.542734
H	6.989668	7.878494	0.150251
H	4.4532	9.175305	0.74021
H	5.100814	7.855773	1.733255
H	3.43152	7.791687	1.15533
H	4.051273	-4.773819	-3.317289
H	4.786531	-4.518843	-4.903799
H	5.731918	-4.252183	-3.431534
H	4.631662	-8.273158	-4.666716
H	4.197824	-6.881043	-5.666156
H	3.377785	-7.146392	-4.131303
H	6.477145	-6.094434	-5.857339
H	7.07916	-7.462089	-4.908542
H	7.502185	-5.806081	-4.443501
H	6.844494	-6.522024	-2.331108
H	6.023409	-8.011979	-2.702051
H	6.735247	-7.387161	-0.030795
H	5.674766	-8.72924	-0.481107
H	5.177631	-7.58641	0.781034
H	3.632554	-8.363077	-1.881651
H	2.957839	-6.745445	-2.103631
H	3.059007	-7.447889	-0.487443
H	12.109384	5.83021	0.75968
H	10.539463	5.668394	1.551499
H	10.643494	5.73298	-0.21906
H	12.232565	2.306817	1.942602
H	11.440769	3.622442	2.817654
H	12.997932	3.898557	2.015271
H	12.376365	2.400792	-0.659296

H	13.142845	3.988385	-0.527178
H	11.686616	3.7829	-1.518188
H	10.5296	-5.343482	1.020767
H	12.112107	-5.424996	0.242234
H	10.662613	-5.225834	-0.745266
H	11.731239	-3.154675	-1.819103
H	13.168143	-3.460874	-0.826153
H	12.402162	-1.867964	-0.810293
H	12.973442	-3.634637	1.709649
H	11.401032	-3.435889	2.505168
H	12.211717	-2.041126	1.783594
H	-10.717498	1.083248	0.554074
H	-8.582676	4.767218	0.587822
H	-10.709561	-0.810483	0.422265
H	-8.529703	-4.440098	-0.032863
H	-6.972688	5.657647	-0.059567
H	-2.70866	5.63179	0.069502
H	-6.915209	-5.203344	-0.829934
H	-2.651511	-5.173004	-0.621205
H	-2.077789	5.34409	2.068553
H	2.195584	5.379903	2.060608
H	-2.045709	-5.130534	1.452829
H	2.232056	-5.103936	1.513066
H	2.853184	5.658743	0.092503
H	7.112943	5.681723	-0.124361
H	2.854352	-5.167737	-0.542729
H	7.118119	-5.182629	-0.724125
H	8.719722	4.821502	0.521718
H	10.883655	1.155576	0.51814
H	8.724882	-4.39679	0.056801
H	10.88392	-0.747959	0.451627

Table S6. Cartesian coordinates of the helical confirmation of **HBPO**.

	X	Y	Z
C	9.9083	-2.44929	0.61277
C	9.85321	-1.07968	0.86055
C	8.63948	-0.36588	0.78629

C	7.44799	-1.06035	0.46946
C	7.50414	-2.4479	0.15882
C	8.72582	-3.11842	0.24294
C	8.59324	1.0843	1.00333
C	9.76019	1.81528	1.30456
C	9.73027	3.19828	1.47006
C	8.50889	3.87409	1.29042
C	7.32985	3.18698	0.99223
C	7.35993	1.76918	0.8823
C	5.1313	-5.0373	-1.29915
C	6.31037	-4.40852	-0.83367
C	6.27489	-3.14434	-0.25601
C	5.0167	-2.48834	-0.09409
C	3.81746	-3.19351	-0.36452
C	3.90921	-4.42896	-1.05186
C	4.9658	-1.08756	0.24942
C	6.17764	-0.35898	0.46046
C	6.13419	1.02771	0.65198
C	4.87986	1.71513	0.61242
C	3.68209	0.99274	0.45625
C	3.72499	-0.4263	0.2929
C	4.84688	3.15711	0.66542
C	6.06087	3.90256	0.77629
C	6.02478	5.28793	0.65562
C	4.81832	5.97064	0.37762
C	3.64255	5.23752	0.30031
C	3.61133	3.84165	0.53721
C	0.15637	-5.50373	0.34116
C	1.33575	-4.75639	0.34163
C	1.31736	-3.35941	0.17866
C	0.0655	-2.68376	0.24854
C	-1.1497	-3.42831	0.34664
C	-1.07126	-4.82596	0.28533
C	0.02943	-1.24218	0.23171
C	1.23724	-0.49985	0.27245
C	1.19725	0.91201	0.3461
C	-0.05044	1.57901	0.28102
C	-1.25388	0.83924	0.15163

C	-1.21871	-0.57074	0.1829
C	-0.11246	3.00941	0.45436
C	1.06668	3.73082	0.78743
C	0.92052	5.01361	1.34513
C	-0.3224	5.64562	1.41867
C	-1.45561	4.9765	0.92995
C	-1.37108	3.67719	0.41469
C	-4.94713	-5.26687	1.70743
C	-3.74631	-4.60245	1.49238
C	-3.6845	-3.37853	0.7848
C	-4.9079	-2.73951	0.4536
C	-6.14033	-3.44564	0.59765
C	-6.13679	-4.70191	1.19505
C	-4.91195	-1.35147	0.05943
C	-3.70223	-0.63518	0.0428
C	-3.72195	0.77717	-0.16787
C	-4.93442	1.43262	-0.44964
C	-6.15224	0.68544	-0.51461
C	-6.14456	-0.68757	-0.23352
C	-4.9379	2.85436	-0.70008
C	-3.73759	3.60028	-0.5746
C	-3.72478	4.9456	-1.01516
C	-4.88335	5.58832	-1.42878
C	-6.09467	4.85925	-1.44691
C	-6.13892	3.51014	-1.10938
C	-8.58729	-3.53059	0.09065
C	-7.39051	-2.81292	0.14395
C	-7.38426	-1.44112	-0.23469
C	-8.59673	-0.81143	-0.60397
C	-9.78405	-1.5711	-0.63958
C	-9.79194	-2.92557	-0.31495
C	-8.59931	0.61884	-0.93135
C	-7.39046	1.35328	-0.86969
C	-7.39553	2.74371	-1.17372
C	-8.59823	3.35914	-1.52891
C	-9.80665	2.64026	-1.59112
C	-9.78981	1.27873	-1.29883
C	2.54403	-2.58067	-0.00027

C	2.5049	-1.20297	0.19161
C	2.41549	1.69765	0.46854
C	2.36708	3.08263	0.59823
C	-2.42572	-2.71422	0.45522
C	-2.4498	-1.34265	0.22544
C	-2.50735	1.55211	-0.02996
C	-2.54151	2.94076	-0.06909
C	11.21915	-3.24021	0.68601
C	12.3333	-2.45298	1.40178
C	10.98964	-4.554	1.46005
C	11.66472	-3.54642	-0.75774
C	10.99181	4.00681	1.79249
C	12.1362	3.11269	2.30673
C	10.67816	5.05288	2.88117
C	11.44102	4.7101	0.49632
C	5.26547	-6.37301	-2.0548
C	5.89776	-7.37631	-1.07303
C	3.88781	-6.91673	-2.48272
C	6.1133	-6.09843	-3.33041
C	6.98255	-7.22033	-3.96135
C	6.25893	-8.57488	-4.03196
C	7.32175	-6.77964	-5.40747
C	8.309	-7.37539	-3.18881
C	4.84781	7.49435	0.21996
C	3.46976	8.08192	-0.1233
C	5.25992	8.06657	1.60279
C	5.91564	7.93993	-0.81467
C	5.747	7.59951	-2.32271
C	4.67377	8.48584	-2.98476
C	7.10539	7.90394	-3.00456
C	5.40439	6.1225	-2.55934
C	0.16	-7.0324	0.46374
C	1.56402	-7.63527	0.30498
C	-0.31909	-7.34719	1.90494
C	-0.8431	-7.68954	-0.52177
C	-0.56587	-7.67517	-2.05142
C	0.43287	-8.78287	-2.44466
C	-0.04739	-6.31862	-2.54845

C	-1.91136	-7.9745	-2.75907
C	-0.48938	7.0555	1.99809
C	-1.51099	7.00296	3.15055
C	-0.99916	7.98369	0.87612
C	0.83254	7.63046	2.54073
C	-5.01388	-6.61808	2.42797
C	-5.18028	-7.68263	1.31228
C	-3.72496	-6.9375	3.19942
C	-6.24618	-6.71516	3.36762
C	-6.30677	-5.86477	4.66891
C	-7.78551	-5.85127	5.13213
C	-5.46377	-6.50474	5.79085
C	-5.84993	-4.41538	4.45547
C	-4.88816	7.04248	-1.91238
C	-5.18632	6.99341	-3.43374
C	-3.52563	7.72933	-1.73121
C	-6.01764	7.86303	-1.23296
C	-5.94412	8.2003	0.28292
C	-7.36248	8.65217	0.7141
C	-5.53751	6.99326	1.14004
C	-4.97297	9.36788	0.54988
C	-11.07451	-3.76426	-0.34311
C	-10.79771	-5.11407	-1.03547
C	-11.51502	-3.99739	1.11574
C	-12.21179	-3.06038	-1.10767
C	-11.08657	3.37209	-2.01007
C	-12.3509	2.53239	-1.74619
C	-10.98555	3.66943	-3.51932
C	-11.2128	4.68872	-1.217
H	10.76223	-0.53022	1.11531
H	8.76727	-4.18654	0.01968
H	10.70216	1.27088	1.40324
H	8.48445	4.96178	1.38822
H	7.26703	-4.91852	-0.9641
H	2.98608	-4.87921	-1.42229
H	6.94798	5.86394	0.74653
H	2.71262	5.73596	0.0194
H	2.29343	-5.2613	0.48142

H	-1.98446	-5.42049	0.18786
H	1.80121	5.50636	1.76218
H	-2.42913	5.46639	0.99823
H	-2.82039	-5.00967	1.90445
H	-7.07374	-5.25257	1.31108
H	-2.76688	5.46778	-1.05414
H	-7.01169	5.37232	-1.74787
H	-8.59123	-4.5867	0.36921
H	-10.71113	-1.06997	-0.92762
H	-8.60351	4.42609	-1.76507
H	-10.71058	0.69333	-1.35248
H	13.24987	-3.04854	1.47505
H	12.59187	-1.53316	0.86721
H	12.03969	-2.18236	2.422
H	11.92454	-5.1093	1.58638
H	10.58352	-4.35776	2.45884
H	10.28709	-5.21757	0.94571
H	12.60428	-4.10738	-0.77402
H	10.91803	-4.13926	-1.29573
H	11.81889	-2.62442	-1.32935
H	11.83906	2.56102	3.2055
H	12.45754	2.386	1.5535
H	13.01531	3.71153	2.56868
H	10.26675	4.57717	3.77867
H	11.5794	5.59799	3.17961
H	9.94985	5.79582	2.54062
H	11.65985	3.98364	-0.29421
H	10.66783	5.38298	0.11163
H	12.34488	5.30533	0.65871
H	5.37289	-7.37885	-0.11244
H	5.86183	-8.39922	-1.47697
H	6.95318	-7.14571	-0.88039
H	3.24157	-7.12494	-1.61449
H	3.35587	-6.21412	-3.13405
H	3.98837	-7.85282	-3.04251
H	6.78961	-5.23918	-3.14408
H	5.41446	-5.72417	-4.10731
H	5.28759	-8.48105	-4.52853

H	6.84471	-9.31248	-4.5889
H	6.08204	-8.98696	-3.02837
H	6.42363	-6.71183	-6.02863
H	7.81198	-5.80182	-5.42851
H	7.9974	-7.49209	-5.89041
H	8.91301	-6.46475	-3.24478
H	8.13781	-7.58928	-2.1276
H	8.91243	-8.19251	-3.59563
H	3.09973	7.70406	-1.08743
H	2.71613	7.85165	0.64852
H	3.52093	9.17313	-0.20734
H	5.24181	9.16047	1.60352
H	4.57594	7.72583	2.38784
H	6.26673	7.75472	1.89533
H	6.89227	7.52876	-0.48644
H	6.02242	9.04026	-0.733
H	4.846	9.54722	-2.78864
H	4.66264	8.3481	-4.07074
H	3.66945	8.23654	-2.61877
H	7.9034	7.26666	-2.61264
H	7.0533	7.72472	-4.08319
H	7.40563	8.94537	-2.8623
H	6.11042	5.45548	-2.05198
H	4.40395	5.87034	-2.19157
H	5.42674	5.87103	-3.62444
H	1.53006	-8.72787	0.37956
H	2.00937	-7.39035	-0.67539
H	2.25049	-7.27931	1.08133
H	-1.36778	-7.05023	2.0665
H	-0.24372	-8.41544	2.12762
H	0.28609	-6.8137	2.64622
H	-0.97383	-8.74658	-0.21589
H	-1.83431	-7.21625	-0.35231
H	0.1257	-9.75911	-2.05976
H	0.51488	-8.87095	-3.53288
H	1.43748	-8.57605	-2.06267
H	-0.73733	-5.50548	-2.29266
H	0.92261	-6.06513	-2.10931

H	0.07356	-6.31034	-3.63612
H	-2.65385	-7.1962	-2.55649
H	-1.78497	-8.01936	-3.8454
H	-2.33269	-8.93151	-2.44043
H	-1.20101	6.28734	3.92081
H	-1.62151	7.97969	3.63173
H	-2.50481	6.69715	2.80587
H	-0.29851	8.00876	0.03485
H	-1.97329	7.66017	0.47944
H	-1.1216	9.00954	1.23773
H	1.59977	7.71506	1.7521
H	0.68461	8.63423	2.95278
H	1.24266	7.00983	3.34509
H	-5.2049	-8.6952	1.72567
H	-6.1023	-7.53509	0.74174
H	-4.34922	-7.63938	0.59985
H	-3.78995	-7.92096	3.67802
H	-2.83766	-6.95444	2.54471
H	-3.54196	-6.20176	3.99508
H	-7.14785	-6.48025	2.76461
H	-6.36508	-7.77785	3.65891
H	-7.89864	-5.29943	6.07085
H	-8.43304	-5.36569	4.39638
H	-8.16564	-6.86219	5.30124
H	-5.63744	-6.00493	6.74938
H	-5.70147	-7.56315	5.92482
H	-4.39084	-6.42701	5.57984
H	-5.94832	-3.82543	5.37225
H	-4.80082	-4.35726	4.14517
H	-6.44096	-3.91273	3.68151
H	-4.45284	6.36832	-3.95625
H	-6.174	6.5744	-3.64728
H	-5.14557	7.99008	-3.88275
H	-2.73705	7.22501	-2.30086
H	-3.56273	8.76699	-2.08137
H	-3.2152	7.75549	-0.67291
H	-6.97366	7.33005	-1.41538
H	-6.11363	8.82086	-1.78268

H	-7.37876	8.95026	1.76721
H	-7.70942	9.50808	0.12888
H	-8.09366	7.84685	0.59819
H	-5.60195	7.22109	2.20858
H	-6.18407	6.12826	0.95135
H	-4.50998	6.67584	0.93602
H	-5.03938	9.70708	1.58867
H	-3.93209	9.07432	0.37387
H	-5.18811	10.22768	-0.09037
H	-10.0762	-5.72151	-0.4798
H	-11.71312	-5.7069	-1.13039
H	-10.3938	-4.96513	-2.04327
H	-10.74999	-4.52904	1.69062
H	-11.7042	-3.04824	1.62956
H	-12.43419	-4.58938	1.16454
H	-12.50349	-2.11906	-0.63057
H	-11.92345	-2.84251	-2.14188
H	-13.10746	-3.69005	-1.14614
H	-12.35266	1.60395	-2.32665
H	-12.44496	2.27194	-0.68635
H	-13.25446	3.08533	-2.02572
H	-10.87997	2.74566	-4.0993
H	-11.87698	4.18924	-3.88373
H	-10.11976	4.29742	-3.75296
H	-12.15243	5.20164	-1.44616
H	-11.19198	4.50258	-0.13732
H	-10.40064	5.3861	-1.4455

Table S7. Cartesian coordinates of the transition state (TS1) for **HBPO**.

	X	Y	Z
C	9.9083	-2.44929	0.61277
C	9.85321	-1.07968	0.86055
C	8.63948	-0.36588	0.78629
C	7.44799	-1.06035	0.46946
C	7.50414	-2.4479	0.15882
C	8.72582	-3.11842	0.24294
C	8.59324	1.0843	1.00333
C	9.76019	1.81528	1.30456

C	9.73027	3.19828	1.47006
C	8.50889	3.87409	1.29042
C	7.32985	3.18698	0.99223
C	7.35993	1.76918	0.8823
C	5.1313	-5.0373	-1.29915
C	6.31037	-4.40852	-0.83367
C	6.27489	-3.14434	-0.25601
C	5.0167	-2.48834	-0.09409
C	3.81746	-3.19351	-0.36452
C	3.90921	-4.42896	-1.05186
C	4.9658	-1.08756	0.24942
C	6.17764	-0.35898	0.46046
C	6.13419	1.02771	0.65198
C	4.87986	1.71513	0.61242
C	3.68209	0.99274	0.45625
C	3.72499	-0.4263	0.2929
C	4.84688	3.15711	0.66542
C	6.06087	3.90256	0.77629
C	6.02478	5.28793	0.65562
C	4.81832	5.97064	0.37762
C	3.64255	5.23752	0.30031
C	3.61133	3.84165	0.53721
C	0.15637	-5.50373	0.34116
C	1.33575	-4.75639	0.34163
C	1.31736	-3.35941	0.17866
C	0.0655	-2.68376	0.24854
C	-1.1497	-3.42831	0.34664
C	-1.07126	-4.82596	0.28533
C	0.02943	-1.24218	0.23171
C	1.23724	-0.49985	0.27245
C	1.19725	0.91201	0.3461
C	-0.05044	1.57901	0.28102
C	-1.25388	0.83924	0.15163
C	-1.21871	-0.57074	0.1829
C	-0.11246	3.00941	0.45436
C	1.06668	3.73082	0.78743
C	0.92052	5.01361	1.34513
C	-0.3224	5.64562	1.41867

C	-1.45561	4.9765	0.92995
C	-1.37108	3.67719	0.41469
C	-4.94713	-5.26687	1.70743
C	-3.74631	-4.60245	1.49238
C	-3.6845	-3.37853	0.7848
C	-4.9079	-2.73951	0.4536
C	-6.14033	-3.44564	0.59765
C	-6.13679	-4.70191	1.19505
C	-4.91195	-1.35147	0.05943
C	-3.70223	-0.63518	0.0428
C	-3.72195	0.77717	-0.16787
C	-4.93442	1.43262	-0.44964
C	-6.15224	0.68544	-0.51461
C	-6.14456	-0.68757	-0.23352
C	-4.9379	2.85436	-0.70008
C	-3.73759	3.60028	-0.5746
C	-3.72478	4.9456	-1.01516
C	-4.88335	5.58832	-1.42878
C	-6.09467	4.85925	-1.44691
C	-6.13892	3.51014	-1.10938
C	-8.58729	-3.53059	0.09065
C	-7.39051	-2.81292	0.14395
C	-7.38426	-1.44112	-0.23469
C	-8.59673	-0.81143	-0.60397
C	-9.78405	-1.5711	-0.63958
C	-9.79194	-2.92557	-0.31495
C	-8.59931	0.61884	-0.93135
C	-7.39046	1.35328	-0.86969
C	-7.39553	2.74371	-1.17372
C	-8.59823	3.35914	-1.52891
C	-9.80665	2.64026	-1.59112
C	-9.78981	1.27873	-1.29883
C	2.54403	-2.58067	-0.00027
C	2.5049	-1.20297	0.19161
C	2.41549	1.69765	0.46854
C	2.36708	3.08263	0.59823
C	-2.42572	-2.71422	0.45522
C	-2.4498	-1.34265	0.22544

C	-2.50735	1.55211	-0.02996
C	-2.54151	2.94076	-0.06909
C	11.21915	-3.24021	0.68601
C	12.3333	-2.45298	1.40178
C	10.98964	-4.554	1.46005
C	11.66472	-3.54642	-0.75774
C	10.99181	4.00681	1.79249
C	12.1362	3.11269	2.30673
C	10.67816	5.05288	2.88117
C	11.44102	4.7101	0.49632
C	5.26547	-6.37301	-2.0548
C	5.89776	-7.37631	-1.07303
C	3.88781	-6.91673	-2.48272
C	6.1133	-6.09843	-3.33041
C	6.98255	-7.22033	-3.96135
C	6.25893	-8.57488	-4.03196
C	7.32175	-6.77964	-5.40747
C	8.309	-7.37539	-3.18881
C	4.84781	7.49435	0.21996
C	3.46976	8.08192	-0.1233
C	5.25992	8.06657	1.60279
C	5.91564	7.93993	-0.81467
C	5.747	7.59951	-2.32271
C	4.67377	8.48584	-2.98476
C	7.10539	7.90394	-3.00456
C	5.40439	6.1225	-2.55934
C	0.16	-7.0324	0.46374
C	1.56402	-7.63527	0.30498
C	-0.31909	-7.34719	1.90494
C	-0.8431	-7.68954	-0.52177
C	-0.56587	-7.67517	-2.05142
C	0.43287	-8.78287	-2.44466
C	-0.04739	-6.31862	-2.54845
C	-1.91136	-7.9745	-2.75907
C	-0.48938	7.0555	1.99809
C	-1.51099	7.00296	3.15055
C	-0.99916	7.98369	0.87612
C	0.83254	7.63046	2.54073

C	-5.01388	-6.61808	2.42797
C	-5.18028	-7.68263	1.31228
C	-3.72496	-6.9375	3.19942
C	-6.24618	-6.71516	3.36762
C	-6.30677	-5.86477	4.66891
C	-7.78551	-5.85127	5.13213
C	-5.46377	-6.50474	5.79085
C	-5.84993	-4.41538	4.45547
C	-4.88816	7.04248	-1.91238
C	-5.18632	6.99341	-3.43374
C	-3.52563	7.72933	-1.73121
C	-6.01764	7.86303	-1.23296
C	-5.94412	8.2003	0.28292
C	-7.36248	8.65217	0.7141
C	-5.53751	6.99326	1.14004
C	-4.97297	9.36788	0.54988
C	-11.07451	-3.76426	-0.34311
C	-10.79771	-5.11407	-1.03547
C	-11.51502	-3.99739	1.11574
C	-12.21179	-3.06038	-1.10767
C	-11.08657	3.37209	-2.01007
C	-12.3509	2.53239	-1.74619
C	-10.98555	3.66943	-3.51932
C	-11.2128	4.68872	-1.217
H	10.76223	-0.53022	1.11531
H	8.76727	-4.18654	0.01968
H	10.70216	1.27088	1.40324
H	8.48445	4.96178	1.38822
H	7.26703	-4.91852	-0.9641
H	2.98608	-4.87921	-1.42229
H	6.94798	5.86394	0.74653
H	2.71262	5.73596	0.0194
H	2.29343	-5.2613	0.48142
H	-1.98446	-5.42049	0.18786
H	1.80121	5.50636	1.76218
H	-2.42913	5.46639	0.99823
H	-2.82039	-5.00967	1.90445
H	-7.07374	-5.25257	1.31108

H	-2.76688	5.46778	-1.05414
H	-7.01169	5.37232	-1.74787
H	-8.59123	-4.5867	0.36921
H	-10.71113	-1.06997	-0.92762
H	-8.60351	4.42609	-1.76507
H	-10.71058	0.69333	-1.35248
H	13.24987	-3.04854	1.47505
H	12.59187	-1.53316	0.86721
H	12.03969	-2.18236	2.422
H	11.92454	-5.1093	1.58638
H	10.58352	-4.35776	2.45884
H	10.28709	-5.21757	0.94571
H	12.60428	-4.10738	-0.77402
H	10.91803	-4.13926	-1.29573
H	11.81889	-2.62442	-1.32935
H	11.83906	2.56102	3.2055
H	12.45754	2.386	1.5535
H	13.01531	3.71153	2.56868
H	10.26675	4.57717	3.77867
H	11.5794	5.59799	3.17961
H	9.94985	5.79582	2.54062
H	11.65985	3.98364	-0.29421
H	10.66783	5.38298	0.11163
H	12.34488	5.30533	0.65871
H	5.37289	-7.37885	-0.11244
H	5.86183	-8.39922	-1.47697
H	6.95318	-7.14571	-0.88039
H	3.24157	-7.12494	-1.61449
H	3.35587	-6.21412	-3.13405
H	3.98837	-7.85282	-3.04251
H	6.78961	-5.23918	-3.14408
H	5.41446	-5.72417	-4.10731
H	5.28759	-8.48105	-4.52853
H	6.84471	-9.31248	-4.5889
H	6.08204	-8.98696	-3.02837
H	6.42363	-6.71183	-6.02863
H	7.81198	-5.80182	-5.42851
H	7.9974	-7.49209	-5.89041

H	8.91301	-6.46475	-3.24478
H	8.13781	-7.58928	-2.1276
H	8.91243	-8.19251	-3.59563
H	3.09973	7.70406	-1.08743
H	2.71613	7.85165	0.64852
H	3.52093	9.17313	-0.20734
H	5.24181	9.16047	1.60352
H	4.57594	7.72583	2.38784
H	6.26673	7.75472	1.89533
H	6.89227	7.52876	-0.48644
H	6.02242	9.04026	-0.733
H	4.846	9.54722	-2.78864
H	4.66264	8.3481	-4.07074
H	3.66945	8.23654	-2.61877
H	7.9034	7.26666	-2.61264
H	7.0533	7.72472	-4.08319
H	7.40563	8.94537	-2.8623
H	6.11042	5.45548	-2.05198
H	4.40395	5.87034	-2.19157
H	5.42674	5.87103	-3.62444
H	1.53006	-8.72787	0.37956
H	2.00937	-7.39035	-0.67539
H	2.25049	-7.27931	1.08133
H	-1.36778	-7.05023	2.0665
H	-0.24372	-8.41544	2.12762
H	0.28609	-6.8137	2.64622
H	-0.97383	-8.74658	-0.21589
H	-1.83431	-7.21625	-0.35231
H	0.1257	-9.75911	-2.05976
H	0.51488	-8.87095	-3.53288
H	1.43748	-8.57605	-2.06267
H	-0.73733	-5.50548	-2.29266
H	0.92261	-6.06513	-2.10931
H	0.07356	-6.31034	-3.63612
H	-2.65385	-7.1962	-2.55649
H	-1.78497	-8.01936	-3.8454
H	-2.33269	-8.93151	-2.44043
H	-1.20101	6.28734	3.92081

H	-1.62151	7.97969	3.63173
H	-2.50481	6.69715	2.80587
H	-0.29851	8.00876	0.03485
H	-1.97329	7.66017	0.47944
H	-1.1216	9.00954	1.23773
H	1.59977	7.71506	1.7521
H	0.68461	8.63423	2.95278
H	1.24266	7.00983	3.34509
H	-5.2049	-8.6952	1.72567
H	-6.1023	-7.53509	0.74174
H	-4.34922	-7.63938	0.59985
H	-3.78995	-7.92096	3.67802
H	-2.83766	-6.95444	2.54471
H	-3.54196	-6.20176	3.99508
H	-7.14785	-6.48025	2.76461
H	-6.36508	-7.77785	3.65891
H	-7.89864	-5.29943	6.07085
H	-8.43304	-5.36569	4.39638
H	-8.16564	-6.86219	5.30124
H	-5.63744	-6.00493	6.74938
H	-5.70147	-7.56315	5.92482
H	-4.39084	-6.42701	5.57984
H	-5.94832	-3.82543	5.37225
H	-4.80082	-4.35726	4.14517
H	-6.44096	-3.91273	3.68151
H	-4.45284	6.36832	-3.95625
H	-6.174	6.5744	-3.64728
H	-5.14557	7.99008	-3.88275
H	-2.73705	7.22501	-2.30086
H	-3.56273	8.76699	-2.08137
H	-3.2152	7.75549	-0.67291
H	-6.97366	7.33005	-1.41538
H	-6.11363	8.82086	-1.78268
H	-7.37876	8.95026	1.76721
H	-7.70942	9.50808	0.12888
H	-8.09366	7.84685	0.59819
H	-5.60195	7.22109	2.20858
H	-6.18407	6.12826	0.95135

H	-4.50998	6.67584	0.93602
H	-5.03938	9.70708	1.58867
H	-3.93209	9.07432	0.37387
H	-5.18811	10.22768	-0.09037
H	-10.0762	-5.72151	-0.4798
H	-11.71312	-5.7069	-1.13039
H	-10.3938	-4.96513	-2.04327
H	-10.74999	-4.52904	1.69062
H	-11.7042	-3.04824	1.62956
H	-12.43419	-4.58938	1.16454
H	-12.50349	-2.11906	-0.63057
H	-11.92345	-2.84251	-2.14188
H	-13.10746	-3.69005	-1.14614
H	-12.35266	1.60395	-2.32665
H	-12.44496	2.27194	-0.68635
H	-13.25446	3.08533	-2.02572
H	-10.87997	2.74566	-4.0993
H	-11.87698	4.18924	-3.88373
H	-10.11976	4.29742	-3.75296
H	-12.15243	5.20164	-1.44616
H	-11.19198	4.50258	-0.13732
H	-10.40064	5.3861	-1.4455

Table S8. Selected top five frequencies of the transition state (TS1) for **HBPO**.

Mode	Frequency	Infrared
1	-5.93	0.0005
2	6.05	0.0061
3	8.42	0.0067
4	8.86	0.0032
5	9.00	0.0082

Table S9. Cartesian coordinates of the transition state (TS2) of **HBPO**.

	X	Y	Z
C	-9.787042	-2.81354	-0.01685
C	-9.798618	-1.482675	-0.428963
C	-8.615327	-0.718531	-0.48436
C	-7.385539	-1.322634	-0.129778
C	-7.372424	-2.66294	0.347483

C	-8.566517	-3.385773	0.388853
C	-8.638606	0.695948	-0.873201
C	-9.843961	1.34008	-1.216726
C	-9.87823	2.694535	-1.543011
C	-8.682953	3.434509	-1.483316
C	-7.467083	2.833549	-1.148032
C	-7.432583	1.437991	-0.877057
C	-4.84637	-4.964615	2.014192
C	-6.064921	-4.442624	1.521722
C	-6.101959	-3.255447	0.79771
C	-4.87955	-2.574692	0.513365
C	-3.642767	-3.193942	0.82381
C	-3.658885	-4.342687	1.650451
C	-4.898611	-1.225766	0.000535
C	-6.14666	-0.576789	-0.251849
C	-6.168974	0.777387	-0.609369
C	-4.944996	1.51187	-0.702659
C	-3.712309	0.860856	-0.509244
C	-3.689257	-0.526454	-0.16998
C	-4.976836	2.938133	-0.923373
C	-6.224142	3.61768	-1.055571
C	-6.249167	5.011357	-1.064779
C	-5.068101	5.761736	-0.904447
C	-3.850976	5.087199	-0.84113
C	-3.765911	3.680256	-0.932147
C	0.089124	-5.420874	0.346223
C	-1.117254	-4.724939	0.240739
C	-1.153623	-3.320625	0.236498
C	0.069435	-2.610435	0.068163
C	1.311847	-3.314	0.061966
C	1.287443	-4.694903	0.316177
C	0.042042	-1.179754	-0.111932
C	-1.198951	-0.498814	-0.223011
C	-1.223131	0.889289	-0.487021
C	-0.00306	1.606114	-0.560447
C	1.234174	0.940669	-0.364792
C	1.260569	-0.460014	-0.18773
C	-0.010122	2.997914	-0.938705

C	-1.233312	3.631056	-1.303107
C	-1.164289	4.833191	-2.02314
C	0.056444	5.479191	-2.268879
C	1.234669	4.906697	-1.783362
C	1.219766	3.700733	-1.065443
C	5.182439	-5.181384	-1.041091
C	3.952891	-4.537585	-0.911645
C	3.840548	-3.22528	-0.404109
C	5.040164	-2.491998	-0.197464
C	6.299052	-3.156594	-0.263698
C	6.349757	-4.490152	-0.670478
C	4.986696	-1.060445	-0.018199
C	3.746662	-0.401324	-0.089298
C	3.708979	1.025827	-0.100535
C	4.896142	1.766933	0.050029
C	6.144666	1.089975	0.211558
C	6.192357	-0.309683	0.146351
C	4.845813	3.210123	0.058515
C	3.615949	3.877886	-0.173404
C	3.562482	5.28115	0.000838
C	4.702651	6.031852	0.251844
C	5.93774	5.359623	0.393614
C	6.024579	3.971885	0.323072
C	8.754198	-3.063888	0.204092
C	7.526131	-2.412278	0.065845
C	7.463972	-1.001686	0.238929
C	8.651807	-0.274471	0.491968
C	9.870784	-0.970147	0.620654
C	9.934527	-2.356686	0.498137
C	8.595299	1.187501	0.60324
C	7.356823	1.854232	0.440135
C	7.307709	3.274358	0.515674
C	8.483908	3.984619	0.769569
C	9.716592	3.331843	0.954272
C	9.757586	1.942727	0.859097
C	-2.409113	-2.577233	0.346774
C	-2.43381	-1.234816	-0.014988
C	-2.477752	1.606724	-0.662193

C	-2.494501	2.964415	-0.965837
C	2.555673	-2.570709	-0.161473
C	2.522904	-1.180032	-0.140278
C	2.460022	1.720033	-0.331758
C	2.436683	3.096348	-0.520733
C	-11.06605	-3.655259	0.05326
C	-12.222815	-3.018168	-0.74015
C	-11.471235	-3.769096	1.536295
C	-10.801	-5.056828	-0.532555
C	-11.182781	3.408342	-1.914244
C	-11.630943	4.230226	-0.689484
C	-12.297286	2.414697	-2.293929
C	-10.944906	4.340742	-3.119151
C	-4.874795	-6.224272	2.886883
C	-3.522851	-6.506372	3.559604
C	-5.182279	-7.401906	1.925241
C	-6.008213	-6.165928	3.946654
C	-5.92021	-5.165304	5.134492
C	-7.339307	-5.070214	5.750123
C	-4.961936	-5.679674	6.227818
C	-5.483867	-3.760486	4.697498
C	-5.089213	7.293764	-0.855445
C	-4.552114	7.771463	-2.229549
C	-6.508524	7.858835	-0.686557
C	-4.144285	7.84682	0.244942
C	-4.4925	7.650458	1.748115
C	-4.960825	6.224245	2.06765
C	-3.19872	7.93469	2.551797
C	-5.568178	8.655712	2.207873
C	0.122314	-6.959422	0.459826
C	-1.261871	-7.502027	0.847659
C	1.111751	-7.344369	1.577014
C	0.584388	-7.467154	-0.934668
C	0.521231	-8.972261	-1.314735
C	1.199347	-9.115361	-2.700901
C	1.278711	-9.856966	-0.310603
C	-0.927379	-9.478626	-1.4542
C	0.064574	6.794293	-3.057282

C	-0.404956	6.501105	-4.494984
C	-0.893176	7.792252	-2.375346
C	1.464475	7.436712	-3.111865
C	5.224151	-6.615047	-1.604388
C	6.633023	-7.219259	-1.501015
C	4.2724	-7.477054	-0.74708
C	4.732219	-6.508037	-3.074992
C	5.046829	-7.636271	-4.096413
C	4.776031	-9.034895	-3.51706
C	4.097505	-7.428248	-5.303214
C	6.507142	-7.541616	-4.595873
C	4.673262	7.561758	0.329115
C	3.253979	8.135281	0.186662
C	5.498618	8.073528	-0.881832
C	5.357233	8.081275	1.621848
C	4.724839	7.809806	3.016406
C	5.796881	8.1793	4.073337
C	3.490967	8.700821	3.259425
C	4.336565	6.338036	3.211687
C	11.253854	-3.125916	0.628301
C	12.35647	-2.27697	1.28941
C	11.704453	-3.525625	-0.790736
C	11.040554	-4.386108	1.49097
C	10.972472	4.170941	1.214841
C	11.359557	4.860246	-0.108619
C	12.155348	3.311925	1.700805
C	10.674287	5.228949	2.29648
H	-10.738327	-1.004499	-0.714506
H	-8.555187	-4.419216	0.741193
H	-10.764551	0.751959	-1.218647
H	-8.707837	4.503754	-1.705809
H	-6.994426	-4.96756	1.753517
H	-2.707979	-4.717288	2.035523
H	-7.205457	5.52558	-1.175635
H	-2.937978	5.666957	-0.675774
H	-2.054657	-5.279469	0.153518
H	2.224648	-5.227188	0.495594
H	-2.078245	5.263885	-2.437523

H	2.196598	5.376536	-1.998363
H	3.048205	-5.052667	-1.242819
H	7.319125	-4.988244	-0.724938
H	2.588094	5.771929	-0.038302
H	6.838687	5.948103	0.582358
H	8.800856	-4.148664	0.082478
H	10.77823	-0.394616	0.817899
H	8.447645	5.075366	0.827324
H	10.704415	1.411378	0.980181
H	-12.509444	-2.042993	-0.333231
H	-11.956772	-2.881432	-1.794136
H	-13.115769	-3.652067	-0.710002
H	-11.65359	-2.781696	1.974913
H	-12.38568	-4.358426	1.655291
H	-10.689799	-4.248992	2.134257
H	-10.064959	-5.615829	0.054136
H	-11.716463	-5.656613	-0.557332
H	-10.420952	-4.990402	-1.558312
H	-10.879317	4.973098	-0.403338
H	-12.56434	4.765628	-0.888805
H	-11.797184	3.586591	0.18151
H	-13.208739	2.942717	-2.594744
H	-11.996709	1.779724	-3.134695
H	-12.567141	1.763075	-1.456531
H	-10.243023	5.147151	-2.883425
H	-10.534894	3.787325	-3.971592
H	-11.87738	4.810752	-3.447827
H	-2.712142	-6.644736	2.825051
H	-3.232069	-5.689303	4.234523
H	-3.569838	-7.420186	4.162152
H	-4.428556	-7.468874	1.133082
H	-5.187458	-8.359301	2.454562
H	-6.154869	-7.288324	1.43724
H	-6.120568	-7.181676	4.375585
H	-6.960369	-5.97319	3.410366
H	-8.061458	-4.66549	5.035157
H	-7.703021	-6.046539	6.080975
H	-7.346516	-4.407477	6.62142

H	-3.918076	-5.644745	5.893884
H	-5.028342	-5.065764	7.131918
H	-5.18647	-6.710509	6.513365
H	-6.145296	-3.351359	3.925458
H	-5.493185	-3.058774	5.537583
H	-4.468936	-3.755962	4.285113
H	-3.497736	7.488117	-2.379793
H	-5.12638	7.331889	-3.051912
H	-4.616214	8.858857	-2.328717
H	-7.172607	7.552569	-1.500869
H	-6.957082	7.536048	0.263336
H	-6.49235	8.954792	-0.677376
H	-4.02242	8.934217	0.070311
H	-3.136914	7.411779	0.071562
H	-5.894028	5.973264	1.552411
H	-4.217421	5.476956	1.766633
H	-5.136922	6.09153	3.139781
H	-2.405729	7.223104	2.301634
H	-2.818281	8.942083	2.36433
H	-3.376259	7.848061	3.628498
H	-6.546128	8.423784	1.770326
H	-5.690355	8.632591	3.29561
H	-5.311906	9.680764	1.928037
H	-1.612797	-7.082904	1.805601
H	-2.01619	-7.270005	0.086301
H	-1.241513	-8.594509	0.957591
H	0.876589	-6.821464	2.510598
H	1.07465	-8.422621	1.782381
H	2.147195	-7.10136	1.317192
H	0.004461	-6.910812	-1.700179
H	1.644292	-7.139658	-1.069119
H	1.139353	-10.145753	-3.064794
H	0.721467	-8.478721	-3.450961
H	2.268324	-8.848971	-2.671386
H	1.2304	-10.912727	-0.594193
H	2.335894	-9.580289	-0.249071
H	0.859861	-9.765692	0.700174
H	-0.963064	-10.449818	-1.958198

H	-1.405977	-9.608999	-0.476297
H	-1.541736	-8.785113	-2.035945
H	0.248133	5.767797	-4.982332
H	-1.420584	6.092172	-4.518774
H	-0.401746	7.406729	-5.109269
H	-1.943238	7.458356	-2.420035
H	-0.637247	7.929598	-1.319721
H	-0.850963	8.77417	-2.857378
H	2.184453	6.794893	-3.631923
H	1.438296	8.389492	-3.651069
H	1.862963	7.643792	-2.10484
H	6.635302	-8.258489	-1.862681
H	7.356825	-6.668574	-2.112293
H	6.99102	-7.235416	-0.466765
H	4.394613	-8.543898	-0.958046
H	4.467664	-7.332992	0.321027
H	3.211512	-7.233914	-0.928168
H	5.126789	-5.562034	-3.502584
H	3.632352	-6.367983	-3.047867
H	4.892048	-9.812595	-4.277497
H	5.466501	-9.266248	-2.695428
H	3.749399	-9.107878	-3.122124
H	4.221686	-6.435627	-5.747058
H	4.289096	-8.165466	-6.088986
H	3.047096	-7.53031	-5.01396
H	7.061511	-6.740961	-4.094412
H	7.061759	-8.469137	-4.428101
H	6.561735	-7.333579	-5.669146
H	2.60308	7.806513	1.010129
H	3.271919	9.230455	0.213264
H	2.778937	7.842144	-0.763699
H	6.547272	7.767198	-0.826199
H	5.097866	7.681391	-1.82304
H	5.478307	9.165473	-0.945948
H	5.476279	9.178676	1.519763
H	6.390391	7.677897	1.639008
H	5.408423	8.0522	5.088872
H	6.682186	7.542731	3.987474

H	6.119246	9.219382	3.976762
H	3.136144	8.611663	4.291217
H	3.709222	9.755926	3.075604
H	2.656695	8.413122	2.606425
H	4.027331	6.138002	4.242523
H	3.50225	6.045674	2.564955
H	5.170208	5.664229	2.984621
H	13.279397	-2.855162	1.407781
H	12.606819	-1.394553	0.691603
H	12.055482	-1.937493	2.286643
H	10.963067	-4.159612	-1.287755
H	11.851752	-2.643482	-1.423763
H	12.648581	-4.078551	-0.768035
H	10.348541	-5.093381	1.02252
H	11.98273	-4.918416	1.65639
H	10.629803	-4.127067	2.473408
H	12.25715	5.474979	0.009928
H	11.562942	4.124264	-0.894542
H	10.560225	5.511933	-0.475997
H	11.908123	2.775626	2.623559
H	12.460564	2.575219	0.950565
H	13.031728	3.934417	1.91222
H	10.306446	4.760143	3.216188
H	11.573042	5.799131	2.552663
H	9.917541	5.949313	1.970017

Table S10. Selected top five frequencies of the transition state (TS1) for **HBPO**.

Mode	Frequency	Infrared
1	-147.48	0.0271
2	5.96	0.0069
3	8.04	0.0018
4	9.04	0.0035
5	9.29	0.0074

Table S11. Cartesian coordinates of dibenzo-*peri*-octacene.

	X	Y	Z
C	-1.20007	5.00300	-0.00041
C	-1.22777	3.60125	-0.00024

C	0.00000	2.88126	-0.00022
C	1.22777	3.60125	-0.00029
C	1.20007	5.00300	-0.00047
C	0.00000	5.69766	-0.00053
C	0.00000	1.43051	-0.00015
C	-1.23222	0.71836	-0.00013
C	-1.23222	-0.71837	-0.00014
C	0.00000	-1.43051	-0.00015
C	1.23223	-0.71837	-0.00013
C	1.23223	0.71836	-0.00014
C	0.00000	-2.88126	-0.00022
C	-1.22777	-3.60126	-0.00028
C	-1.20007	-5.00301	-0.00045
C	0.00000	-5.69767	-0.00053
C	1.20007	-5.00300	-0.00042
C	1.22777	-3.60126	-0.00025
C	-2.49714	2.87027	-0.00010
C	2.49714	2.87027	-0.00019
C	-2.49714	-2.87027	-0.00019
C	2.49714	-2.87027	-0.00011
C	2.47784	1.42794	-0.00013
C	3.70386	0.71754	-0.00006
C	4.95085	1.41338	-0.00001
C	4.95142	2.84831	-0.00005
C	3.70914	3.52808	-0.00013
C	6.18423	0.71911	0.00007
C	7.41406	1.43054	0.00012
C	7.39676	2.86894	0.00007
C	6.16357	3.54022	-0.00001
C	-3.70914	3.52808	0.00001
C	-4.95142	2.84831	0.00010
C	-4.95085	1.41338	0.00006
C	-3.70386	0.71754	-0.00003
C	-2.47784	1.42794	-0.00009
C	-6.16357	3.54022	0.00020
C	-7.39676	2.86894	0.00026
C	-7.41406	1.43054	0.00021
C	-6.18423	0.71911	0.00012

C	-2.47784	-1.42794	-0.00013
C	-3.70386	-0.71754	-0.00006
C	-4.95085	-1.41338	-0.00001
C	-4.95142	-2.84831	-0.00006
C	-3.70915	-3.52808	-0.00014
C	-6.18423	-0.71911	0.00007
C	-7.41406	-1.43054	0.00012
C	-7.39676	-2.86894	0.00007
C	-6.16357	-3.54022	-0.00002
C	3.70914	-3.52808	0.00002
C	4.95142	-2.84831	0.00010
C	4.95085	-1.41338	0.00006
C	3.70386	-0.71754	-0.00003
C	2.47784	-1.42794	-0.00010
C	6.16357	-3.54022	0.00021
C	7.39676	-2.86894	0.00026
C	7.41406	-1.43054	0.00021
C	6.18423	-0.71911	0.00011
C	-8.62752	3.57827	0.00037
C	-9.82099	2.89508	0.00042
C	-9.84099	1.48765	0.00037
C	-8.66851	0.73587	0.00026
C	-8.66851	-0.73587	0.00020
C	-9.84099	-1.48765	0.00023
C	-9.82099	-2.89507	0.00018
C	-8.62752	-3.57827	0.00010
C	8.66851	0.73587	0.00020
C	9.84099	1.48765	0.00023
C	9.82099	2.89508	0.00018
C	8.62752	3.57827	0.00011
C	8.62752	-3.57827	0.00036
C	9.82099	-2.89507	0.00041
C	9.84099	-1.48765	0.00036
C	8.66851	-0.73587	0.00026
H	-2.12252	5.56900	-0.00048
H	2.12252	5.56900	-0.00057
H	0.00000	6.78338	-0.00068
H	-2.12252	-5.56900	-0.00054

H	0.00000	-6.78339	-0.00068
H	2.12252	-5.56900	-0.00050
H	3.75058	4.61021	-0.00014
H	6.15426	4.62725	-0.00004
H	-3.75058	4.61021	0.00005
H	-6.15426	4.62725	0.00024
H	-3.75058	-4.61021	-0.00016
H	-6.15426	-4.62725	-0.00005
H	3.75058	-4.61021	0.00007
H	6.15426	-4.62725	0.00024
H	-8.60677	4.66436	0.00040
H	-10.76143	3.43813	0.00050
H	-10.80498	0.99258	0.00041
H	-10.80499	-0.99258	0.00029
H	-10.76143	-3.43813	0.00021
H	-8.60678	-4.66436	0.00006
H	10.80498	0.99258	0.00028
H	10.76143	3.43813	0.00021
H	8.60677	4.66436	0.00007
H	8.60678	-4.66436	0.00040
H	10.76143	-3.43813	0.00049
H	10.80499	-0.99258	0.00041

Table S12. Cartesian coordinates of *peri*-octacene.

	X	Y	Z
C	1.22909	3.55198	-0.00001
C	0.00000	2.87313	0.00000
C	-1.22909	3.55198	0.00001
C	0.00000	1.42212	0.00001
C	1.22885	0.71736	0.00000
C	1.22885	-0.71736	0.00001
C	0.00000	-1.42212	0.00000
C	-1.22885	-0.71736	0.00000
C	-1.22885	0.71736	0.00001
C	0.00000	-2.87313	-0.00001
C	1.22909	-3.55198	0.00001
C	-1.22909	-3.55198	-0.00002
C	2.46264	2.87217	-0.00002

C	-2.46264	2.87217	0.00002
C	2.46264	-2.87217	0.00002
C	-2.46264	-2.87217	-0.00002
C	-2.46523	1.42209	0.00001
C	-3.68832	0.71783	0.00001
C	-4.92994	1.42275	0.00002
C	-4.92186	2.86896	0.00004
C	-3.68761	3.55074	0.00003
C	-6.15531	0.72164	0.00001
C	-7.39094	1.43077	0.00003
C	-7.37431	2.87145	0.00005
C	-6.14643	3.55064	0.00006
C	3.68761	3.55074	-0.00003
C	4.92186	2.86896	-0.00004
C	4.92994	1.42275	-0.00002
C	3.68832	0.71783	-0.00001
C	2.46523	1.42209	0.00000
C	6.14643	3.55064	-0.00006
C	7.37431	2.87145	-0.00006
C	7.39093	1.43077	-0.00003
C	6.15531	0.72164	-0.00001
C	2.46523	-1.42209	0.00001
C	3.68832	-0.71783	0.00001
C	4.92994	-1.42275	0.00002
C	4.92186	-2.86896	0.00003
C	3.68761	-3.55074	0.00003
C	6.15531	-0.72164	0.00001
C	7.39094	-1.43077	0.00003
C	7.37431	-2.87145	0.00006
C	6.14643	-3.55064	0.00006
C	-3.68761	-3.55074	-0.00004
C	-4.92186	-2.86896	-0.00004
C	-4.92994	-1.42275	-0.00001
C	-3.68832	-0.71783	-0.00001
C	-2.46523	-1.42209	-0.00001
C	-6.14643	-3.55064	-0.00006
C	-7.37431	-2.87145	-0.00006
C	-7.39094	-1.43077	-0.00003

C	-6.15531	-0.72164	-0.00001
C	8.60809	3.57701	-0.00009
C	9.80039	2.89137	-0.00009
C	9.81798	1.48491	-0.00006
C	8.64131	0.73570	-0.00002
C	8.64131	-0.73570	0.00002
C	9.81798	-1.48491	0.00006
C	9.80039	-2.89137	0.00009
C	8.60810	-3.57701	0.00009
C	-8.64131	0.73570	0.00002
C	-9.81798	1.48491	0.00005
C	-9.80039	2.89137	0.00008
C	-8.60809	3.57701	0.00008
C	-8.60810	-3.57701	-0.00008
C	-9.80039	-2.89137	-0.00008
C	-9.81798	-1.48491	-0.00005
C	-8.64131	-0.73570	-0.00002
H	1.22957	4.63911	-0.00002
H	-1.22958	4.63911	0.00001
H	1.22958	-4.63911	0.00000
H	-1.22958	-4.63911	-0.00003
H	-3.68952	4.63786	0.00004
H	-6.14614	4.63784	0.00007
H	3.68952	4.63786	-0.00005
H	6.14614	4.63784	-0.00008
H	3.68952	-4.63786	0.00004
H	6.14614	-4.63784	0.00007
H	-3.68952	-4.63786	-0.00006
H	-6.14614	-4.63784	-0.00008
H	8.59008	4.66333	-0.00011
H	10.74180	3.43331	-0.00012
H	10.78044	0.98692	-0.00007
H	10.78044	-0.98692	0.00007
H	10.74180	-3.43331	0.00012
H	8.59008	-4.66333	0.00011
H	-10.78044	0.98692	0.00005
H	-10.74180	3.43331	0.00010
H	-8.59008	4.66333	0.00010

H	-8.59008	-4.66333	-0.00010
H	-10.74180	-3.43331	-0.00011
H	-10.78044	-0.98692	-0.00005

9. References

[1] Sens, R.; Drexhage, K. H. Fluorescence quantum yield of oxazine and carbazine laser dyes. *J. Lumin.* **1981**, *24-25*, 709-712.

[2] Gaussian 09, R. D., M. J. Frisch, G. W. Trucks, H. B. Schlegel, G. E. Scuseria, M. A. Robb, J. R. Cheeseman, G. Scalmani, V. Barone, B. Mennucci, G. A. Petersson, H. Nakatsuji, M. Caricato, X. Li, H. P. Hratchian, A. F. Izmaylov, J. Bloino, G. Zheng, J. L. Sonnenberg, M. Hada, M. Ehara, K. Toyota, R. Fukuda, J. Hasegawa, M. Ishida, T. Nakajima, Y. Honda, O. Kitao, H. Nakai, T. Vreven, J. A. Montgomery, Jr., J. E. Peralta, F. Ogliaro, M. Bearpark, J. J. Heyd, E. Brothers, K. N. Kudin, V. N. Staroverov, T. Keith, R. Kobayashi, J. Normand, K. Raghavachari, A. Rendell, J. C. Burant, S. S. Iyengar, J. Tomasi, M. Cossi, N. Rega, J. M. Millam, M. Klene, J. E. Knox, J. B. Cross, V. Bakken, C. Adamo, J. Jaramillo, R. Gomperts, R. E. Stratmann, O. Yazyev, A. J. Austin, R. Cammi, C. Pomelli, J. W. Ochterski, R. L. Martin, K. Morokuma, V. G. Zakrzewski, G. A. Voth, P. Salvador, J. J. Dannenberg, S. Dapprich, A. D. Daniels, O. Farkas, J. B. Foresman, J. V. Ortiz, J. Cioslowski, and D. J. Fox, Gaussian, Inc., Wallingford CT, 2013.

[3] G. Li, K.-Y. Yoon, X. Zhong, X. Zhu, G. Dong, *Chem. Eur. J.* **2016**, *22*, 9116.

[4] Y. Gu, R. Muñoz-Mármol, S. Wu, Y. Han, Y. Ni, M. A. Díaz-García, J. Casado, J. Wu, *Angew. Chem. Int. Ed.* **2020**, *59*, 8113.

[5] a) A. D. Becke, *J. Chem. Phys.* **1993**, *98*, 5648; b) C. Lee, W. Yang, R. G. Parr, *Phys. Rev. B: Condens. Matter* **1988**, *37*, 785; c) T. Yanai, D. Tew, N. Handy, *Chem. Phys. Lett.* **2004**, *393*, 51.

[6] a) R. Ditchfield, W. J. Hehre, J. A. Pople, *J. Chem. Phys.* **1971**, *54*, 724; b) W. J. Hehre, R. Ditchfield, Pople, J. A. *Chem. Phys.* **1972**, *56*, 2257; c) P. C. Hariharan, J. A. Pople, *Theor. Chim. Acta* **1973**, *28*, 213.

[7] H. Fallah-Bagher-Shaidaei, S. S. Wannere, C. Corminboeuf, R. Puchta, P. v. R. Schleyer, *Org. Lett.* **2006**, *8*, 863.

[8] D. Geuenich, K. Hess, F. Köhler, R. Herges, *Chem. Rev.* **2005**,*105*, 3758.

[9] G. M. Paternò, Q. Chen, X.-Y. Wang, J. Liu, S. G. Motti, A. Petrozza, X. Feng, G. Lanzani, K. Müllen, A. Narita, F. Scotognella, *Angew. Chem. Int. Ed.* **2017**, *56*, 6753.

[10] V. Bonal, R. Munoz-Marmol, F. Gordillo Gamez, M. Morales-Vidal, J. M. Villalvilla, P. G. Boj, J. A. Quintana, Y. Gu, J. Wu, J. Casado, M. A. Diaz-Garcia, *Nat Commun* **2019**, *10*, 3327.

[11] R. Muñoz-Mármol, F. Gordillo, V. Bonal, J. M. Villalvilla, P. G. Boj, J. A. Quintana, A. M. Ross, G. M. Paternò, F. Scotognella, G. Lanzani, A. Derradji, J. C. Sancho-García, Y. Gu, J. Wu, J. Casado, M. A. Díaz-García, *Adv. Funct. Mater.* **2021**, *31*, 2105073.
