# Supplementary material for: Cove‐Edged Hexa‐peri‐hexabenzo‐bis‐peri‐octacene: Molecular Conformations and Amplified Spontaneous Emission
Source: Angew Chem Int Ed Engl. 2022 Mar 2;61(18):e202201088. doi: 10.1002/anie.202201088 (PMC9311809; doi:10.1002/anie.202201088)

checkCIF/PLATON report

Structure factors have been supplied for datablock(s) hbpo

THIS REPORT IS FOR GUIDANCE ONLY. IF USED AS PART OF A REVIEW PROCEDURE FOR PUBLICATION, IT SHOULD NOT REPLACE THE EXPERTISE OF AN EXPERIENCED CRYSTALLOGRAPHIC REFEREE.

No syntax errors found. CIF dictionary Interpreting this report

Datablock: hbpo

Bond precision: C-C = 0.0069 A Wavelength=0.71073

Cell:	a=10.7542(6)	b=14.8759(8)	c=20.7535(14)
	alpha=89.158(5)	beta=84.844(5)	gamma=79.496(5)
Temperature:	120 K		

	Calculated	Reported
Volume	3251.3(3)	3251.3(3)
Space group	P -1	P -1
Hall group	-P 1	-P 1
Moiety formula	C146 H150, 3(C9 H12)	C146 H150, 3(C9 H12)
Sum formula	C173 H186	C173 H186
Mr	2265.22	2265.21
Dx,g cm-3	1.157	1.157
Z	1	1
Mu (mm-1)	0.065	0.065
F000	1224.0	1224.0
F000'	1224.42	
h,k,lmax	14,19,27	14,19,27
Nref	15991	15584
Tmin,Tmax	0.988,0.995	0.975,0.996
Tmin'	0.969	

Correction method= # Reported T Limits: Tmin=0.975 Tmax=0.996
AbsCorr = INTEGRATION

Data completeness= 0.975 Theta(max)= 28.176

R(reflections)= 0.1344(7276) wR2(reflections)= 0.3047(15584)

S = 1.127 Npar= 961

The following ALERTS were generated. Each ALERT has the format
test-name_ALERT_alert-type_alert-level.
Click on the hyperlinks for more details of the test.

Alert level B

```
PLAT910_ALERT_3_B Missing # of FCF Reflection(s) Below Theta(Min).         11 Note
```

Author Response: reflections obscured by beam stop.

Alert level C

```
ABSTY02_ALERT_1_C  An _exptl_absorpt_correction_type has been given without
            a literature citation. This should be contained in the
            _exptl_absorpt_process_details field.
            Absorption correction given as integration
PLAT026_ALERT_3_C Ratio Observed / Unique Reflections (too) Low ..        47% Check
PLAT082_ALERT_2_C High R1 Value ..................................       0.13 Report
PLAT084_ALERT_3_C High wR2 Value (i.e. > 0.25) ...................       0.30 Report
PLAT213_ALERT_2_C Atom C57A            has ADP max/min Ratio .....        3.3 prolat
PLAT213_ALERT_2_C Atom C71A            has ADP max/min Ratio .....        3.8 prolat
PLAT220_ALERT_2_C NonSolvent   Resd 1  C   Ueq(max)/Ueq(min) Range        5.3 Ratio
PLAT222_ALERT_3_C NonSolvent Resd 1  H   Uiso(max)/Uiso(min) Range        6.7 Ratio
PLAT234_ALERT_4_C Large Hirshfeld Difference C56      --C58A     .       0.21 Ang.
PLAT242_ALERT_2_C Low    'MainMol' Ueq as Compared to Neighbors of        C44 Check
PLAT242_ALERT_2_C Low    'MainMol' Ueq as Compared to Neighbors of        C60 Check
PLAT242_ALERT_2_C Low    'MainMol' Ueq as Compared to Neighbors of        C64 Check
PLAT244_ALERT_4_C Low    'Solvent' Ueq as Compared to Neighbors of        C5L Check
PLAT340_ALERT_3_C Low Bond Precision on  C-C Bonds ..............     0.0069 Ang.
PLAT410_ALERT_2_C Short Intra H...H Contact  H4        ..H9       .       1.99 Ang.
                                                 x,y,z  =      1_555 Check
PLAT906_ALERT_3_C Large K Value in the Analysis of Variance ......     42.388 Check
PLAT906_ALERT_3_C Large K Value in the Analysis of Variance ......      5.383 Check
PLAT906_ALERT_3_C Large K Value in the Analysis of Variance ......      2.837 Check
```

Alert level G

```
PLAT002_ALERT_2_G Number of Distance or Angle Restraints on AtSite         11 Note
PLAT003_ALERT_2_G Number of Uiso or Uij Restrained non-H Atoms ...          1 Report
PLAT066_ALERT_1_G Predicted and Reported Tmin&Tmax Range Identical          ? Check
PLAT083_ALERT_2_G SHELXL Second Parameter in WGHT  Unusually Large       7.48 Why ?
PLAT154_ALERT_1_G The s.u.'s on the Cell Angles are Equal ..(Note)      0.005 Degree
PLAT176_ALERT_4_G The CIF-Embedded .res File Contains SADI Records          2 Report
PLAT186_ALERT_4_G The CIF-Embedded .res File Contains ISOR Records          1 Report
PLAT187_ALERT_4_G The CIF-Embedded .res File Contains RIGU Records          3 Report
PLAT300_ALERT_4_G Atom Site Occupancy of C48        Constrained at        0.5 Check
PLAT300_ALERT_4_G Atom Site Occupancy of C49        Constrained at        0.5 Check
PLAT300_ALERT_4_G Atom Site Occupancy of C50        Constrained at        0.5 Check
PLAT300_ALERT_4_G Atom Site Occupancy of C51        Constrained at        0.5 Check
PLAT300_ALERT_4_G Atom Site Occupancy of C53        Constrained at        0.7 Check
PLAT300_ALERT_4_G Atom Site Occupancy of C54        Constrained at        0.7 Check
PLAT300_ALERT_4_G Atom Site Occupancy of C55        Constrained at        0.7 Check
PLAT300_ALERT_4_G Atom Site Occupancy of C57        Constrained at        0.7 Check
```

PLAT300_ALERT_4_G Atom Site Occupancy of C58 Constrained at 0.7 Check
PLAT300_ALERT_4_G Atom Site Occupancy of C61 Constrained at 0.75 Check
PLAT300_ALERT_4_G Atom Site Occupancy of C62 Constrained at 0.75 Check
PLAT300_ALERT_4_G Atom Site Occupancy of C63 Constrained at 0.75 Check
PLAT300_ALERT_4_G Atom Site Occupancy of C69 Constrained at 0.85 Check
PLAT300_ALERT_4_G Atom Site Occupancy of C70 Constrained at 0.85 Check
PLAT300_ALERT_4_G Atom Site Occupancy of C71 Constrained at 0.85 Check
PLAT300_ALERT_4_G Atom Site Occupancy of C74 Constrained at 0.85 Check
PLAT300_ALERT_4_G Atom Site Occupancy of C75 Constrained at 0.85 Check
PLAT300_ALERT_4_G Atom Site Occupancy of C53A Constrained at 0.3 Check
PLAT300_ALERT_4_G Atom Site Occupancy of C54A Constrained at 0.3 Check
PLAT300_ALERT_4_G Atom Site Occupancy of C55A Constrained at 0.3 Check
PLAT300_ALERT_4_G Atom Site Occupancy of C57A Constrained at 0.3 Check
PLAT300_ALERT_4_G Atom Site Occupancy of C58A Constrained at 0.3 Check
PLAT300_ALERT_4_G Atom Site Occupancy of C61A Constrained at 0.25 Check
PLAT300_ALERT_4_G Atom Site Occupancy of C62A Constrained at 0.25 Check
PLAT300_ALERT_4_G Atom Site Occupancy of C63A Constrained at 0.25 Check
PLAT300_ALERT_4_G Atom Site Occupancy of C69A Constrained at 0.15 Check
PLAT300_ALERT_4_G Atom Site Occupancy of C70A Constrained at 0.15 Check
PLAT300_ALERT_4_G Atom Site Occupancy of C71A Constrained at 0.15 Check
PLAT300_ALERT_4_G Atom Site Occupancy of C74A Constrained at 0.15 Check
PLAT300_ALERT_4_G Atom Site Occupancy of C75A Constrained at 0.15 Check
PLAT300_ALERT_4_G Atom Site Occupancy of H47A Constrained at 0.5 Check
PLAT300_ALERT_4_G Atom Site Occupancy of H47B Constrained at 0.5 Check
PLAT300_ALERT_4_G Atom Site Occupancy of H47C Constrained at 0.5 Check
PLAT300_ALERT_4_G Atom Site Occupancy of H47D Constrained at 0.5 Check
PLAT300_ALERT_4_G Atom Site Occupancy of H47E Constrained at 0.5 Check
PLAT300_ALERT_4_G Atom Site Occupancy of H49A Constrained at 0.5 Check
PLAT300_ALERT_4_G Atom Site Occupancy of H49B Constrained at 0.5 Check
PLAT300_ALERT_4_G Atom Site Occupancy of H49C Constrained at 0.5 Check
PLAT300_ALERT_4_G Atom Site Occupancy of H50A Constrained at 0.5 Check
PLAT300_ALERT_4_G Atom Site Occupancy of H50B Constrained at 0.5 Check
PLAT300_ALERT_4_G Atom Site Occupancy of H50C Constrained at 0.5 Check
PLAT300_ALERT_4_G Atom Site Occupancy of H51A Constrained at 0.5 Check
PLAT300_ALERT_4_G Atom Site Occupancy of H51B Constrained at 0.5 Check
PLAT300_ALERT_4_G Atom Site Occupancy of H51C Constrained at 0.5 Check
PLAT300_ALERT_4_G Atom Site Occupancy of H53A Constrained at 0.7 Check
PLAT300_ALERT_4_G Atom Site Occupancy of H53B Constrained at 0.7 Check
PLAT300_ALERT_4_G Atom Site Occupancy of H53C Constrained at 0.7 Check
PLAT300_ALERT_4_G Atom Site Occupancy of H54A Constrained at 0.7 Check
PLAT300_ALERT_4_G Atom Site Occupancy of H54B Constrained at 0.7 Check
PLAT300_ALERT_4_G Atom Site Occupancy of H54C Constrained at 0.7 Check
PLAT300_ALERT_4_G Atom Site Occupancy of H55A Constrained at 0.7 Check
PLAT300_ALERT_4_G Atom Site Occupancy of H55B Constrained at 0.7 Check
PLAT300_ALERT_4_G Atom Site Occupancy of H57A Constrained at 0.7 Check
PLAT300_ALERT_4_G Atom Site Occupancy of H57B Constrained at 0.7 Check
PLAT300_ALERT_4_G Atom Site Occupancy of H57C Constrained at 0.7 Check
PLAT300_ALERT_4_G Atom Site Occupancy of H58A Constrained at 0.7 Check
PLAT300_ALERT_4_G Atom Site Occupancy of H58B Constrained at 0.7 Check
PLAT300_ALERT_4_G Atom Site Occupancy of H58C Constrained at 0.7 Check
PLAT300_ALERT_4_G Atom Site Occupancy of H61A Constrained at 0.75 Check
PLAT300_ALERT_4_G Atom Site Occupancy of H61B Constrained at 0.75 Check
PLAT300_ALERT_4_G Atom Site Occupancy of H61C Constrained at 0.75 Check
PLAT300_ALERT_4_G Atom Site Occupancy of H62A Constrained at 0.75 Check
PLAT300_ALERT_4_G Atom Site Occupancy of H62B Constrained at 0.75 Check
PLAT300_ALERT_4_G Atom Site Occupancy of H62C Constrained at 0.75 Check
PLAT300_ALERT_4_G Atom Site Occupancy of H63A Constrained at 0.75 Check

PLAT300_ALERT_4_G Atom Site Occupancy of H63B Constrained at 0.75 Check
PLAT300_ALERT_4_G Atom Site Occupancy of H63C Constrained at 0.75 Check
PLAT300_ALERT_4_G Atom Site Occupancy of H69A Constrained at 0.85 Check
PLAT300_ALERT_4_G Atom Site Occupancy of H69B Constrained at 0.85 Check
PLAT300_ALERT_4_G Atom Site Occupancy of H69C Constrained at 0.85 Check
PLAT300_ALERT_4_G Atom Site Occupancy of H70A Constrained at 0.85 Check
PLAT300_ALERT_4_G Atom Site Occupancy of H70B Constrained at 0.85 Check
PLAT300_ALERT_4_G Atom Site Occupancy of H70C Constrained at 0.85 Check
PLAT300_ALERT_4_G Atom Site Occupancy of H71A Constrained at 0.85 Check
PLAT300_ALERT_4_G Atom Site Occupancy of H71B Constrained at 0.85 Check
PLAT300_ALERT_4_G Atom Site Occupancy of H74A Constrained at 0.85 Check
PLAT300_ALERT_4_G Atom Site Occupancy of H74B Constrained at 0.85 Check
PLAT300_ALERT_4_G Atom Site Occupancy of H74C Constrained at 0.85 Check
PLAT300_ALERT_4_G Atom Site Occupancy of H75A Constrained at 0.85 Check
PLAT300_ALERT_4_G Atom Site Occupancy of H75B Constrained at 0.85 Check
PLAT300_ALERT_4_G Atom Site Occupancy of H75C Constrained at 0.85 Check
PLAT300_ALERT_4_G Atom Site Occupancy of H53D Constrained at 0.3 Check
PLAT300_ALERT_4_G Atom Site Occupancy of H53E Constrained at 0.3 Check
PLAT300_ALERT_4_G Atom Site Occupancy of H53F Constrained at 0.3 Check
PLAT300_ALERT_4_G Atom Site Occupancy of H54D Constrained at 0.3 Check
PLAT300_ALERT_4_G Atom Site Occupancy of H54E Constrained at 0.3 Check
PLAT300_ALERT_4_G Atom Site Occupancy of H54F Constrained at 0.3 Check
PLAT300_ALERT_4_G Atom Site Occupancy of H55C Constrained at 0.3 Check
PLAT300_ALERT_4_G Atom Site Occupancy of H55D Constrained at 0.3 Check
PLAT300_ALERT_4_G Atom Site Occupancy of H57D Constrained at 0.3 Check
PLAT300_ALERT_4_G Atom Site Occupancy of H57E Constrained at 0.3 Check
PLAT300_ALERT_4_G Atom Site Occupancy of H57F Constrained at 0.3 Check
PLAT300_ALERT_4_G Atom Site Occupancy of H58D Constrained at 0.3 Check
PLAT300_ALERT_4_G Atom Site Occupancy of H58E Constrained at 0.3 Check
PLAT300_ALERT_4_G Atom Site Occupancy of H58F Constrained at 0.3 Check
PLAT300_ALERT_4_G Atom Site Occupancy of H61D Constrained at 0.25 Check
PLAT300_ALERT_4_G Atom Site Occupancy of H61E Constrained at 0.25 Check
PLAT300_ALERT_4_G Atom Site Occupancy of H61F Constrained at 0.25 Check
PLAT300_ALERT_4_G Atom Site Occupancy of H62D Constrained at 0.25 Check
PLAT300_ALERT_4_G Atom Site Occupancy of H62E Constrained at 0.25 Check
PLAT300_ALERT_4_G Atom Site Occupancy of H62F Constrained at 0.25 Check
PLAT300_ALERT_4_G Atom Site Occupancy of H63D Constrained at 0.25 Check
PLAT300_ALERT_4_G Atom Site Occupancy of H63E Constrained at 0.25 Check
PLAT300_ALERT_4_G Atom Site Occupancy of H63F Constrained at 0.25 Check
PLAT300_ALERT_4_G Atom Site Occupancy of H69D Constrained at 0.15 Check
PLAT300_ALERT_4_G Atom Site Occupancy of H69E Constrained at 0.15 Check
PLAT300_ALERT_4_G Atom Site Occupancy of H69F Constrained at 0.15 Check
PLAT300_ALERT_4_G Atom Site Occupancy of H70D Constrained at 0.15 Check
PLAT300_ALERT_4_G Atom Site Occupancy of H70E Constrained at 0.15 Check
PLAT300_ALERT_4_G Atom Site Occupancy of H70F Constrained at 0.15 Check
PLAT300_ALERT_4_G Atom Site Occupancy of H71C Constrained at 0.15 Check
PLAT300_ALERT_4_G Atom Site Occupancy of H71D Constrained at 0.15 Check
PLAT300_ALERT_4_G Atom Site Occupancy of H74D Constrained at 0.15 Check
PLAT300_ALERT_4_G Atom Site Occupancy of H74E Constrained at 0.15 Check
PLAT300_ALERT_4_G Atom Site Occupancy of H74F Constrained at 0.15 Check
PLAT300_ALERT_4_G Atom Site Occupancy of H75D Constrained at 0.15 Check
PLAT300_ALERT_4_G Atom Site Occupancy of H75E Constrained at 0.15 Check
PLAT300_ALERT_4_G Atom Site Occupancy of H75F Constrained at 0.15 Check
PLAT300_ALERT_4_G Atom Site Occupancy of C10L Constrained at 0.5 Check
PLAT300_ALERT_4_G Atom Site Occupancy of C11L Constrained at 0.5 Check
PLAT300_ALERT_4_G Atom Site Occupancy of C12L Constrained at 0.5 Check
PLAT300_ALERT_4_G Atom Site Occupancy of C13L Constrained at 0.5 Check

```
PLAT300_ALERT_4_G Atom Site Occupancy of C14L       Constrained at        0.5 Check
PLAT300_ALERT_4_G Atom Site Occupancy of C15L       Constrained at        0.5 Check
PLAT300_ALERT_4_G Atom Site Occupancy of C16L       Constrained at        0.5 Check
PLAT300_ALERT_4_G Atom Site Occupancy of C17L       Constrained at        0.5 Check
PLAT300_ALERT_4_G Atom Site Occupancy of C18L       Constrained at        0.5 Check
PLAT300_ALERT_4_G Atom Site Occupancy of H10L       Constrained at        0.5 Check
PLAT300_ALERT_4_G Atom Site Occupancy of H12L       Constrained at        0.5 Check
PLAT300_ALERT_4_G Atom Site Occupancy of H14L       Constrained at        0.5 Check
PLAT300_ALERT_4_G Atom Site Occupancy of H16A       Constrained at        0.5 Check
PLAT300_ALERT_4_G Atom Site Occupancy of H16B       Constrained at        0.5 Check
PLAT300_ALERT_4_G Atom Site Occupancy of H16C       Constrained at        0.5 Check
PLAT300_ALERT_4_G Atom Site Occupancy of H17A       Constrained at        0.5 Check
PLAT300_ALERT_4_G Atom Site Occupancy of H17B       Constrained at        0.5 Check
PLAT300_ALERT_4_G Atom Site Occupancy of H17C       Constrained at        0.5 Check
PLAT300_ALERT_4_G Atom Site Occupancy of H18A       Constrained at        0.5 Check
PLAT300_ALERT_4_G Atom Site Occupancy of H18B       Constrained at        0.5 Check
PLAT300_ALERT_4_G Atom Site Occupancy of H18C       Constrained at        0.5 Check
PLAT301_ALERT_3_G Main Residue  Disorder .............(Resd  1  )        21% Note
PLAT302_ALERT_4_G Anion/Solvent/Minor-Residue Disorder (Resd  3  )       100% Note
PLAT304_ALERT_4_G Non-Integer Number of Atoms in ..... (Resd  3  )      10.50 Check
PLAT333_ALERT_2_G Large Aver C6-Ring C-C Dist C12      -C14      .       1.43 Ang.
PLAT333_ALERT_2_G Large Aver C6-Ring C-C Dist C33      -C38      .       1.43 Ang.
PLAT412_ALERT_2_G Short Intra XH3 .. XHn     H9       ..H53B     .       2.09 Ang.
                                                        x,y,z  =     1_555 Check
PLAT412_ALERT_2_G Short Intra XH3 .. XHn     H11      ..H54C     .       2.09 Ang.
                                                        x,y,z  =     1_555 Check
PLAT412_ALERT_2_G Short Intra XH3 .. XHn     H29      ..H69C     .       2.08 Ang.
                                                        x,y,z  =     1_555 Check
PLAT412_ALERT_2_G Short Intra XH3 .. XHn     H45A     ..H50C     .       1.96 Ang.
                                                        x,y,z  =     1_555 Check
PLAT412_ALERT_2_G Short Intra XH3 .. XHn     H46A     ..H49C     .       2.12 Ang.
                                                        x,y,z  =     1_555 Check
PLAT412_ALERT_2_G Short Intra XH3 .. XHn     H73B     ..H75E     .       2.04 Ang.
                                                        x,y,z  =     1_555 Check
PLAT412_ALERT_2_G Short Intra XH3 .. XHn     H73B     ..H75F     .       1.87 Ang.
                                                        x,y,z  =     1_555 Check
PLAT720_ALERT_4_G Number of Unusual/Non-Standard Labels .........          9 Note
PLAT773_ALERT_2_G Check long C-C Bond in CIF: C56      --C58A            1.75 Ang.
PLAT789_ALERT_4_G Atoms with Negative _atom_site_disorder_group  #        21 Check
PLAT860_ALERT_3_G Number of Least-Squares Restraints ............        223 Note
PLAT912_ALERT_4_G Missing # of FCF Reflections Above STh/L=  0.600       397 Note
PLAT941_ALERT_3_G Average HKL Measurement Multiplicity ..........        3.0 Low
PLAT965_ALERT_2_G The SHELXL WEIGHT Optimisation has not Converged    Please Check
PLAT978_ALERT_2_G Number C-C Bonds with Positive Residual Density.         4 Info
```

---

   0 **ALERT level A** = Most likely a serious problem - resolve or explain
   1 **ALERT level B** = A potentially serious problem, consider carefully
  18 **ALERT level C** = Check. Ensure it is not caused by an omission or oversight
 167 **ALERT level G** = General information/check it is not something unexpected

   3 ALERT type 1 CIF construction/syntax error, inconsistent or missing data
  23 ALERT type 2 Indicator that the structure model may be wrong or deficient
  11 ALERT type 3 Indicator that the structure quality may be low
 149 ALERT type 4 Improvement, methodology, query or suggestion
   0 ALERT type 5 Informative message, check

It is advisable to attempt to resolve as many as possible of the alerts in all categories. Often the minor alerts point to easily fixed oversights, errors and omissions in your CIF or refinement strategy, so attention to these fine details can be worthwhile. In order to resolve some of the more serious problems it may be necessary to carry out additional measurements or structure refinements. However, the purpose of your study may justify the reported deviations and the more serious of these should normally be commented upon in the discussion or experimental section of a paper or in the "special_details" fields of the CIF. checkCIF was carefully designed to identify outliers and unusual parameters, but every test has its limitations and alerts that are not important in a particular case may appear. Conversely, the absence of alerts does not guarantee there are no aspects of the results needing attention. It is up to the individual to critically assess their own results and, if necessary, seek expert advice.

Publication of your CIF in IUCr journals

A basic structural check has been run on your CIF. These basic checks will be run on all CIFs submitted for publication in IUCr journals (*Acta Crystallographica*, *Journal of Applied Crystallography*, *Journal of Synchrotron Radiation*); however, if you intend to submit to *Acta Crystallographica Section C* or *E* or *IUCrData*, you should make sure that full publication checks are run on the final version of your CIF prior to submission.

Publication of your CIF in other journals

Please refer to the *Notes for Authors* of the relevant journal for any special instructions relating to CIF submission.

PLATON version of 18/12/2021; check.def file version of 18/12/2021

Datablock hbpo - ellipsoid plot

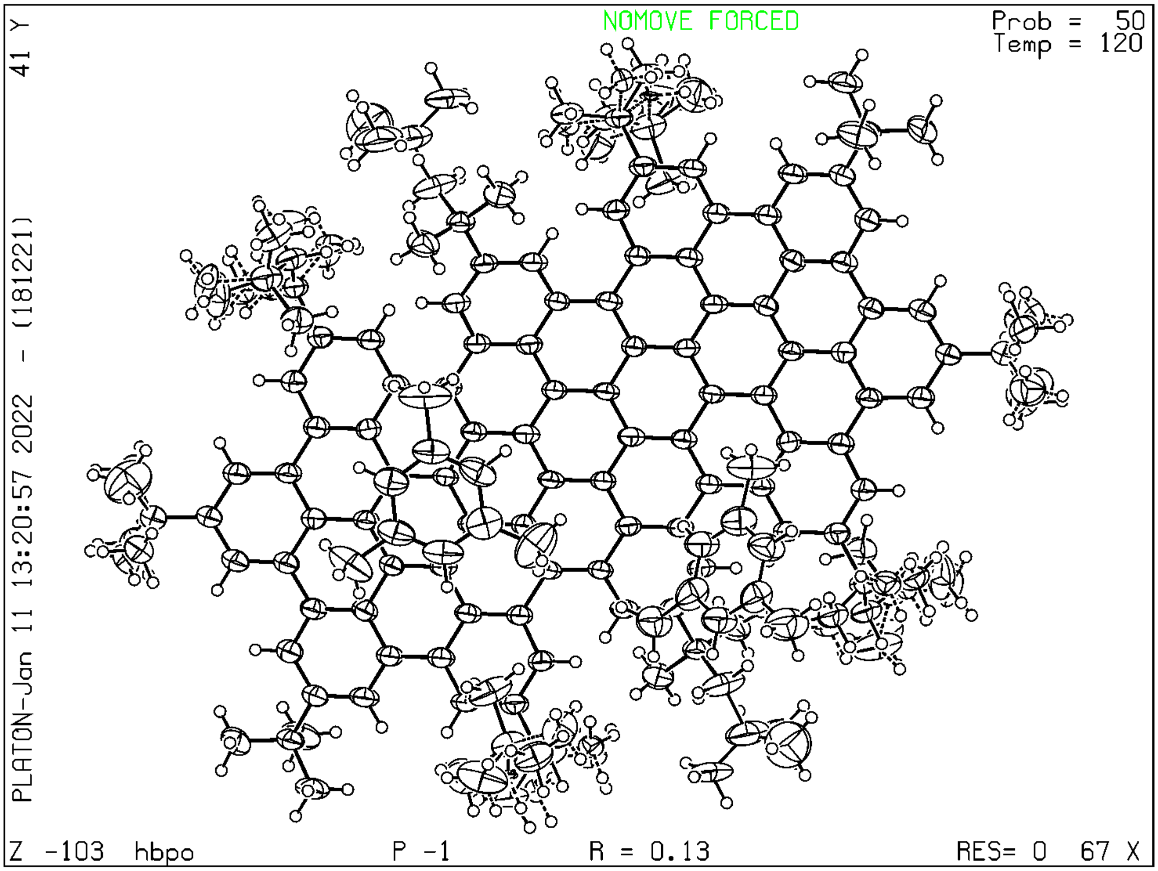

Supplement: Supplementary file 1 — Supporting Information [file ANIE-61-0-s001.pdf]
