# Supplementary material for: Cove‐Edged Hexa‐peri‐hexabenzo‐bis‐peri‐octacene: Molecular Conformations and Amplified Spontaneous Emission
Source: Angew Chem Int Ed Engl. 2022 Mar 2;61(18):e202201088. doi: 10.1002/anie.202201088 (PMC9311809; doi:10.1002/anie.202201088)

checkCIF/PLATON report

Structure factors have been supplied for datablock(s) cq87

THIS REPORT IS FOR GUIDANCE ONLY. IF USED AS PART OF A REVIEW PROCEDURE FOR PUBLICATION, IT SHOULD NOT REPLACE THE EXPERTISE OF AN EXPERIENCED CRYSTALLOGRAPHIC REFEREE.

No syntax errors found. CIF dictionary Interpreting this report

Datablock: cq87

```
Bond precision:  C-C = 0.0129 A          Wavelength=1.54178

Cell:           a=23.1399(10)      b=23.7733(11)     c=26.3841(11)
                alpha=78.775(3)    beta=71.015(3)    gamma=81.423(4)
Temperature:    120 K

                Calculated                Reported
Volume          13404.4(11)               13404.4(11)
Space group     P -1                      P -1
Hall group      -P 1                      -P 1
Moiety formula  C146 H150 [+ solvent]     C146 H150 + [SOLVENT]
Sum formula     C146 H150 [+ solvent]     C146 H150 + [SOLVENT]
Mr              1904.67                   1904.65
Dx,g cm-3       0.944                     0.944
Z               4                         4
Mu (mm-1)       0.395                     0.395
F000            4104.0                    4104.0
F000'           4114.08
h,k,lmax        27,28,31                  26,27,31
Nref            48952                     45893
Tmin,Tmax       0.914,0.984
Tmin'           0.910

Correction method= Not given

Data completeness= 0.938          Theta(max)= 68.073

R(reflections)= 0.2072( 20398)    wR2(reflections)= 0.5807( 45893)

S = 1.883                  Npar= 2030
```

The following ALERTS were generated. Each ALERT has the format
test-name_ALERT_alert-type_alert-level.
Click on the hyperlinks for more details of the test.

Alert level A

```
PLAT082_ALERT_2_A High R1 Value ..................................       0.21 Report
```

Author Response: poor diffracting crystal structure only for proof of the synthesis

```
PLAT084_ALERT_3_A High wR2 Value (i.e. > 0.25) ...................       0.58 Report
```

Author Response: poor diffracting crystal structure only for proof of the synthesis

```
PLAT201_ALERT_2_A Isotropic non-H Atoms in Main Residue(s) .......        120 Report
              C87       C88       C89       C90       C91                 etc.
```

Author Response: side groups highly disordered

```
PLAT934_ALERT_3_A Number of (Iobs-Icalc)/Sigma(W) > 10 Outliers ..         24 Check
```

Author Response: influence of the solvence (Platon SQUEEZE)

Alert level B

```
PLAT029_ALERT_3_B _diffrn_measured_fraction_theta_full value Low .      0.944 Why?
```

Author Response: geometrical limitation of the instrument

```
PLAT097_ALERT_2_B Large Reported Max.  (Positive) Residual Density       0.88 eA-3
PLAT241_ALERT_2_B High   'MainMol' Ueq as Compared to Neighbors of       C326 Check
```

Author Response: disordered t-butyl groups

```
PLAT242_ALERT_2_B Low    'MainMol' Ueq as Compared to Neighbors of       C115 Check
```

Author Response: disordered aliphatic chain

```
PLAT242_ALERT_2_B Low    'MainMol' Ueq as Compared to Neighbors of       C119 Check
```

Author Response: disordered aliphatic chain

```
PLAT242_ALERT_2_B Low    'MainMol' Ueq as Compared to Neighbors of       C135 Check
```

Author Response: disordered aliphatic chain

```
PLAT242_ALERT_2_B Low    'MainMol' Ueq as Compared to Neighbors of       C323 Check
```

Author Response: disordered aliphatic chain

```
PLAT242_ALERT_2_B Low    'MainMol' Ueq as Compared to Neighbors of       C327 Check
```

Author Response: disordered aliphatic chain

```
PLAT242_ALERT_2_B Low    'MainMol' Ueq as Compared to Neighbors of       C331 Check
```

Author Response: disordered aliphatic chain

```
PLAT242_ALERT_2_B Low    'MainMol' Ueq as Compared to Neighbors of       C335 Check
```

Author Response: disordered aliphatic chain

```
PLAT340_ALERT_3_B Low Bond Precision on  C-C Bonds ...............    0.01293 Ang.
```

Author Response: low intensity, high disorder, solvent could not be located

```
PLAT360_ALERT_2_B Short  C(sp3)-C(sp3) Bond  C119     - C121     .       1.32 Ang.
```

Author Response: disordered t-butyl group

Alert level C

```
DIFMN02_ALERT_2_C  The minimum difference density is < -0.1*ZMAX*0.75
            _refine_diff_density_min given =     -0.531
            Test value =     -0.450
DIFMN03_ALERT_1_C  The minimum difference density is < -0.1*ZMAX*0.75
            The relevant atom site should be identified.
DIFMX02_ALERT_1_C  The maximum difference density is > 0.1*ZMAX*0.75
            The relevant atom site should be identified.
PLAT026_ALERT_3_C Ratio Observed / Unique Reflections (too) Low ..        44% Check
PLAT098_ALERT_2_C Large Reported Min.  (Negative) Residual Density      -0.53 eA-3
PLAT220_ALERT_2_C NonSolvent   Resd 1  C   Ueq(max)/Ueq(min) Range        5.3 Ratio
PLAT220_ALERT_2_C NonSolvent   Resd 2  C   Ueq(max)/Ueq(min) Range        5.6 Ratio
PLAT222_ALERT_3_C NonSolvent Resd 1  H   Uiso(max)/Uiso(min) Range        5.8 Ratio
PLAT222_ALERT_3_C NonSolvent Resd 2  H   Uiso(max)/Uiso(min) Range        6.3 Ratio
PLAT241_ALERT_2_C High   'MainMol' Ueq as Compared to Neighbors of        C47 Check
```

Author Response: disordered t-butyl groups

```
PLAT241_ALERT_2_C High   'MainMol' Ueq as Compared to Neighbors of        C64 Check
```

Author Response: disordered t-butyl groups

```
PLAT241_ALERT_2_C High   'MainMol' Ueq as Compared to Neighbors of        C65 Check
```

Author Response: disordered t-butyl groups

```
PLAT241_ALERT_2_C High   'MainMol' Ueq as Compared to Neighbors of       C276 Check
```

Author Response: disordered t-butyl groups

```
PLAT241_ALERT_2_C High   'MainMol' Ueq as Compared to Neighbors of       C334 Check
```

Author Response: disordered t-butyl groups

```
PLAT242_ALERT_2_C Low    'MainMol' Ueq as Compared to Neighbors of        C66 Check
```

Author Response: disordered aliphatic chain

```
PLAT242_ALERT_2_C Low    'MainMol' Ueq as Compared to Neighbors of        C91 Check
```

Author Response: disordered aliphatic chain

```
PLAT242_ALERT_2_C Low    'MainMol' Ueq as Compared to Neighbors of       C103 Check
```

Author Response: disordered aliphatic chain

```
PLAT242_ALERT_2_C Low    'MainMol' Ueq as Compared to Neighbors of       C107 Check
```

Author Response: disordered aliphatic chain

```
PLAT242_ALERT_2_C Low    'MainMol' Ueq as Compared to Neighbors of       C111 Check
```

Author Response: disordered aliphatic chain

```
PLAT242_ALERT_2_C Low    'MainMol' Ueq as Compared to Neighbors of       C127 Check
```

Author Response: disordered aliphatic chain

```
PLAT242_ALERT_2_C Low    'MainMol' Ueq as Compared to Neighbors of       C134 Check
```

Author Response: disordered aliphatic chain

```
PLAT242_ALERT_2_C Low    'MainMol' Ueq as Compared to Neighbors of       C139 Check
```

Author Response: disordered aliphatic chain

```
PLAT242_ALERT_2_C Low    'MainMol' Ueq as Compared to Neighbors of       C143 Check
```

Author Response: disordered aliphatic chain

```
PLAT242_ALERT_2_C Low    'MainMol' Ueq as Compared to Neighbors of       C287 Check
```

Author Response: disordered aliphatic chain

```
PLAT242_ALERT_2_C Low    'MainMol' Ueq as Compared to Neighbors of       C291 Check
```

Author Response: disordered aliphatic chain

```
PLAT242_ALERT_2_C Low    'MainMol' Ueq as Compared to Neighbors of       C307 Check
```

Author Response: disordered aliphatic chain

```
PLAT242_ALERT_2_C Low    'MainMol' Ueq as Compared to Neighbors of       C315 Check
```

Author Response: disordered aliphatic chain

```
PLAT242_ALERT_2_C Low    'MainMol' Ueq as Compared to Neighbors of       C319 Check
```

Author Response: disordered aliphatic chain

```
PLAT242_ALERT_2_C Low    'MainMol' Ueq as Compared to Neighbors of       C343 Check
```

Author Response: disordered aliphatic chain

```
PLAT250_ALERT_2_C Large U3/U1 Ratio for Average U(i,j) Tensor ....        2.1 Note
PLAT360_ALERT_2_C Short  C(sp3)-C(sp3) Bond  C131     - C133     .       1.38 Ang.
```

Author Response: disordered t-butyl group

```
PLAT360_ALERT_2_C Short  C(sp3)-C(sp3) Bond  C291     - C294     .       1.39 Ang.
```

Author Response: disordered t-butyl group

```
PLAT361_ALERT_2_C Long   C(sp3)-C(sp3) Bond  C119     - C120     .       1.66 Ang.
PLAT361_ALERT_2_C Long   C(sp3)-C(sp3) Bond  C135     - C137     .       1.65 Ang.
PLAT361_ALERT_2_C Long   C(sp3)-C(sp3) Bond  C334     - C335     .       1.65 Ang.
PLAT361_ALERT_2_C Long   C(sp3)-C(sp3) Bond  C335     - C336     .       1.70 Ang.
PLAT361_ALERT_2_C Long   C(sp3)-C(sp3) Bond  C335     - C337     .       1.68 Ang.
PLAT361_ALERT_2_C Long   C(sp3)-C(sp3) Bond  C335     - C338     .       1.67 Ang.
PLAT410_ALERT_2_C Short Intra H...H Contact  H6       ..H14      .       1.97 Ang.
                                                      x,y,z  =      1_555 Check
PLAT410_ALERT_2_C Short Intra H...H Contact  H10      ..H27      .       1.97 Ang.
                                                      x,y,z  =      1_555 Check
PLAT410_ALERT_2_C Short Intra H...H Contact  H10Q     ..H29      .       1.96 Ang.
                                                      x,y,z  =      1_555 Check
PLAT410_ALERT_2_C Short Intra H...H Contact  H65      ..H76      .       1.97 Ang.
                                                      x,y,z  =      1_555 Check
PLAT410_ALERT_2_C Short Intra H...H Contact  H202     ..H208     .       1.97 Ang.
                                                      x,y,z  =      1_555 Check
PLAT410_ALERT_2_C Short Intra H...H Contact  H210     ..H227     .       1.96 Ang.
                                                      x,y,z  =      1_555 Check
```

```
PLAT412_ALERT_2_C Short Intra XH3 .. XHn     H10D     ..H96B     .       1.85 Ang.
                                                      x,y,z  =     1_555 Check
PLAT412_ALERT_2_C Short Intra XH3 .. XHn     H11F     ..H11S     .       1.87 Ang.
                                                      x,y,z  =     1_555 Check
PLAT412_ALERT_2_C Short Intra XH3 .. XHn     H11H     ..H11Q     .       1.88 Ang.
                                                      x,y,z  =     1_555 Check
PLAT412_ALERT_2_C Short Intra XH3 .. XHn     H13D     ..H13Q     .       1.81 Ang.
                                                      x,y,z  =     1_555 Check
PLAT412_ALERT_2_C Short Intra XH3 .. XHn     H13I     ..H13J     .       1.89 Ang.
                                                      x,y,z  =     1_555 Check
PLAT905_ALERT_3_C Negative K value in the Analysis of Variance ...     -0.652 Report
PLAT906_ALERT_3_C Large K Value in the Analysis of Variance ......      2.063 Check
PLAT911_ALERT_3_C Missing FCF Refl Between Thmin & STh/L=    0.600       2468 Report
PLAT918_ALERT_3_C Reflection(s) with I(obs) much Smaller I(calc) .        128 Check
PLAT923_ALERT_1_C S    Values in the CIF and FCF Differ by .......      0.012 Check
PLAT977_ALERT_2_C Check Negative Difference Density on H13E             -0.37 eA-3
PLAT977_ALERT_2_C Check Negative Difference Density on H30L             -0.33 eA-3
```

Alert level G

```
CELLZ01_ALERT_1_G Difference between formula and atom_site contents detected.
CELLZ01_ALERT_1_G ALERT: Large difference may be due to a
            symmetry error - see SYMMG tests
           From the CIF: _cell_formula_units_Z    4
           From the CIF: _chemical_formula_sum  C146 H150 + [solvent]
           TEST: Compare cell contents of formula and atom_site data

           atom    Z*formula  cif sites diff
           C        584.00    584.00    0.00
           H        600.00    600.00    0.00
           +          4.00      0.00    4.00
           [solv      4.00      0.00    4.00
PLAT002_ALERT_2_G Number of Distance or Angle Restraints on AtSite         10 Note
PLAT176_ALERT_4_G The CIF-Embedded .res File Contains SADI Records          2 Report
PLAT187_ALERT_4_G The CIF-Embedded .res File Contains RIGU Records          2 Report
PLAT333_ALERT_2_G Large Aver C6-Ring C-C Dist C4       -C20      .       1.43 Ang.
PLAT333_ALERT_2_G Large Aver C6-Ring C-C Dist C11      -C26      .       1.43 Ang.
PLAT333_ALERT_2_G Large Aver C6-Ring C-C Dist C23      -C81      .       1.43 Ang.
PLAT333_ALERT_2_G Large Aver C6-Ring C-C Dist C58      -C61      .       1.42 Ang.
PLAT333_ALERT_2_G Large Aver C6-Ring C-C Dist C203     -C207     .       1.43 Ang.
PLAT333_ALERT_2_G Large Aver C6-Ring C-C Dist C204     -C220     .       1.43 Ang.
PLAT333_ALERT_2_G Large Aver C6-Ring C-C Dist C211     -C226     .       1.43 Ang.
PLAT333_ALERT_2_G Large Aver C6-Ring C-C Dist C223     -C281     .       1.44 Ang.
PLAT333_ALERT_2_G Large Aver C6-Ring C-C Dist C252     -C255     .       1.42 Ang.
PLAT333_ALERT_2_G Large Aver C6-Ring C-C Dist C255     -C260     .       1.42 Ang.
PLAT333_ALERT_2_G Large Aver C6-Ring C-C Dist C258     -C261     .       1.43 Ang.
PLAT343_ALERT_2_G Unusual sp3      Angle Range in Main Residue for       C314 Check
PLAT343_ALERT_2_G Unusual sp3      Angle Range in Main Residue for       C326 Check
PLAT606_ALERT_4_G Solvent Accessible VOID(S) in Structure ........          ! Info
PLAT860_ALERT_3_G Number of Least-Squares Restraints .............       1884 Note
PLAT869_ALERT_4_G ALERTS Related to the Use of SQUEEZE Suppressed           ! Info
PLAT910_ALERT_3_G Missing # of FCF Reflection(s) Below Theta(Min).          3 Note
PLAT912_ALERT_4_G Missing # of FCF Reflections Above STh/L=  0.600        323 Note
PLAT913_ALERT_3_G Missing # of Very Strong Reflections in FCF ....          3 Note
PLAT941_ALERT_3_G Average HKL Measurement Multiplicity ...........        2.6 Low
PLAT978_ALERT_2_G Number C-C Bonds with Positive Residual Density.          0 Info
PLAT992_ALERT_5_G Repd & Actual _reflns_number_gt Values Differ by          2 Check
```

---

4 **ALERT level A** = Most likely a serious problem - resolve or explain
12 **ALERT level B** = A potentially serious problem, consider carefully
56 **ALERT level C** = Check. Ensure it is not caused by an omission or oversight

```
27 ALERT level G = General information/check it is not something unexpected

 5 ALERT type 1 CIF construction/syntax error, inconsistent or missing data
73 ALERT type 2 Indicator that the structure model may be wrong or deficient
15 ALERT type 3 Indicator that the structure quality may be low
 5 ALERT type 4 Improvement, methodology, query or suggestion
 1 ALERT type 5 Informative message, check
```

It is advisable to attempt to resolve as many as possible of the alerts in all categories. Often the minor alerts point to easily fixed oversights, errors and omissions in your CIF or refinement strategy, so attention to these fine details can be worthwhile. In order to resolve some of the more serious problems it may be necessary to carry out additional measurements or structure refinements. However, the purpose of your study may justify the reported deviations and the more serious of these should normally be commented upon in the discussion or experimental section of a paper or in the "special_details" fields of the CIF. checkCIF was carefully designed to identify outliers and unusual parameters, but every test has its limitations and alerts that are not important in a particular case may appear. Conversely, the absence of alerts does not guarantee there are no aspects of the results needing attention. It is up to the individual to critically assess their own results and, if necessary, seek expert advice.

Publication of your CIF in IUCr journals

A basic structural check has been run on your CIF. These basic checks will be run on all CIFs submitted for publication in IUCr journals (*Acta Crystallographica*, *Journal of Applied Crystallography*, *Journal of Synchrotron Radiation*); however, if you intend to submit to *Acta Crystallographica Section C* or *E* or *IUCrData*, you should make sure that full publication checks are run on the final version of your CIF prior to submission.

Publication of your CIF in other journals

Please refer to the *Notes for Authors* of the relevant journal for any special instructions relating to CIF submission.

PLATON version of 18/09/2020; check.def file version of 20/08/2020

Datablock cq87 - ellipsoid plot

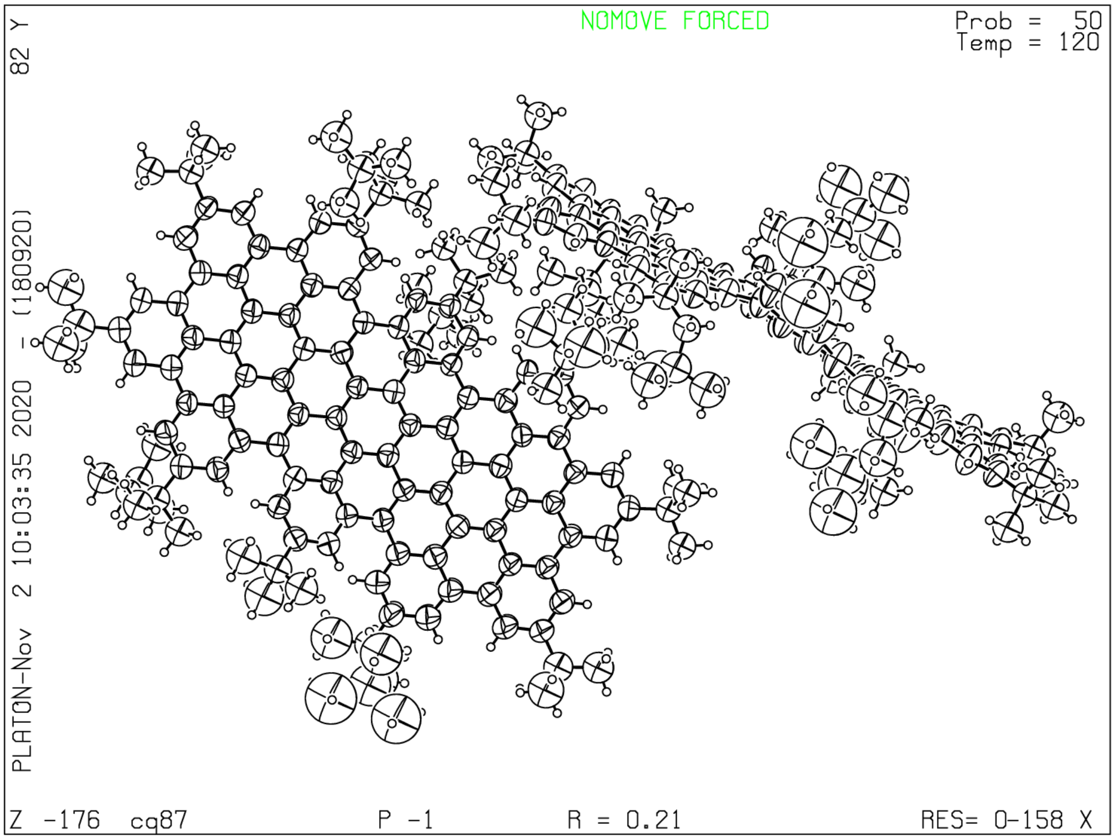

Supplement: Supplementary file 2 — Supporting Information [file ANIE-61-0-s004.pdf]
